# Genes targeted by the Hedgehog-signaling pathway can be regulated by Estrogen related receptor β

**DOI:** 10.1186/s12867-015-0047-3

**Published:** 2015-11-23

**Authors:** Yuan Lu, Jilong Li, Jianlin Cheng, Dennis B. Lubahn

**Affiliations:** Department of Biochemistry, University of Missouri, Columbia, MO 65211 USA; MU Center for Botanical Interaction Studies, University of Missouri, Columbia, MO 65211 USA; Computer Science Department, University of Missouri, Columbia, MO 65211 USA; Informatics Institute, University of Missouri, Columbia, MO 65211 USA; Xiphophorus Genetic Stock Center, Texas State University, San Marcos, TX 78666 USA

**Keywords:** Estrogen related receptor, Hedgehog signaling pathway, Gene expression profiling, RNA-Seq, Gene interactions

## Abstract

**Background:**

Nuclear receptor family member, Estrogen related receptor β, and the Hedgehog signal transduction pathway are both reported to relate to tumorigenesis and induced pluripotent stem cell reprogramming. We hypothesize that Estrogen related receptor β can modulate the Hedgehog signaling pathway and affect Hedgehog driven downstream gene expression.

**Results:**

We established an estrogen related receptor β-expressing Hedgehog-responsive NIH3T3 cell line by Esrrb transfection, and performed mRNA profiling using RNA-Seq after Hedgehog ligand conditioned medium treatment. Esrrb expression altered 171 genes, while Hedgehog signaling activation alone altered 339 genes. Additionally, estrogen related receptor β expression in combination with Hedgehog signaling activation affects a group of 109 Hedgehog responsive mRNAs, including *Hsd11b1*, *Ogn*, *Smoc2*, *Igf1, Pdcd4, Igfbp4, Stmn1, Hp, Hoxd8, Top2a, Tubb4b, Sfrp2, Saa3, Prl2c3* and *Dpt*.

**Conclusions:**

We conclude that Estrogen related receptor β is capable of interacting with Hh-signaling downstream targets. Our results suggest a new level of regulation of Hedgehog signaling by Estrogen related receptor β, and indicate modulation of Estrogen related receptor β can be a new strategy to regulate various functions driven by the Hedgehog signaling pathway.

**Electronic supplementary material:**

The online version of this article (doi:10.1186/s12867-015-0047-3) contains supplementary material, which is available to authorized users.

## Background

Hedgehog (Hh) signaling is a pivotal signaling pathway in embryonic pattern formation, stem cell/cancer stem cell self-renewal, as well as induced pluripotent stem cells induction [1–12]. An early study showed the Hh-signaling inhibitor, cyclopamine, is enriched in *Veratrum californicum.* This plant when consumed by pregnant sheep resulted in a midline differentiation defect in offspring [13–17]. Hh-signaling activation can lead to reprogramming by driving the expression of Bmi1, and the endogenous Smoothened activator, oxysterol, can facilitate reprogramming [8].

Similar to other core development related pathways, deregulated Hh-signaling due to the mutation or overexpression of pathway components and/or pathway ligand induces a variety types of cancers including basal cell carcinoma, medulloblastoma, bladder cancer, breast cancer, cervical cancer, liver cancer, colon cancer, prostate cancer, gastric cancer, pancreas cancer, head and neck cancer, lymphoma and non-small cell lung cancer [18–32]. The pivotal role of Hh-signaling in cancer development makes Hh-signaling an attractive target for drug development [33–36]. For example, the FDA in 2012 approved GDC-0449, an Hh pathway inhibitor targeting Smoothened for basal cell carcinoma treatment [33, 37, 38].

Hh signaling pathway is controlled by membrane proteins Patched (Ptch) and Smo. When there is no ligand bound to Ptch, Ptch inhibits Smo and keeps the downstream pathway inactivated. When Ptch binds to Hh ligand, the inhibition of Ptch on Smo is relieved and the Hh-signaling pathway is activated. One of the broadly accepted mechanisms of Hh-signaling target genes response is through the binding of Gli family transcription factors to Gli-binding sites in the regulatory sequence of Hh regulated genes.

Esrrb belongs to the nuclear receptor family [39–41]. It is important in early embryo development as genomic knock out of Esrrb is embryonic lethal due to the placenta deformation resulted from early differentiation of trophoblast stem cells [42]. Recent research showed that Esrrb was found to be a core reprogramming factor in inducing pluripotent stem cells (iPSC). C-myc and klf4 of Yamanaka factors (Oct4, Sox2, Klf4, c-Myc) can be replaced by Esrrb [43–46]. Esrrb was also reported to drive Sox2 transcription to induce reprogramming in a single cell reprogramming system, revealing its central role in differentiation [47]. In addition, Esrrb was found to play an important role in tumorigenesis in both in vitro and in vivo studies. It is down-regulated in prostate cancer and re-expression of Esrrb in prostate cancer cells inhibited cancer cell proliferation through tumor suppressor Cdkn1a/p21 induction [48, 49].

Esrrb has been reported to be constitutively active in the absence of a ligand and this is supported by the evidence that Esrrg, which shares over 80 % of its Ligand Binding Domain with Esrrb, has a transcriptional active conformation similar to E2-activated Estrogen Receptor [50–53]. Another explanation for this endogenous activity is that Esrrb binds to an unknown endogenous ligand. The Esrrb endogenous ligand hypothesis is supported by a report that culturing the cells with charcoal-stripped serum-containing medium can eliminate the transcriptional activity of Esrrb on SFRE/ERRE [54].

Knocking down Esrrb in mouse embryonic stem cells was shown to affect the transcription of several Hh-signaling pathway related genes, including *Gli2* and several Wnt family members, indicating Esrrb can potentially regulate Hh driven gene expression [55].

Although Gli transcription factors are relatively well known for transmitting Hh-signaling to target genes, other factors mediating the Hh-signaling activity are not well studied. For example, *Dner, Fbn2, Hsd11b1* and *Brak* are Hh responsive genes in fetal prostate, but overexpression of *Gli1* or *Gli2* cannot affect the transcription of these 4 genes. However, the expression of active Smo significantly increased the mRNA concentration of these genes [56], indicating there is at least one other mechanism accounting for the Hh-signaling target gene transcription regulation.

Due to the importance of both Esrrb and Hh-signaling in development and tumorigenesis, we hypothesized that Esrrb can regulate Hh-mediated transcription regulation and can serve as a regulator of Hh-signaling target genes. By employing mRNA profiling, we emphasized on the discovery of Esrrb-regulated Hh-signaling pathway-targeted genes and we report 109 genes that differentially respond to Hh-signaling activation with Esrrb present.

## Results

### Establishment of model cell lines

**Fig. 1:**
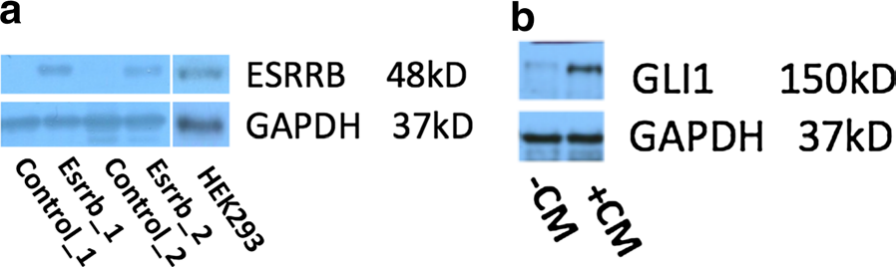
Characterization of model cell line. **a** Expression of Esrrb is confirmed by Western blot. Two replicates of Esrrb transfected cells showed successful expression of Esrrb protein and **b** 1 % Hh-CM treated NIH3T3-pc3.1 showed increased concentration of Gli1 protein

With the purpose of exploring whether Esrrb can regulate Hh-signaling targeted genes, we require a Hh-responsive cell line. NIH3T3 cells are commonly used as Hh-responsive cell line. Additionally, this cell line does not express Esrrb, making it a good model that provides clean background of Esrrb. To make the NIH3T3 cells Esrrb positive, we stably transfected Esrrb into NIH3T3 cells. The control vector transfected NIH3T3 cells (NIH3T3-pc3.1) have no Esrrb expressed as mentioned before. In contrast, NIH3T3 cells transfected with Esrrb expression vector (NIH3T3-Esrrb) have significantly increased Esrrb protein concentration (Fig. 1a). Compared to HEK293 cells, which have endogenous Esrrb expressed but lack of detectable Hh-signaling response, the concentration of overexpressed Esrrb in NIH3T3 cells attained a physiological relevant concentration (Fig. 1a).

### Hedgehog signaling and Esrrb regulated genes

**Fig. 2:**
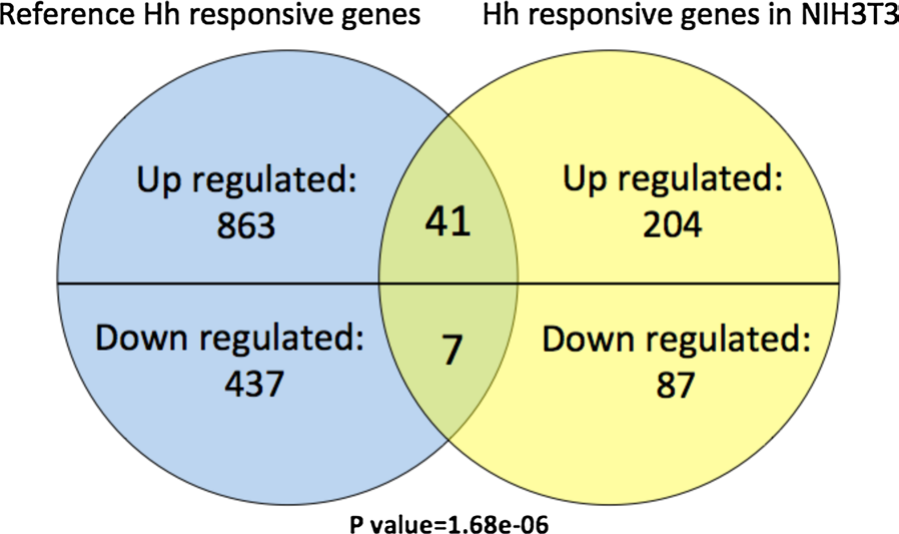
Hh-signaling target genes. Previously reported Hh-signaling targeted genes were retrieved, only the genes that have more than twofold change are kept. The Hh responsive genes from our assay were compared to the reported genes. *Venn diagram* shows known Hh-signaling genes with the Hh-signaling responsive genes from our study were compared. The 48 overlapped genes (enrichment score = 1.68e−06) contain 41 Hh-signaling up-regulated genes, and 7 down-regulated genes

**Fig. 3:**
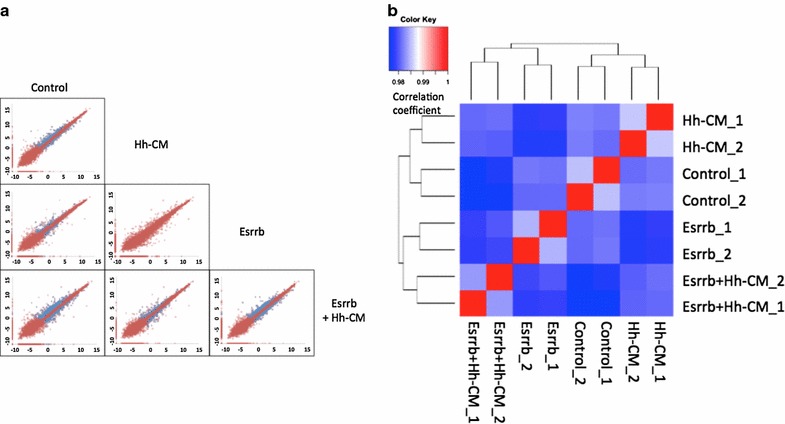
Pair-wise comparisons for differentially expressed genes within Control, Hedgehog treatment, Esrrb expression and Esrrb expression plus Hedgehog treatment. **a** *Scatter plot* of gene expression values in different conditions. 0.001 is added to the RPKM value of each transcript and resulted RPKM + 0.001 values are log_2_ transformed. For each *plot*, each point (*Xcondition1*, *Ycondition2*) on the plot represents the log_2_ (RPKM + 0.001) of the gene in indicated conditions. If a certain gene passed the different expression test, that gene is highlighted by *blue color*, otherwise is *red colored*. All pair-wise comparisons are plotted, except Hedgehog vs. Esrrb, which lacks biological meaning and **b** Spearman Ranking Correlation was used to analyze the similarity of mRNA profiles in control, Hh-signaling activation, Esrrb expression and Hh-signaling activation with Esrrb expression. Spearman ranking correlation coefficient was calculated and Hierarchical clustered. Correlation coefficient is *color coded* as indicated in the figure

To comprehensively characterize Hh-signaling driving mRNA changes in our system, we performed RNA-Seq analysis on the mRNA isolated from NIH3T3-pc3.1 cells treated with vehicle control or 1 % Hh ligand conditioned medium (Hh-CM). Hedgehog signaling pathway activation after Hh-CM treatment was confirmed by significantly increased concentration of Gli1 by westernblot (Fig. 1b). After the Hh-CM treatment, we distinguished a total of 339 (245 up-regulated, 94 down-regulated) altered mRNAs (Figs. 2, 3a, Additional file 1: Table S3). We collected published Hh-signaling target gene sets generated by activating the Hh-signaling pathway or Gli transcription factor overexpression/knock-down in different model cell lines or tissues as Ref. [56–63]. For all of the non-redundant 1348 genes from previous reports, we found 48 genes (enrichment *p* value = 1.68e–06) that overlapped and another 291 new Hh-signaling responsive genes (Fig. 2).

We also surveyed the gene expression in NIH3T3-Esrrb cells. 171 genes were differentially expressed by Esrrb expression (Additional file 1: Table S2; Additional file 2: Figure S1). We compared Esrrb-regulated genes to Hh-induced genes, and found there are 12 genes, *Fabp4, Phex, Ccl5, Tagln, Aldh1a7, Lmod1, Cesla, Igf1, Mafb, Steap4, Pfkfb3* and *Hlf*, which are up-regulated or down-regulated by either Hh ligand or Esrrb expression (Additional file 2: Figures S1,  S2, #1–8). The presence of these genes implies that Hh-signaling and Esrrb have functional overlaps in the regulation of these genes.

### Hh-signaling regulated genes in the presence of Esrrb

Next we treated NIH3T3-Esrrb cells with Hh-CM to test gene response. Esrrb expression with Hh-CM treatment led to the largest amount of altered mRNAs (Fig. 3a). Supportively, spearman ranking correlation of all mRNA profiles in four different conditions showed that each condition generates different mRNA profiles, and correlation between Hh-signaling pathway stimulation in the presence of Esrrb expression and the no Esrrb no Hh stimulation control resulted in the lowest correlation coefficient in all pairwise comparisons (Fig. 3b).

Theoretically, mRNA profiling from four different conditions (Control, Hh-CM, Esrrb, Esrrb + CM) generates six different differentially expression pairwise comparisons. Since the comparison “Hh-CM vs. Esrrb” lacks of apparent interest, only five comparisons were left (refer to “Methods”). Every gene can be differentially expressed or not in any of the five given comparisons, therefore the comparison results can contain 2^5^ different possibilities for each gene.

**Fig. 4:**
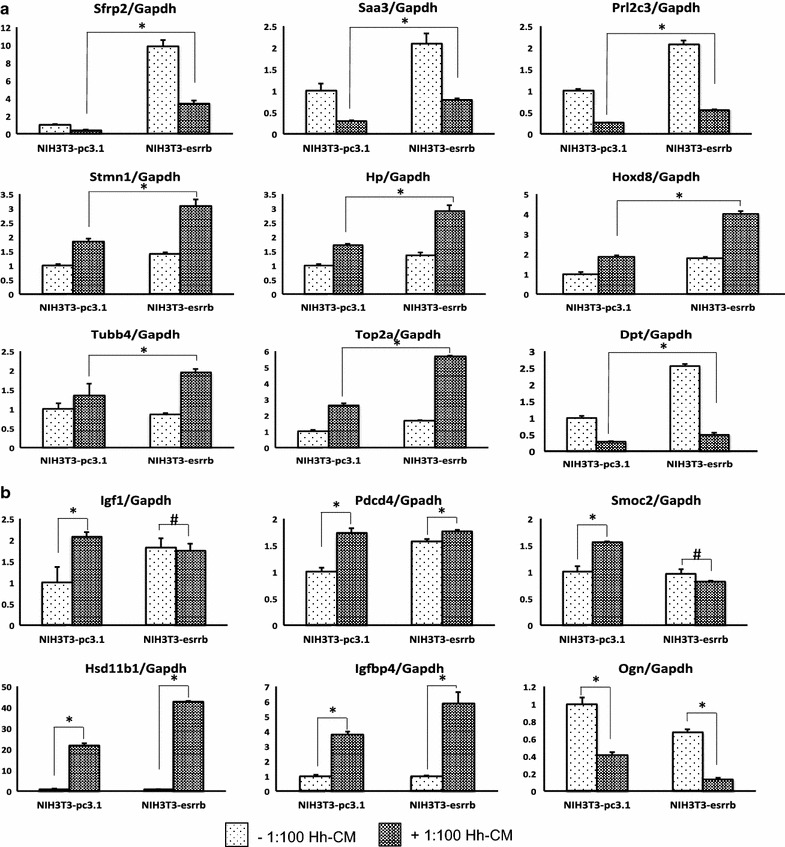
Quantitative PCR validation of the top 15 Hedgehog signaling differentially responsive mRNAs. Hh-signaling differentially responsive genes as determined by RNAseq were confirmed by qPCR after NIH3T3-pc3.1 and NIH3T3-Esrrb cells are treated with Hh-CM. Each mRNA concentration is normalized to housekeeping gene GAPDH, and further normalized to that ratio in NIH3T3-pc3.1. *Y-axis* represents relative fold change to no Esrrb expressed no Hh-CM treated control. **a** Esrrb altered the Hh-CM treatment response, due to a proportional additive effect from Esrrb expression. When Esrrb is expressed, the genetic response to Hh is different than the condition without Esrrb expressed, *Asterisk* means p < 0.01 and **b** genes respond to Hedgehog signaling better when Esrrb is expressed as well as genes losing Hh response when Esrrb is expressed, *Asterisk* means p < 0.01 *Number sign* means “not statistically significant”

We defined a group of genes as “Hh differentially response genes” as they response to Hh-CM when there is no Esrrb, while when Esrrb is expressed, their response to Hh-CM is further enhanced, depressed or lost. These genes will indicate Esrrb can regulate Hh-signaling pathway activity. To find Hh differentially response genes, we used computer assisted gene sorting (“Methods”, Additional file 2: Figure S2). The genes that fit our definition of “Hh differentially response” were found in groups #1, #2, #3, #4, #5, #9, #10 and #14 (Additional file 1: Table S3). Using the indicated filters of fold change and RPKM value described in “Methods”, we classified 109 genes that differentially respond to Hh-CM treatment when Esrrb is expressed (Additional file 2: Figure S2). We confirmed the concentration of 15 highly expressed mRNAs (*saa3, prl2c3, dpt, sfrp2, pdcd4, smoc2, igf1, stmn1, top2a, tubb4b, hp, hoxd8, igfbp4, hsd11b1, ogn*) by qPCR. Pearson correlation coefficients between RNA-Seq and qPCR are at least 0.9 (Additional file 2: Figure S4). Among tested mRNAs, we found that when Esrrb expressing cells are treated with Hh-CM, *Sfrp2* (secreted frizzle related protein 2), *Saa3* (serum amyloid A3), *Prl2c3* (prolactin 2A3), *Stmn1* (stathmin1), Hp (haptoglobin), *Hoxd8* (homeoboxD8), *Tubb4b* (tubulin beta 4B), *Top2a* (topoisomerase II alpha) and *Dpt* (dermatopontin) had different mRNA concentrations compared to Hh-CM treatment in cells without Esrrb. These differences are more likely due to a proportional additive effect of Esrrb expression on altered baseline expression of the mRNAs (Fig. 4a). In contrast, *Igf1* (Insulin-like growth factor 1), *Pdcd4* (programmed cell death 4) and *Smoc2* (SPARC related calcium binding 2) lost their response to Hh stimuli when Esrrb was present (Fig. 4b). On the other hand, *Hsd11b1* (hydoxysteroid 11 beta dehydrogenase 1), *Igfbp4* (insulin-like growth factor binding protein 4) and *Ogn* (osteoglycin) responded to Hh-CM better when Esrrb was expressed (Fig. 4b).

## Discussion

We distinguished 339 Hh-signaling altered mRNAs, among which are 48 known Hh-signaling target genes and 291 newly discovered targets (Fig. 3a, Additional file 1: Table S2). Most importantly, we characterized 109 genes that behave differently in response to Hh ligand in the presence versus the absence of Esrrb expression. This group of genes contains genes as *Igf1*, which respond to Hh ligand treatment in the absence of Esrrb, but when Esrrb is expressed, Hh ligand treatment cannot modify its transcription (Fig. 4b); genes as *Hoxd8*, which has differential expression in response to Hh stimulation when Esrrb is expressed, though this different response comes from the effect of Esrrb on basal level mRNA concentration (Fig. 4a). We also observed genes like *Hsd11b1* (Hh-CM vs. control: 21-fold; Esrrb + Hh-CM vs. Esrrb: 59 fold), *Igfbp4* (Hh-CM vs. control: 3.4-fold; Esrrb + Hh-CM vs. Esrrb: 7.6 fold), and *Ogn* (Hh-CM vs. control: 57 % inhibition; Esrrb + Hh-CM vs. Esrrb, 83 % inhibition), which indicate that Esrrb and the Hh-signaling pathway synergistically regulate these genes (Fig. 4b). These results demonstrated that Esrrb is capable of interacting with Hh-signaling pathway and potentially regulate Hh-signaling downstream targets.

In the canonical Hh-signaling pathway model, the activation of Smo transmits the Hh signal to Gli transcription factor through the activation and inactivation of several pathway components including Fused [30] and Suppressor of Fused (SuFu). However, the evidence that the transcription of *Hsd11b1*, *Fbn2* and *Brak* respond to active Smo transfection, but not Gli1 or Gli2 overexpression, indicates that Smo activation has a Gli-independent function in regulating gene expression [56]. In support of this hypothesis, we listed all the transcription factors; chromatin remodeling factors and transcription co-factors that response to Hh-signaling activation for future reference (Additional file 2: Figure S2).

There are 21 genes (group #25) that respond to Hh-signaling but only when Esrrb is present (Log2FC > 1 or Log2FC <−1) (Additional file 2: Figure S5). 20 of them are up-regulated and 1 is down-regulated. This indicates Esrrb has the ability to expand the transcription regulation of the Hh-signaling pathway.

Additionally, we found cortisol-cortisone converting enzyme Hsd11b1 is correlated to Hh-signaling activation from several reports in several model systems including fetal prostate, prostate cancer and embryonic fibroblast cell lines [56, 64], indicating cortisone converted from cortisol by Hsd11b1 may account for part of the Hh response gene profile. Surprisingly, we found the classic Glucocorticoid Receptor (GR) target gene, *Mt2*, along with 3 Hh-signaling activation inhibits all other genes that have Glucocorticoid Response Elements in their promoter regions, *Aldh1a7, Ankrd1* and *Ism1* are repressed in response to Hh treatment (Additional file 2: Figure S6). Although Esrrb does not change the expression of Hsd11b1, Hh treatment with Esrrb expression further increased the mRNA concentration of Hsd11b1, accompanied by statistically significant alterations in concentrations of *Mt2, Aldh1a7, Ankrd1* and *Ism1* (Additional file 2: Figure S6). Our discovery strongly supports the idea that metabolite(s) downstream of Smo can also be mediators of Hh-signaling responses. Interestingly, GR overexpression and the activation of its target genes are strongly associated with anti-androgen treatment in prostate cancer therapy. GR target genes overlap with those of the Androgen Receptor and have been determined to be involved in antiandrogen treatment enzalutamide resistance [65]. Inhibiting GR can restore enzalutamide sensitivity. Esrrb’s activity in increasing Hsd11b1 indirectly represses GR activity by potentially lowering the GR ligand cortisol, and thus activating Esrrb may lead to better response of antiandrogen treatment and eliminate or postpone the resistance.

## Conclusions

Our data provided useful reference marker genes for both Hh-signaling and Esrrb function. In addition, we also showed that Esrrb has a role in the regulation of Hh-signaling driven genes. The mechanism of Hh-signaling was also expanded and a new layer of regulation of the Hh pathway through Esrrb was revealed, which may lead to improved treatments in Hh-signaling driven diseases.

## Methods

### Conditional medium

Sonic Hedgehog conditioned medium (Hh-CM) is collected from cultured HEK293 cells carrying Sonic Hedgehog (Shh) N-terminus transgene, which was a gift from Dr. Phillip Beachy’s lab. Briefly, the Shh stable-transfected cells were maintained in Dulbecco’s Modified Eagal Medium (DMEM, Invitrogen, Grand Island, NY, USA) with 10 % Fetal Bovine Serum (FBS) until confluent, the medium was switched to DMEM with 0.2 % FBS after 2 days. The medium enriched with Shh-N terminus was collected 24 h later, and was filtered through 0.22 μM filter, and stored in −80° freezer [32].

### Cell lines and Expression vectors

All cell lines used in this study were obtained commercially, and we did not conduct any animal work to obtain them. Paracrine Hh-responsive mouse embryonic fibroblast cells NIH3T3 were obtained from American Type Culture Collection (ATCC, Cat. No. CRL-1658). NIH3T3 cells are cultured in DMEM supplement with 10 % Newborn Calf Serum (NBCS). The cells were transfected with pcDNA3.1 (Zeo+) empty vector (Promega, Madison, WI) as control, or pcDNA3.1 (Zeo+)-Esrrb expression vector using Fugene HD (Promega, Madison, WI), and were further selected by supplementing 150 μg/ml Zeocine (Invitrogen, Grand Island, NY) to the culture medium for 3 weeks. Two independent transfections were performed and established cells from each transfection were pooled together. Cells were cultured until confluent, and are treated with 1:100 diluted Hh-CM for 48 h, in phenol-red free DMEM supplement with 5 % NBCS. The Hh-CM treatment was controlled by the same dilution of medium cultured HEK293 cells that do not express Shh N-terminus transgene.

### Reverse transcriptase PCR and real time PCR

Total RNA was isolated and purified from two biological replicates of NIH3T3-pc3.1 and NIH3T3-Esrrb using RNeasy kit (Qiagen, Venlo, Netherlands) respectively. 1000 ng of total RNA was used to create cDNA libraries using Superscript III Reverse Transciptase (Invitrogen, Grand Island, NY) with random primers and oligodT. Quantitative PCR (qPCR) assays were carried out using SYBR GREEN iQ supermix (BioRad, Herculus, CA, USA) on ABI7500 system (Applied Biosytems, Foster City, CA, USA). Each qPCR assay was repeated 3 times. qPCR condition: 95°, 30 s; 60°, 40 s; 72°, 40 s. Primer sequences:

GAPDH (NM_008084), forward primer: AGCCTCGTCCCGTAGACAAAAT, reverse primer: CCGTGAGTGGAGTCATACTGGA;

Patched (NM_008957), forward primer: CTCTGGAGCAGATTTCCAAGG, reverse primer: TGCCGCAGTTCTTTTGAATG;

Gli1 (NM_010296), forward primer: GGAAGTCCTATTCACGCCTTGA, reverse primer: CAACCTTCTTGCTCACACATGTAAG;

Igf1 (NM_001111274), forward primer: TGAGTGGCTTCCCTTGGGGG, reverse primer: AGGTGTTGTTTTGTGGGTGGGGT;

Smoc2 (NM_022315), forward primer: GGAAGGAGCAGGGAAAGCAGATGAT, reverse primer: TGGGCTGCTTGGCTTCCTCAAG;

Pdcd4 (NM_001168491), forward primer: GGACACTCCTAGGGCACCGC, reverse primer: TCCGCTTCCCGCCTTTGGAC;

Stmn1 (NM_019641), forward primer: TCGGACCGAGCAGGGCTTTC, reverse primer: CCGAGGGCTGAGAATCAGCTCAA;

Hp (NM_017370), forward primer: GAGGCAGTGTGTGGGAAGCCC, reverse primer: GGTCAGCAGCCACTGGTCACT;

Ogn (NM_008760), forward primer: ACGACCTGGAATCTGTGCCTCC, reverse primer: TTGGATTGCCCTCCAGGCGA;

Hoxd8 (NM_008276), forward primer: TTCCCTGGATGAGACCACAAGCAGC, reverse primer: GTCTCTCCGTGAGGGCCAGAGT

Dpt (NM_019759), forward primer: TCAGTGCTGGATCGTGAGTGGC, reverse primer: ACTGGCGATCCCTTTCCACTGC;

Top2a (NM_011623), forward primer: CCCAGGGAAGCTCCATGTCGG, reverse primer: GGTTCCCTTTGGCGCAGCTC;

Igfbp4 (NM_010517), forward primer: GATCGTGGGGACACCTCGGG, reverse primer: GCGGGGTGACACTGTTTGGGG;

Tubb4b (NM_146116), forward primer: TGTTGGCAGAGCGTCGGTTGT, reverse primer: CGCTGATTACCTCCCAGAACTTGGC;

Hsd11b1 (NM_001044751): forward primer: CTGCCTGCCTGGGAGGTTGT, reverse primer: TCCCTGGAGCATTTCTGGTCTGAAC;

Sfrp2 (NM_009144): forward primer: GGCCACAGAGGAAGCTCCCAA, reverse primer: TCGGACACGCCGTTCAGCTT;

Saa3 (NM_011315): forward primer: ACAGCCAAAGATGGGTCCAGTTCA, reverse primer: ACAGCCTCTCTGGCATCGCTGA;

Prl2c3 (NM_011118): forward primer: AGCCAGGCTCACACACTATGCAG, reverse primer: CCCGTTCCGGACTGCGTTGA;

### Immunoblot

600,000 cells were plated in 6-well-plate. After 24 h growth in medium containing 10 % NBCS, the cells were treated with 1 % Hh-CM in phenol-red free DMEM with 5 % NBCS for 48 h. Cells were lysed by protein sample buffer (BioRad, Herculus, CA, USA) and boiled for 5 min at 95°. 20 μg of total protein was loaded on 12 % SDS-PAGE gel. Electrophoresis was then performed. The proteins were then transferred to nitrocellulose membrane. The membrane was block by Phosphate buffered saline (PBS) with 0.05 % Tween 20, 0.015 g/ml dry milk and 0.015 g/ml bovine serum albumin (BSA). The membrane was then incubated with 1:2000 diluted monoclonal anti-Gli1 mouse IgG (Cell signaling, Beverly, MA, Cat.No: L42B10), 1:2000 diluted monoclonal anti-Esrrb mouse IgG (R&D system, Cat. No: PP-H6705-00) and 1:2000 diluted polyclonal anti-GPADH rabbit IgG (Santa Cruz, Dallas, TX, Cat. No: sc-25777) overnight, washed, incubated with secondary antibodies diluted in PBS with 0.01 g/ml BSA. The chemoluminescence was generated by west-Dura (Promega, Madison, WI, USA) and recorded by X-ray film (Fisher Scientific, Pittsburg, PA, USA).

### Deep sequencing and data analysis

2500 ng total RNA from 2 biological replicates of each culture condition were extracted and purified, and then used to generate sequencing cDNA library using TruSeq Stranded mRNA Sample Preparation kits (Illumina, San Diego, CA). Eight samples were pooled in one lane and each sample was ligated to one specific barcoded aligner. cDNA libraries quality was determined by University of Missouri DNA core. Deep sequencing was performed by University of Missouri DNA core using Illumina HiSeq 2000. Around 18 million reads were generated in.fastq format for each sample (raw data and data repository accession number will be available upon manuscript acceptance). The sequencing reads were trimmed and filtered using FASTX-Toolkit (http://hannonlab.cshl.edu/fastx_toolkit), and mapped to UCSC mm9 genome using Bowtie2 and TopHat2 [66, 67]. Genome mapping results were submitted to Galaxy public server (galaxy.psu.edu) for gene expression value quantification. Gene expression value is represented as Reads Per Kilobase of transcript per Million mapped reads (RPKM) by Cufflink reference genome guided transcript assembly. Gene models from different experiment were merged together by Cuffmerge and all pair-wise comparisons of relative mRNA concentrations were analyzed by Cuffdiff. Differentially expressed genes are determined by False Discovery Rate adjusted p-value (q-value < 0.05), Log_2_Fold Change (Log_2_FC ≥ 1 or ≤−1).

### Gene sorting and Hh differentially response genes characterization

Five pairwise comparisons, (1) control vs. Hh-CM, (2) control vs. Esrrb, (3) control vs. Esrrb + Hh-CM, (4) Hh-CM vs. Esrrb + Hh-CM, (5) Esrrb vs. Esrrb + Hh-CM, but not Esrrb versus Hh-CM treatment, were made through differentially expressed gene analysis Cuffdiff, and test results are stored for each transcript [Yes (Y): q < 0.05, statistically significantly different; or No (N): q > 0.05, not significant]. Each gene is then sorted into 1 of the 32 groups based on each test result using an in-house R script (available upon request, Additional file 2: Figure S1). For each of the 16 groups of Hh responsive genes (control vs. Hh-CM, q < 0.05), a logic determination is made to filter out the groups that have pairwise comparison results against themselves (group #6, #7, #8, #12, #13, #15, #16) or groups with no real world interest (#11). Genes in the rest groups (#1, #2, #3, #4, #5, #9, #10 and #14) are for further data filter and analysis. For the genes that passed the Hh-CM vs. Esrrb + Hh-CM test, we further filtered out genes that have −0.5 < [(RPKM(Esrrb + Hh-CM) − RPKMHh-CM)/RPKMHh-CM] < 0.5, −0.5 < [(RPKM(Hh-CM) − RPKMcontrol)/RPKMcontrol] < 0.5, and −5 < [RPKM(Esrrb + Hh-CM) − RPKMHh-CM] < 5. For the genes that do not pass the Hh-CM vs. Esrrb + Hh-CM, the genes that have −0.5 < [(RPKM(Hh-CM) − RPKM(control))/RPKMcontrol] < 0.5, [(RPKM(Esrrb + Hh-CM) − RPKMHh-CM)/RPKMHh-CM] > 0.1 or <−0.1, −0.5 < [(RPKM(Esrrb) − RPKM(control))/RPKMcontrol] < 0.5 are filtered out. All genes that have passed the above tests are classified as Hh differentially response genes, and they respond to Hh-CM treatment differentially in the conditions with or without Esrrb expression.

### Gene ontology and KEGG pathway analysis

DAVID bioinformatics source 6.7 is used for gene ontology (GO) analysis and KEGG pathway analysis [68, 69]. The gene names from certain pairwise comparison result were submitted to DAVID server (http://david.abcc.ncifcrf.gov) and GO analysis were performed for biological process (BP). Minimum counts were set as default value (two counts) and maximum EASE score (p value) was set to 0.05. Same parameter was used for KEGG pathway analysis.

### Hh-signaling related genes enrichment analysis

Previous published Hh-signaling related gene sets generated by deep sequencing or microarray were retrieved (Refer to Additional file 1: Table S1 for specific experiment conditions and Hh-signaling targeted gene set descriptions). Briefly, only mRNAs altered more than twofold are included. Up-regulated and down-regulated mRNAs are sub-grouped and mRNA associated sequence identifications are all converted to gene symbols. Hh altered mRNAs in our gene set are compared to the previous reported mRNAs, and enrichment p value is calculated as previously described [70].

### Statistical analysis

Spearman Ranking Correlation is analyzed using R (version 3.0.2). RPKM value for each mRNA from both biological replicate in each condition is collected for correlation. The resulted pairwise correlation coefficients are stored in a matrix and Hierarchical clustering is created by R/Bioconductor (version 2.13) package Heatplus 2. Each Spearman correlation coefficient is color transformed for data visualization. qPCR experiments were repeated 3 times on two biological replicates. qPCR results were analyzed using t-test (p < 0.01). Hh differentially response mRNA concentrations measured by RNAseq and qPCR are correlated using Pearson method in R. Briefly, for each tested mRNA, qPCR tested relative concentration is normalized by the concentration of internal control GAPDH, and further normalized to control condition. RNA-seq generated RPKM values are normalized to the value from control. Correlation is then performed between the above two sets of normalized expression values.

## Availability of supporting data

The data sets supporting the results of this article are available in the NCBI-GEO repository, GSE71209 http://www.ncbi.nlm.nih.gov/geo/query/acc.cgi?acc=GSE71209.

## Additional files

10.1186/s12867-015-0047-3 Known Hh-signaling pathway target genes. **Table S2:** Result of all pairwise comparisons of differentially expressed genes. **Table S3:** Hh-signaling differentially responsive genes.
10.1186/s12867-015-0047-3 Genes regulated by either Esrrb or Hh-signaling pathway. **Figure S2:** Decision tree model of gene sorting. **Figure S3:** Hh-responsive transcription-related genes. **Figure S4:** Correlation of qPCR and RNA-seq of top 15 Hh differentially responsive genes. **Figure S5:** Esrrb-dependent Hh-responsive genes. **Figure S6:** Glucocorticoid Receptor related genes in response to Hh-CM and Esrrb.

## Abbreviations

Esrrb: Estrogen related receptor β
Hh: Hedgehog
Hh-CM: Hh ligand conditioned medium
iPSC: induced pluripotent stem cells
OSKM: Oct4, Sox2, Klf4, cMyc
FBS: fetal bovine serum
RT: reverse transcriptase
KEGG: kyoto encyclopedia of genes and genomes
GO: gene ontology
FC: fold change
