# Supplementary material for: Genes targeted by the Hedgehog-signaling pathway can be regulated by Estrogen related receptor β
Source: BMC Mol Biol. 2015 Nov 23;16:19. doi: 10.1186/s12867-015-0047-3 (PMC4657266; doi:10.1186/s12867-015-0047-3)
Supplement: Supplementary file 1 — 10.1186/s12867-015-0047-3 Known Hh-signaling pathway target genes. Table S2: Result of all pairwise comparisons of differentially expressed genes. Table S3: Hh-signaling differentially responsive genes. [file 12867_2015_47_MOESM1_ESM.pdf]

Supplement Table 1. Known Hh-signaling pathway target genes

Reference Title	Experiment Method	Upregulated genes(Log2 Fold change >1)	Downregulated genes (Log2 Fold change <-1)
Sonic Hedgehog-responsive Genes in the Fetal Prostate	Species: mouse<br>Tissue type: UGS cells<br>Method: Microarray<br>Treatment: Hh peptide	Brak<br>Dner<br>Gli1<br>Fgf5<br>Ptch1<br>Rasl11b<br>Map3k12<br>Cd24a<br>Timp3<br>Cldn2<br>Ccdc3<br>angpt4<br>hsd11b1<br>camk1d<br>tnmd<br>rrm2b<br>smoc2<br>vldlr<br>lgi2<br>5133400d11rik<br>foxd1<br>cdc14a<br>al024069<br>slc6a6<br>stxbp6<br>artn<br>ksr<br>steap4<br>tmem69<br>rbpms<br>serpinb9b<br>1700080016rik<br>fbn2<br>id4<br>igfbp3<br>pde9a<br>trib2<br>prdm8<br>gpr73<br>efnb2<br>ntrk3<br>scara5<br>4933425f03rik<br>fblim1<br>slitrk6<br>uchl3<br>tiam1<br>igfbp6<br>slc7a12<br>fa2h<br>mr1<br>plxna2<br>plod2<br>hes1	Dmp1<br>mmp13<br>wdr61<br>sufu<br>fkbp1a<br>atg121<br>4930523c07rik<br>rgs4<br>cxcl14<br>loc544932<br>adam12<br>tcfcp213<br>clca1<br>clca2<br>au022121<br>cx3cl1<br>tnfaip6<br>nr1h4<br>kremen1<br>spdy1

		rbm24	
		loc380843l1l0007al3rik	
		gpt2	
		fmo1	
		spag9	
		rrm2b	
		lhfp	
		sos1	
		amisyn	
		fads2	
		scarb1	
Genome-Wide Screening Reveals an EMT Molecular Network Mediated by Sonic Hedgehog-Gli1 Signaling in Pancreatic Cancer Cells	Speccies: human<br>Tissue type: AsPC-1 cell<br>Method: Microarray<br>Treatment: Gli1 overexpression	KRT20	RPP40
		KLK7	PCBD2
		EMP1	HSD17B6
		EGR3	MTHFD2L
		DUSP5	C1orf59
		GPA33	HCG18
		UCA1	PTPN2
		IL8	OGFRL1
		CST6	GPR44
		IER3	PRICKLE1
		CAV1	AGMAT
		LGALS1	C9orf86
		TSPAN1	CFL2
		S100A4	CDK6
		NRG1	SLC16A9
		CLIC5	NPEPL1
		WNT5A	SOLH
		MALAT1	SCLY
		ANKRD22	PPIF
		MALL	MSI2
		AHNAK2	TRPS1
		RGS14	C9orf122
		ALOX5	PNO1
		EGR1	HPGD
		MIR21	SLC39A12
		ALDH3A1	MCM4
		TNFRSF12A	PIGW
		CEACAM6	STX3
		S100A6	KIT
		CADPS	SESN1
		FOS	CTPS
		IL33	SLC1A3
		BIRC3	G3BP2
		CHI3L1	STRADB
		HIST1H4C	FOLH1
		ATAD2	SRM
		ATF3	B3GALNT1
		SCARA3	SEC61A1
		CTTN	TMEM87A
		CYR61	TMEM5
		TIMP3	SKP2
		S100A2	RPL27A
		AKR1C2	PTGS2
		AKR1C3	ADK
		SDC4	CRTAM
		ARL4C	PAH
		HIVEP2	ING3
		PLAUR	HUS1
		HEPH	C1QTNF3
		KRT80	KCNJ8

ITGB6
SIX4
CEP110
KLF9
LRRC1
KTN1
TIMP1
TIMP2
C19orf33
SPRR3
SLC16A5
ARL14
SYT17
SERPINB5
KLK1
SPINK4
ITGA3
RRAS
NR1D2
TOX2
KLK10
C2orf68
TNC
TGFBR3
FAM119A
ENPP5
TGFB2
LOC100129105
NEURL1B
KLF4
KLRC1/2
ITPKB
ANG
RNASE4
NCRNA00173
DUSP6
SCD
FAM100B
PLEKHA8
JDP2
GPRC5A
GBP3
NAB2
MAFF
GSDMB
S100A14
RPL31
IER5L
FOXO1
ZMIZ1
IDS
SAT1
MYADM
RHOU
H1F0
GADD45B
ANXA6
QSOX1
ABCC3
ID3
NFAT5
DIRAS2
KBTBD8
AMACR/C1QTNF3
CHST13
TPK1
TDO2
SMOC2
KITLG
RBM8A

GDA
RASA4
MSX2
IGFBP6
PTPRF
SPRY4
UACA
HISPPD2A
SELENBP1
IGFL2
PSMB9
TNNT1
SGOL2
HES1
ZFP36
KLK11
MT1F
FOXQ1
TFPI
KLF13
CMPK1
EXT2
AHNAK
KLRC3
TM4SF1
RAB27B
KLF12
MIDN
BHLHE41
KLF6
TNFAIP8L1
METTL7A
AKR1C1
HOXC6
JHDM1D
PRAGMIN
IL17RD
S100A11
IL27RA
HOXB5
LY6G6D
IL18
MST1R
NEO1
C1orf116
KLRK1
EGFR
LFNG
BCL2
IRS1
CA12
ABCG1
TMCC3
SERTAD2
APOBEC3B
MED13L
MT1X
LAMB3
VEGFA
OVOL1
GJA3

CASC5
DBP
IQGAP3
SYTL2
GAS2L3
PDGFA
TFF1
RIN2
FAM110C
C1orf133
PTTG3
ID2
GSTA4
RPS6KA3
TBX3
C20orf112
ANKRD57
SAP30L
CSRNP1
RAB8B
CCNG2
TGOLN2
RHEBL1
DUSP4
EIF5A2
SERTAD4
TRIM15
MT1E/1H/1M
GNAL
MT2A
CLMN
OVOS/OVOS2
SOX13
PPARGC1A
TRIP10
PCDH7
ETV5
MKI67
SOX4
FAM43A
CAPRIN2
YPEL5
LIF
TMEM49
ENDOD1
CAV2
LDLR
SFXN3
ARHGAP29
MT1H
EPS8L3
IQGAP2
ATP9A
C17orf61
BCL6
CD59
SFRS18
C14orf4
MACF1
KDM5B
EPHB6

		SPRED1<br>KLF5<br>PAM<br>ID1<br>SBF2<br>ARHGAP28<br>KLK6<br>MICAL2<br>ANXA1<br>TNIK<br>ZDHHC3<br>SCG5<br>C7orf41<br>SEPP1<br>IGF1R<br>IFITM1<br>CTSB<br>PTK6<br>NUDT4<br>CD97<br>PLAU<br>POLD4<br>PPM1A<br>TNS4<br>AK7<br>PTTG1<br>HRCT1<br>SGPP2<br>PDK4<br>PTCH1<br>ZSWIM6<br>SERPINA1<br>EFHD2<br>ITGB4<br>LPHN1<br>PITPNM3<br>C5<br>UNC84B<br>FAM131B<br>MLLT6<br>FOXN3<br>CDC2L6<br>ZFHX3<br>C11orf70<br>ATP6V1C2	
Shh signaling is essential for rugae morphogenesis in mice	Species: mouse<br>Tissue type: palatal shelves of embryos dissected from pregnant mice one-day post<br>Method: Microarray<br>Treatment: Shh antibody arresting<br>Note: "upregulated genes" in this group are Shh arresting downregulated genes	sostdc1<br>ptch1<br>gli1	shh<br>axin2<br>dkk1<br>gata3
The GLI genes as the molecular switch in disrupting Hedgehog signaling in colon cancer	Species: human<br>Tissue type: HT29 cells<br>Treatment: GANT61<br>Note: "upregulated genes" in this [illegible]	TYMS<br>TK1<br>TOP2A<br>RRM1<br>RRM2<br>PRPS2	

		POLE	
		POLE2	
		POLA1	
		POLA2	
		POLQ	
		E2F2	
		CDT1	
		PRIM1	
		GMNN	
		RFC2	
		RFC3	
		RFC4	
		RFC5	
		H2AFX	
		MDC1	
		BRCA1	
		FANCD2	
		BARD1	
		CDC45L	
		DDIT2	
		DDIT3	
		DDIT4	
		PPP1R15A	
		PCNA	
		ATF3	
		RAD51	
		RAD51C	
		RAD54B	
		RAD54L	
		FEN1	
		MSH6	
		KIAA0101	
		UNG	
		LIG1	
		EXO1	
		HELLS	
Ablation of Indian Hedgehog in the Murine Uterus Results in Decreased Cell Cycle Progression, Aberrant Epidermal Growth Factor Signaling, and Increased Estrogen Signaling	Species: mouse<br>Method: Microarray<br>treatment: IHH ablation<br>Note: "upregulated genes" in this g	1110064A23Rik	1110001D15Rik
		1700010B09Rik	1190002H23Rik
		2810417H13Rik	1190002H23Rik
		4930413G21Rik	A2m
		4930413G21Rik	Abi3bp
		4930426I24Rik	Accn1
		4930556L07Rik	Adamdec1
		Acta1	Akr1c14
		Amhr2	Ano4
		Amhr2	Aox3
		Angptl7	Aqp5
		Anln	Arg2
		Asl	Arg2
		Aspm	Aspg
		Atf4	BB144871
		AU024342	BC048679
		Aurka	Bex1
		AW548124	Car3
		Birc5	Ccl28
		Bub1	Cd36
		Ccna2	Cd36
		Ccna2	Chga
		Ccnb1	Clca3
		Ccnb1	containing
		Ccnb2	Cpm

	Ccne2	Cxcl14
	Cdc20	Fos
	Cdc2a	Gdpd3
	Cdca3	Gem
	Cdca8	Gstm7
	Cdca8	Gulo
	Cdca8	H2-T23
	Cenpa	Hba-a1
	Cenpe	Hbb-b1
	Cenpf	Iapp
	Centd2	Iapp
	Cep55	Iapp
	Cks2	Inhbb
	Cks2	Inmt
	Cspg2	Krt85
	cycle	Lcn2
	cycle	Mfsd4
	Cyp1b1	Mitf
	Dleu7	Moxd1
	Dll4	Myb
	Ect2	Myo3b
	Eno4	Olfm4
	Fignl1	Oxtr
	Fst	P2ry14
	Fst	Pamr1
	Gli1	Papers
	Hrasls	Prlr
	Hsd17b3	Prlr
	Il13ra2	Rab6b
	Irg1	Rbm39
	Kcnd3	regeneration
	Kif11	Ror2
	Kif20a	Sord
	Kif23	Sox9
	Maob	Spink3
	Mcm2	Tgfbi
	Mcm3	Tgfbi
	Mcm5	Tgfbi
	Mcm5	Tgfbi
	Mcm6	Transcribed
	MGC73635	Transcribed
	Mki67	Wnt4
	Mtap2	
	Ncapd2	
	Ncapg	
	Ndp	
	Nek2	
	Nt5dc2	
	Nuf2	
	Nusap1	
	Papers	
	Papers	
	Pbk	
	Pcsk5	
	Pcsk5	
	Pkdcc	
	Plk1	
	Prc1	
	Ptch1	
	Ptch2	
	Pthr1	

		Racgap1	
		Rad51	
		Rgs2	
		Rrm2	
		Rrm2	
		Shcbp1	
		Slc6a2	
		Smc4	
		Spc25	
		Steap4	
		Stmn1	
		Stmn1	
		Tac2	
		Tacc3	
		Tcf19	
		Thy1	
		Tk1	
		Tmem132c	
		Top2a	
		Tpx2	
		Trip13	
		Tssk3	
		Ube2c	
		Uhrf1	
		Unknown	
		Vcan	
		Vgll3	
		Vgll3	
		Vit	
		Wfdc1	
GLI2 Knockdown Using an Antisense Oligonucleotide Induces Apoptosis and Chemosensitizes Cells to Paclitaxel in Androgen-Independent Prostate Cancer	Species: human<br>Tissue type: PC3 cells<br>Method: Microarray<br>treatment: Gli2 knock down<br>Note: "upregulated genes" in this group are Gli2 knock down downregulated genes	ACOT7	BMF
		ANO10	BMF
		APBA3	C9orf72
		BCL2L1	C9orf72
		BCL2L1	CCDC17
		CASC4	CCDC17
		CASC4	CDKN1A
		CCNY	CDKN1A
		CCNY	CTIF
		CMTM4	CXCL13
		COG5	CYLC2
		DDR2	DDIT3; FUS
		ENOSF1	DEGS1
		ENOSF1	FLJ44342
		EXOC4	FLJ44342
		FAM155A	GDF15
		FAM168A	GEM
		FAM20C	HOXD3
		FMNL3	HPN
		FMNL3	IFITM1
		FNDC3B	IL24
		FNDC3B	KRT82
		GALNT18	LINC01094
		HOXB13	LINC01094
		IPO8	LOC100132319
		ITFG3	LOC101929239
		KCNMA1	LOC101929239
		KHK	NR3C1
		KHK	PKD2L2
		LMO2	PLSCR1
		MSI2	PMAIP1

		MYLK	PPP1R15A
		MYLK	PTPRG
		NPAS2	PYGM
		NPR3	RSAD2
		NPR3	SPESP1
		NRG2	SPRR1A
		PALD1	STC2
		PPFIA1	TDRD7
		PPFIA1	TDRD7
		PREX1	TMOD4
		PRR16	TMOD4
		RNFT2	YOD1
		RUNDC1	ZCCHC8
		RUNDC1	
		SEMA4F	
		SIPA1L3	
		SLITRK3	
		SLITRK3	
		SORL1	
		SUPT3H	
		SUPT3H	
		TBC1D19	
		WDR70	
		WDR70	
		WDR76	
		ZNF124	
		ZNF185	
		ZNF185	
Hedgehog inhibition prolongs survival in a genetically engineered mouse model of pancreatic cancer	Species: human<br>Tissue type: HPNE-Gli1(IC3B10)<br>Method: Microarray<br>Treatment: Gli1 overexpression	C3orf14	DHDH
		HEPH	NUFIP1
		ENST00000375284	TNC
		AGT	FTSJ3
		AGT	SLC2A9
		AGT	PRKCBP1
		AGT	C20orf42
		AGT	SETD6
		AGT	AK092668
		RGC32	EXOSC4
		AGT	NOLC1
		SERTAD4	GLDN
		CDKN1C	DMKN
		AGT	AF247042
		LYPD1	LIMS3
		ENST00000357303	SCUBE2
		AK125162	THC2284350
		TRPA1	A_24_P587621
		AGT	PAGE1
		AGT	ENST00000369797
		BX248748	MGC4294
		RORB	POLR1B
		THC2330271	ACCN2
		ENST00000371528	TTC29
		NPFFR2	PITPNC1
		SLC7A14	PLA2G3
		FLJ37228	THC2263651
		ENST00000367013	ENST00000356931
		NPFFR2	PDSS1
		ENST00000366569	FMOD
		ATP1A2	KCNJ15
		ACTG2	GIMAP5
		LYPD1	IPO4

DENND2A	C22orf35
ADRA1B	LYN
WFDC1	EPB41L1
RGC32	ODZ4
MFAP4	HHIP
ENST00000367012	TMOD1
ANXA3	LYN
SIRPA	DPH2
RP1-93H18.5	F2RL2
ADARB1	HK2
BHMT2	AA714039
CR605298	LYN
ZNF610	A_23_P170719
TRAM1L1	LYN
ENST00000371256	LOC92312
SAA1	VSNL1
SDS	DCBLD2
HTRA3	PRSS3
SELENBP1	LYN
SLC7A14	TNNI3
CCRK	AK024680
ENST00000366930	C9orf58
DENND2A	LYN
MAGEA2B	KRTAP1-5
NPY2R	C18orf50
TSPY2	WDR3
LZTS1	METTL7B
DMGDH	WDR3
THC2314822	LOC441461
EREG	LYN
CNN1	HAVCR1
EPHB4	ANGPTL4
AL832540	LYN
GSTM3	THC2283371
BOK	AF116675
SAA2	LYN
LAMA5	LRRC17
SLITRK6	EPB41L1
NRXN1	IL12RB2
AI470277	LYN
BHMT2	ENST00000339446
ENST00000321925	COLEC11
C10orf10	BC038983
DMN	SMOC1
TMEM130	ZNF334
TNFRSF11B	SERPINB2
TP53I11	AKR1B10
GSTM3	A_23_P111766
CSTA	RPL22L1
DPP4	HSPC111
W04231	FOLR3
TP53I11	PEO1
EDN1	EDG8
EDN1	EDG8
FLJ34515	SLC6A15
EDN1	KIAA0690
EDN1	PAPPA2
CKB	C21orf34
THBS1	THC2274524
EDN1	LAMA1
XAGE2	COL14A1

THBS1	PLAC8
EDN1	POLR1B
EDN1	LOC391403
EDN1	OXGR1
ECHDC2	BC018675
EDN1	DPYSL4
THBS1	RDM1
AK001966	CTSH
TIAM2	SPHK1
EDN1	ZNF179
THC2435731	PEO1
KIF26B	PTPRE
THBS1	DCBLD2
FOXF1	IL22RA1
ST6GAL1	AKAP12
THBS1	TMEM46
CSTA	THC2279790
THBS1	LOC401131
THBS1	THC2379144
THBS1	KBTBD11
THBS1	PRSS2
MAGEA4	ENST00000383097
MAGEA6	NIPSNAP1
THBS1	A_32_P167212
CES1	PTPN22
FLJ14213	SLC35F2
MAGEA6	BM129308
BE786351	BC033829
PLEKHA6	CPEB1
CLEC3B	AKAP12
MAGEA6	IFNE1
LOC387895	IL23R
BX373237	LPHN3
COL11A1	ZDHHC11
CHRM3	ARG2
D4S234E	CFI
TNFRSF11B	DACH2
MAGEA6	HLA-DOA
GALNTL2	ENST00000267857
MAGEA6	MGC45438
CXCL11	DACH2
ENST00000343905	CMYA3
MAGEA6	LOC400890
MAGEA6	RPS6KA5
AK001007	LOC92312
TAGLN	PRSS2
LOC147710	ACP5
TGFB2	HLA-DRB5
MAGEA6	PECAM1
MAGEA6	HLA-DPA1
F3	ZNF659
AL355687	CR936711
OLFML2A	HLA-DPA1
GABBR2	MYOM2
AMZ1	IFI44L
SPON2	LAMA1
PLAU	DPPA2
PLAU	RSAD2
KCNIP3	ENST00000272643
ESR2	GPR103
PLAU	M27126

ESAM	IMPA2
BC062758	PTPRE
TNFRSF11B	KHK
C18orf2	DHRS9
MYOZ2	FAM5C
ENST00000382416	RSAD2
CXCL11	ENST00000367169
PLAU	PODXL
PLAU	RP11-393H10.2
TNFRSF11B	PXDNL
THC2312468	HLA-DRB4
PLAU	MFAP5
THBS1	ACTR3B
BEXL1	UGT2B11
PLAU	FLJ20581
MAGEA6	AX721193
PLAU	ANXA10
TNFRSF11B	STK33
PLAU	THC2374814
CXCL10	CFI
AV707592	SYT13
PLAU	GPR30
ENST00000376752	SLAMF1
ChGn	FAM78A
ALDOC	SLAMF7
TNFRSF11B	SEPP1
BF213738	TMPRSS11E
SKIP	BC043411
KENAE	THC2440554
TNFRSF11B	C6orf114
AW949170	SMOC1
ADAM8	SLC26A4
THC2314823	DOCK8
SNED1	AK125648
C18orf2	LOC400879
TNFRSF11B	SPTA1
THC2314600	SV2B
PPP1R1C	DA711948
THC2403712	NFIB
UNC5B	CITED4
BX538051	SOHLH2
A_24_P917668	GALNT12
GREM2	ENST00000257897
DACT1	GREB1
EIF4E3	ZNF447
TNFRSF11B	NOSTRIN
THC2354272	ESM1
TNFRSF11B	CREB5
AF086044	ENST00000381655
TRPA1	SULF1
MAGEA12	AK126405
VMD2	INPP5D
C10orf10	ARRB1
CYB5R2	LGALS9
KCTD14	C9orf58
ITGB8	FLJ10324
TNFRSF11B	DPT
ETS2	PLP1
SVEP1	C20orf133
CYP4V2	SERPING1
C10orf125	IL13RA2

FOXA1	RP11-119E20.1
FLJ14213	CD53
RASL11B	LOC284998
NEDD9	A_23_P72014
ENST00000380186	IL27RA
RHOJ	PLEKHA7
SNTA1	OCIAD2
XAGE1	RHBDF2
CR740121	CGNL1
CRISPLD2	FAM105A
PTER	BG118529
ESR2	QPRT
BX117479	NAP1L3
RAB11FIP1	C20orf133
39329	C10orf58
GALNTL1	QPRT
SYNC1	PCSK9
MEST	UGT2B7
ESR2	ALDH1A1
KCNJ6	EGFL11
GRIA3	MMP1
TNIP3	FBLN2
PPARGC1A	MMP1
GBP2	MMP1
DLL3	MMP1
DDX43	MMP1
MGC16121	RP1-32F7.2
C10orf125	MMP1
C20orf132	THC2375723
THC2439128	MMP1
REPS2	MMP1
EMID1	MMP1
CRISPLD2	DENND2D
GRAMD1C	ICAM2
CEBPD	THC2355280
FAM49A	MMP1
CSAG3A	BG114486
SNTA1	GCNT3
RAB11FIP4	TMEM16D
ADORA2B	AK058065
C1orf198	FLJ30851
AF035291	DSC3
SIRPG	HLA-DMB
TGM2	IRX3
TCEA3	SPP1
BAALC	SPP1
ANGPT1	DSC3
CYGB	SPP1
SYNPO2	SPP1
FLJ46385	SPP1
C1S	HLA-DRA
BDKRB2	SPP1
WISP2	THC2455389
RHOJ	A_24_P561165
OXTR	AK093002
SORT1	SPP1
TPM1	SPP1
AK027294	SPP1
GLI1	SPP1
KIAA0367	TNFSF7
RNF157	RYR2

	FMR1NB	FLJ30851
	TCEA3	ENST00000295989
	TPM1	RP1-32F7.2
	ARHGAP6	ECHDC3
	RASSF4	BCAN
	PCSK5	GIMAP2
	ANGPT1	BCL2A1
	EDG1	MAGEC2
	AK094972	DYDC2
	C6orf194	ATP8A2
	ZNF415	PREX1
	STAT5A	TIE1
	TPM1	DOCK8
	THC2285198	TMEM16D
	NNMT	DOCK8
	TUBA1	
	ADM	
	ADM	
	MYLK	
	SVEP1	
	LCE3A	
	ADM	
	METTL7A	
	OPLAH	
	C5orf23	
	C13orf16	
	SORT1	
	WDR66	
	C20orf132	
	ADM	
	GLUL	
	FOXO1A	
	PLCL1	
	CD633154	
	C20orf175	
	ADM	
	DDIT4	
	DPP7	
	ADM	
	ADM	
	SEMA3B	
	ADM	
	ADM	
	ESR2	
	BC089451	
	AA910154	
	TAGLN	
	ChGn	
	ADM	
	THC2442494	
	PALLD	
	BIRC3	
	CLDN1	
	C21orf7	
	A_24_P398370	
	METTL7A	
	RORB	
	BMP2	
	SIRPA	
	BDNF	
	GPR1	

	KIT
	PDE5A
	EDIL3
	FLJ22536
	ZNF594
	PIM1
	B3GALT4
	RHOJ
	BDNF
	EREG
	ETS2
	LOC340061
	RNF128
	MASP1
	ARHGEF5
	AK123483
	ZNF287
	B3GALT4
	C1orf38
	IGF2
	LBH
	C4orf18
	CD9
	LHX6
	RAB4A
	PPAP2B
	DSCR1L1
	DOC1
	CRIM1
	A_32_P327750
	THC2364378
	PLK2
	SVEP1
	ENST00000334564
	WISP1
	PCDH18
	LOC144501
	HERC5
	CLEC2B
	CYP27A1
	COL5A1
	A_24_P212997
	MUC15
	CD40
	SPECC1
	GRAMD1C
	TXNIP
	THC2411757
	RBMY1B
	ENST00000372234
	FLJ43080
	CYR61
	ABCA13
	IQCG
	CCDC68
	TCEA3
	ESR2
	TPM1
	SPAG8
	AK094154
	ENPP5

		FLJ21075	
		ABCC3	
		BQ379494	
		CALCR	
		KLF9	
		ATF3	
		USP48	
		RBMY1B	
		USP48	
		ENST00000360934	
		MAP4	
		RGS9	
		ECGF1	
		LOC201229	
		MYLK	
		MYH10	
		SLC38A4	
		SOCS2	
		EFNB2	
		TMEM35	
		STC2	
		GARNL4	
		A_32_P188127	
		AA292106	
		THC2286259	
		FOXA1	
		LOC652408	
		C11orf70	
		CD40	
		HEPH	
		CD40	
		TP53I11	
		ENST00000380064	
		NRIP3	
		JUB	
		FAM107B	
		TPST1	
		USP48	
		CHRM2	
Molecular Pathways Regulating Pro-migratory Effects of Hedgehog Signaling	Species: mouse	*Gli*	*Cxcl5*
	Tissue type: C166 cells	*Ptch1*	*Rgs4*
	Method: microarray	*MICAL2*	*Atp1b1*
	Treatment: Hh lignad enriched conditioned medium	*Nrp*	*Pdk4*
		Klf4	*Ccl7*
		Igfbp3	*Sphk1*
		Slc6a6	*Emb*
		Capn1	*Mep1a*
		Tns	*Nelf*
		Penk1	*Pvt1*
		Tbx18	*Arhu*
		Hspa1b	*L1cam*
			Thbs2
Lubahn lab unpublished dataset	Species: mouse	Itga6	Cpne6
	Tissue type: TRAMPC2 cells	Sorcs2	Pamr1
	Treatment: Hh lignad enriched co	Cp	Mmp13
	Method: RNAseq	Synm	Gda
		Foxo6	Sprr1a
		Mtss1	Smtnl2
		Cp	Slc10a6

	Fzd4	Mt2
	Mycl1	Sncaip
	Sykb	Fos
	Lrrc32	Fndc1
	Epb4.1l4b	Rgs4
	Grb14	Agap2
	Scara5	Mmp3
	Nsg2	Kbtbd11
	Srpx2	Il1rn
	Cyb561	Stc1
	Gas7	Ppl
	Kif26b	Mt1
	Plxna2	Gatm
	Hs3st3b1	
	Cd24a	
	Map3k5	
	Ablim1	
	Ngef	
	Abca1	
	Paqr8	
	Slco4a1	
	Itga9	
	Pkdcc	
	Nhs	
	Fbxo32	
	Pid1	
	Rbm20	
	Gdpd5	
	Angpt4	
	Enc1	
	Rasl11b	
	Ccdc3	
	Syt13	
	Alpl	
	Fbn2	
	Ace	
	Hes1	
	Perp	
	Bmper	
	Smoc2	
	Enpp1	
	Trp63	
	Foxd1	
	Bcl11b	
	Atp6v0a4	
	Lama5	
	Penk	
	Col15a1	
	Ptch1	
	Smoc1	
	Cldn9	
	Col23a1	
	Igfbp5	
	Kcnd3	
	Tgm5	
	1810041L15Rik	
	Pla2g3	
	Ddit4l	
	Bdkrb2	
	Hsd11b1	
	Ptch2	

	Gli1
	Cacna2d2

Supplment2 Result of all pairwise comparisons of differentially expressed genes

	Control vs Hedgehog			
gene	log2(fold_change)	p_value	q_value	Gene Symbol
NM_030206	inf	0.00005	0.00089	Cygb
NM_007902	inf	0.00005	0.00089	Edn2
NM_010296	9.14	0.00020	0.00307	Gli1
NM_001044751	4.40	0.00005	0.00089	Hsd11b1
NM_008623	3.92	0.00230	0.02481	Mpz
NM_008957	3.82	0.00005	0.00089	Ptch1
NM_013690	3.81	0.00005	0.00089	Tek
NM_008235	3.73	0.00005	0.00089	Hes1
NM_001271705	3.70	0.00005	0.00089	Pgf
NM_016867	3.47	0.00005	0.00089	Gipc2
NM_001099276	3.23	0.00005	0.00089	Pik3c2b
NM_177709	3.15	0.00255	0.02711	Tusc5
NM_025429	3.04	0.00005	0.00089	Serpinb1a
NM_001177713	2.97	0.00005	0.00089	Cyp26b1
NM_009117	2.92	0.00440	0.04223	Saa1
NM_009641	2.82	0.00005	0.00089	Angpt4
NM_007719	2.81	0.00005	0.00089	Ccr7
NM_016808	2.79	0.00005	0.00089	Usp2
NM_009115	2.78	0.00040	0.00567	S100b
NM_010518	2.65	0.00005	0.00089	Igfbp5
NM_009437	2.64	0.00005	0.00089	Tst
NM_001281819	2.61	0.00005	0.00089	Ace
NM_008380	2.57	0.00005	0.00089	Inhba
NM_022657	2.56	0.00005	0.00089	Fgf23
NM_001033633	2.46	0.00005	0.00089	Slc2a13
NM_001012766	2.45	0.00005	0.00089	Ear12
NM_010740	2.38	0.00005	0.00089	Cd93
NM_001177752	2.36	0.00005	0.00089	Pfkfb3
NM_001285457	2.35	0.00005	0.00089	Gfra1
NM_139197	2.34	0.00005	0.00089	Gbgt1
NR_030721	2.31	0.00005	0.00089	Foxd2os
NM_145356	2.26	0.00005	0.00089	Zbtb7c
NM_008489	2.25	0.00005	0.00089	Lbp
NM_001170847	2.25	0.00005	0.00089	Rbm20
NM_011452	2.21	0.00005	0.00089	Serpinb9b
NM_009382	2.16	0.00005	0.00089	Thy1
NM_172671	2.15	0.00005	0.00089	Lgr4
NM_023734	2.14	0.00030	0.00442	Pi16
NM_019444	2.12	0.00005	0.00089	Ramp2
NM_001167680	2.11	0.00005	0.00089	Rhbdf2
NM_016854	2.09	0.00005	0.00089	Ppp1r3c
NM_001142952	2.07	0.00005	0.00089	Fam46c
NM_001164355	2.06	0.00015	0.00240	Ska1
NM_001145886	2.03	0.00005	0.00089	Tiam1
NM_007539	1.95	0.00005	0.00089	Bdkrb1
NM_153137	1.95	0.00010	0.00167	Traf3ip3
NM_001159518	1.91	0.00005	0.00089	Igfbp7
NM_001033141	1.88	0.00060	0.00814	Ecscr
NM_177343	1.87	0.00005	0.00089	Camk1d
NM_001127259	1.87	0.00005	0.00089	Trp63
NM_001168318	1.86	0.00005	0.00089	Scara5
NM_008737	1.85	0.00005	0.00089	Nrp1
NM_009548	1.84	0.00020	0.00307	Rnf112
NM_001195025	1.81	0.00005	0.00089	Nuak2
NM_007470	1.80	0.00005	0.00089	Apod
NM_018827	1.79	0.00005	0.00089	Crlf1
NM_031168	1.79	0.00005	0.00089	Il6

NM_146149	1.77	0.00010	0.00167	Fam151a
NM_010517	1.76	0.00005	0.00089	Igfbp4
NM_018779	1.75	0.00005	0.00089	Pde3a
NM_001033455	1.75	0.00005	0.00089	Ccdc27
NM_009931	1.73	0.00005	0.00089	Col4a1
NM_026778	1.72	0.00005	0.00089	Cthrc1
NM_172676	1.71	0.00005	0.00089	Samd10
NM_008593	1.71	0.00205	0.02240	Foxd2
NM_030172	1.71	0.00090	0.01150	Efcab11
NM_001033464	1.68	0.00005	0.00089	Efcab4b
NM_019971	1.68	0.00005	0.00089	Pdgfc
NM_001199696	1.68	0.00005	0.00089	Bai2
NM_009860	1.66	0.00005	0.00089	Cdc25c
NM_008185	1.63	0.00005	0.00089	Gstt1
NM_181848	1.63	0.00005	0.00089	Optn
NM_016851	1.62	0.00005	0.00089	Irf6
NR_015556	1.59	0.00005	0.00089	2610035D17Rik
NM_001081085	1.58	0.00005	0.00089	Sapcd2
NM_025590	1.58	0.00005	0.00089	Acot11
NM_027930	1.58	0.00005	0.00089	Mtfr2
NM_011623	1.56	0.00005	0.00089	Top2a
NM_008055	1.56	0.00005	0.00089	Fzd4
NR_015456	1.54	0.00450	0.04299	D7Ertd715e
NM_007805	1.53	0.00005	0.00089	Cyb561
NM_134072	1.51	0.00005	0.00089	Akr1c14
NM_001162506	1.51	0.00005	0.00089	Troap
NM_172564	1.51	0.00005	0.00089	Tns4
NM_053191	1.50	0.00005	0.00089	Pi15
NR_033225	1.50	0.00005	0.00089	Gm13375
NM_172418	1.50	0.00005	0.00089	Mamstr
NM_013822	1.50	0.00005	0.00089	Jag1
NM_198423	1.49	0.00005	0.00089	Bahcc1
NM_033041	1.49	0.00175	0.01972	Hes7
NM_001081258	1.49	0.00005	0.00089	Kif14
NM_001163471	1.48	0.00005	0.00089	Hectd2
NM_001111274	1.47	0.00005	0.00089	Igf1
NM_001081363	1.47	0.00005	0.00089	Cenpf
NM_001110265	1.47	0.00005	0.00089	Ttk
NM_007695	1.46	0.00005	0.00089	Chil1
NM_001164724	1.45	0.00005	0.00089	Il33
NM_001113460	1.45	0.00005	0.00089	Tec
NM_001081117	1.44	0.00005	0.00089	Mki67
NM_146208	1.44	0.00005	0.00089	Neil3
NM_009932	1.44	0.00005	0.00089	Col4a2
NM_144799	1.43	0.00025	0.00374	Lmcd1
NM_009252	1.42	0.00005	0.00089	Serpina3n
NM_001252055	1.42	0.00005	0.00089	Ly6c1
NM_029617	1.40	0.00005	0.00089	Casc5
NM_013538	1.39	0.00005	0.00089	Cdca3
NM_009320	1.39	0.00005	0.00089	Slc6a6
NM_001111073	1.38	0.00005	0.00089	Fxyd5
NM_001081125	1.37	0.00005	0.00089	Gli2
NM_001177794	1.37	0.00005	0.00089	Sertad4
NM_027975	1.37	0.00005	0.00089	Fam83d
NM_001081020	1.37	0.00005	0.00089	Adamts6
NM_007993	1.36	0.00005	0.00089	Fbn1
NM_019753	1.36	0.00005	0.00089	Cdh17
NM_001110162	1.35	0.00005	0.00089	Cdca2
NM_010637	1.35	0.00005	0.00089	Klf4
NM_028481	1.35	0.00015	0.00240	Ccdc18
NM_008209	1.34	0.00005	0.00089	Mr1

NM_001272057	1.34	0.00005	0.00089	Adam5
NM_001085549	1.34	0.00005	0.00089	Trabd2b
NM_176982	1.34	0.00015	0.00240	Fbxo48
NM_009013	1.33	0.00005	0.00089	Rad51ap1
NR_001592	1.33	0.00005	0.00089	H19
NM_028870	1.31	0.00005	0.00089	Cltb
NM_133687	1.31	0.00005	0.00089	Cxxc5
NM_008796	1.31	0.00090	0.01150	Pctp
NM_173762	1.30	0.00005	0.00089	Cenpe
NM_001032413	1.29	0.00005	0.00089	Pear1
NM_028705	1.29	0.00005	0.00089	Herc3
NM_080467	1.28	0.00005	0.00089	Atp6v0a4
NM_134000	1.27	0.00155	0.01783	Traf3ip2
NM_013807	1.27	0.00005	0.00089	Plk3
NM_001042421	1.27	0.00005	0.00089	Kntc1
NM_026743	1.26	0.00005	0.00089	Tspan11
NM_009791	1.26	0.00005	0.00089	Aspm
NM_026613	1.26	0.00005	0.00089	Ccdc34
NM_008458	1.25	0.00080	0.01038	Serpina3c
NM_013552	1.25	0.00005	0.00089	Hmmr
NM_172145	1.25	0.00005	0.00089	Eva1b
NM_028829	1.25	0.00005	0.00089	Paqr8
NM_001159369	1.25	0.00005	0.00089	Polq
NM_134117	1.25	0.00005	0.00089	Pkdcc
NM_010657	1.24	0.00005	0.00089	Hivep3
NM_178804	1.24	0.00005	0.00089	Slit2
NM_009253	1.23	0.00005	0.00089	Serpina3m
NM_197959	1.23	0.00005	0.00089	Kif18b
NM_001164557	1.23	0.00005	0.00089	Pdzk1ip1
NM_053072	1.22	0.00005	0.00089	Fgd6
NM_010620	1.22	0.00005	0.00089	Kif15
NM_181589	1.22	0.00005	0.00089	Ckap2l
NM_001193305	1.22	0.00005	0.00089	Mical2
NM_001001334	1.21	0.00460	0.04371	BC061194
NM_001013377	1.21	0.00380	0.03758	Arhgef39
NM_054098	1.20	0.00005	0.00089	Steap4
NM_007681	1.20	0.00005	0.00089	Cenpa
NM_009378	1.19	0.00005	0.00089	Thbd
NM_010658	1.19	0.00340	0.03441	Mafb
NM_013571	1.19	0.00005	0.00089	Ksr1
NM_001177881	1.19	0.00005	0.00089	Mfap3l
NM_007925	1.19	0.00225	0.02435	Eln
NM_026410	1.18	0.00005	0.00089	Cdca5
NM_016719	1.17	0.00005	0.00089	Grb14
NM_009373	1.17	0.00005	0.00089	Tgm2
NM_008276	1.16	0.00005	0.00089	Hoxd8
NM_007696	1.15	0.00385	0.03790	Ovgp1
NM_175563	1.15	0.00005	0.00089	Prr11
NM_026507	1.15	0.00005	0.00089	Zwilch
NM_001081364	1.15	0.00005	0.00089	Arhgap21
NM_011132	1.15	0.00005	0.00089	Pole
NM_001040631	1.14	0.00005	0.00089	C1qtnf5
NM_178421	1.14	0.00020	0.00307	Nanos1
NM_198605	1.13	0.00005	0.00089	Ska3
NM_001013368	1.13	0.00005	0.00089	E2f8
NM_001172092	1.13	0.00005	0.00089	Depdc1a
NM_010892	1.13	0.00005	0.00089	Nek2
NM_153574	1.13	0.00005	0.00089	Fam13a
NM_001005341	1.12	0.00005	0.00089	Ypel2
NM_029835	1.12	0.00085	0.01093	Ticrr
NM_001012273	1.12	0.00005	0.00089	Birc5

NM_023294	1.12	0.00005	0.00089	Ndc80
NM_028039	1.11	0.00005	0.00089	Esco2
NM_172756	1.11	0.00030	0.00442	Ankle1
NM_028666	1.11	0.00005	0.00089	Fam110a
NM_025566	1.11	0.00035	0.00506	Tnfaip8l1
NM_019483	1.11	0.00020	0.00307	Smad9
NM_146235	1.10	0.00005	0.00089	Ercc6l
NM_019521	1.10	0.00005	0.00089	Gas6
NM_001040111	1.10	0.00005	0.00089	Arap1
NM_021492	1.10	0.00010	0.00167	Ap3b2
NM_008021	1.09	0.00005	0.00089	Foxm1
NR_024720	1.09	0.00070	0.00928	2700099C18Rik
NM_011264	1.09	0.00005	0.00089	Rev3l
NM_013691	1.09	0.00005	0.00089	Thbs3
NM_001081006	1.09	0.00005	0.00089	Etl4
NR_029439	1.09	0.00005	0.00089	1700018A04Rik
NM_001110504	1.08	0.00005	0.00089	Capn1
NM_178609	1.08	0.00005	0.00089	E2f7
NM_172563	1.08	0.00005	0.00089	Hlf
NM_134041	1.08	0.00005	0.00089	4930427A07Rik
NM_010468	1.08	0.00325	0.03313	Hoxd3
NM_027290	1.08	0.00005	0.00089	Mcm10
NM_001102468	1.08	0.00275	0.02878	Calml4
NM_001199123	1.07	0.00005	0.00089	Spc25
NM_001033217	1.07	0.00005	0.00089	Prickle1
NM_001163256	1.07	0.00005	0.00089	Fblim1
NM_138595	1.07	0.00275	0.02878	Gldc
NM_010615	1.07	0.00005	0.00089	Kif11
NM_139001	1.06	0.00005	0.00089	Cspg4
NR_030700	1.06	0.00175	0.01972	4831440E17Rik
NM_001162533	1.06	0.00410	0.03983	Sh3d21
NM_010110	1.06	0.00005	0.00089	Efnb1
NM_029568	1.06	0.00005	0.00089	Mfap4
NM_028266	1.05	0.00005	0.00089	Col16a1
NM_001081429	1.05	0.00045	0.00628	Ccdc15
NM_001145827	1.05	0.00005	0.00089	Stk40
NM_153762	1.05	0.00190	0.02120	Rnf26
NM_010790	1.05	0.00005	0.00089	Melk
NM_175337	1.05	0.00005	0.00089	Mlh3
NM_009764	1.05	0.00005	0.00089	Brca1
NM_011121	1.05	0.00005	0.00089	Plk1
NM_016917	1.04	0.00005	0.00089	Slc40a1
NM_001029850	1.04	0.00005	0.00089	Magi1
NM_001271729	1.04	0.00005	0.00089	Tk1
NM_013529	1.04	0.00005	0.00089	Gfpt2
NM_001284380	1.03	0.00385	0.03790	Adra1b
NM_023284	1.03	0.00005	0.00089	Nuf2
NM_026412	1.03	0.00005	0.00089	Knstrn
NM_009131	1.03	0.00010	0.00167	Clec11a
NM_001113179	1.03	0.00005	0.00089	Bub1
NM_026560	1.02	0.00005	0.00089	Cdca8
NM_001163480	1.02	0.00005	0.00089	Neurl1a
NM_144553	1.02	0.00005	0.00089	Dlgap5
NM_144526	1.01	0.00005	0.00089	Fam64a
NM_001163495	1.01	0.00005	0.00089	Arhgap19
NM_181815	1.01	0.00015	0.00240	Cep128
NM_001164362	1.01	0.00005	0.00089	Cep55
NM_016782	1.01	0.00210	0.02283	Cntnap1
NM_008344	1.01	0.00005	0.00089	Igfbp6
NM_001136071	1.01	0.00005	0.00089	Lsp1
NM_139303	1.01	0.00005	0.00089	Kif18a

NM_001177625	1.00	0.00005	0.00089	Ect2
NM_029249	1.00	0.00005	0.00089	Parpbp
NM_026878	1.00	0.00005	0.00089	Rasl11b
NM_019641	1.00	0.00005	0.00089	Stmn1
NM_153778	1.00	0.00005	0.00089	Atoh8
NM_029352	-1.00	0.00205	0.02240	Dusp9
NM_001081307	-1.02	0.00005	0.00089	Ppp1r12b
NM_008750	-1.02	0.00005	0.00089	Nxn
NM_001204201	-1.02	0.00005	0.00089	Spp1
NM_009721	-1.02	0.00005	0.00089	Atp1b1
NM_011491	-1.02	0.00005	0.00089	Stc2
NM_011610	-1.03	0.00005	0.00089	Tnfrsf1b
NM_011077	-1.03	0.00005	0.00089	Phex
NM_010516	-1.03	0.00005	0.00089	Cyr61
NM_001204129	-1.04	0.00005	0.00089	C1qtnf1
NM_011352	-1.04	0.00230	0.02481	Sema7a
NM_011723	-1.04	0.00005	0.00089	Xdh
NM_173008	-1.04	0.00005	0.00089	Ssc5d
NR_027819	-1.05	0.00005	0.00089	1810032008Rik
NM_201367	-1.05	0.00005	0.00089	Gpr176
NM_144559	-1.05	0.00475	0.04483	Fcgr4
NM_001111021	-1.05	0.00005	0.00089	Runx1
NM_053109	-1.05	0.00005	0.00089	Clec2d
NM_023785	-1.06	0.00415	0.04029	Ppbp
NM_009144	-1.06	0.00005	0.00089	Sfrp2
NM_028474	-1.06	0.00005	0.00089	Ptchd4
NM_024406	-1.07	0.00015	0.00240	Fabp4
NR_033533	-1.07	0.00050	0.00690	Gm12603
NM_007400	-1.08	0.00005	0.00089	Adam12
NM_010924	-1.08	0.00005	0.00089	Nnmt
NM_001039056	-1.08	0.00005	0.00089	Kcnj15
NM_019564	-1.08	0.00355	0.03561	Htra1
NM_018781	-1.09	0.00005	0.00089	Egr3
NM_001276489	-1.11	0.00005	0.00089	Ism1
NM_008086	-1.11	0.00005	0.00089	Gas1
NM_001081756	-1.11	0.00005	0.00089	Nckap5
NM_178738	-1.11	0.00015	0.00240	Prss35
NM_013743	-1.12	0.00005	0.00089	Pdk4
NM_023048	-1.12	0.00005	0.00089	Asb4
NM_001284507	-1.15	0.00005	0.00089	Crabp1
NR_040287	-1.16	0.00395	0.03876	A730020E08Rik
NM_009676	-1.16	0.00005	0.00089	Aox1
NM_007413	-1.17	0.00005	0.00089	Adora2b
NM_008630	-1.17	0.00005	0.00089	Mt2
NM_013495	-1.18	0.00005	0.00089	Cpt1a
NM_013468	-1.19	0.00005	0.00089	Ankrd1
NM_001029836	-1.21	0.00005	0.00089	Npnt
NM_008760	-1.21	0.00010	0.00167	Ogn
NM_025427	-1.23	0.00005	0.00089	Rgcc
NM_008176	-1.23	0.00005	0.00089	Cxcl1
NM_001039239	-1.23	0.00005	0.00089	Zfp808
NM_007420	-1.25	0.00005	0.00089	Adrb2
NM_008102	-1.26	0.00090	0.01150	Gch1
NM_013654	-1.26	0.00005	0.00089	Ccl7
NM_008491	-1.28	0.00005	0.00089	Lcn2
NM_001190911	-1.28	0.00005	0.00089	Kirrel3
NM_008695	-1.30	0.00005	0.00089	Nid2
NM_001038839	-1.30	0.00005	0.00089	P2rx7
NR_026733	-1.31	0.00005	0.00089	3110039M20Rik
NM_029600	-1.33	0.00035	0.00506	Abcc3
NM_008404	-1.35	0.00005	0.00089	Itgb2

NM_139300	-1.35	0.00005	0.00089	Mylk
NM_016689	-1.40	0.00020	0.00307	Aqp3
NM_026821	-1.41	0.00005	0.00089	Lurap1l
NM_010228	-1.42	0.00005	0.00089	Flt1
NM_013467	-1.46	0.00005	0.00089	Aldh1a1
NM_176933	-1.46	0.00005	0.00089	Dusp4
NM_001081127	-1.47	0.00005	0.00089	Adamts14
NM_011526	-1.51	0.00005	0.00089	Tagln
NM_009141	-1.53	0.00005	0.00089	Cxcl5
NM_001025602	-1.53	0.00005	0.00089	Il1rl1
NM_011921	-1.53	0.00005	0.00089	Aldh1a7
NM_001013764	-1.55	0.00005	0.00089	Ces1a
NM_001039048	-1.57	0.00005	0.00089	Trim63
NM_001172424	-1.57	0.00005	0.00089	Dhrs3
NM_007562	-1.59	0.00005	0.00089	Bnc1
NM_026436	-1.61	0.00005	0.00089	Tmem86a
NM_010104	-1.66	0.00025	0.00374	Edn1
NM_007498	-1.67	0.00005	0.00089	Atf3
NM_009285	-1.68	0.00040	0.00567	Stc1
NM_011580	-1.73	0.00005	0.00089	Thbs1
NM_023608	-1.74	0.00005	0.00089	Gdpd2
NR_036452	-1.75	0.00005	0.00089	E230016K23Rik
NM_030728	-1.77	0.00005	0.00089	9930013L23Rik
NM_053106	-1.78	0.00005	0.00089	Lmod1
NM_027828	-1.80	0.00005	0.00089	Fam110c
NM_011118	-1.84	0.00005	0.00089	Prl2c3
NM_001042615	-1.87	0.00005	0.00089	Htra3
NM_013653	-1.88	0.00005	0.00089	Ccl5
NM_172799	-1.88	0.00005	0.00089	Ttll6
NM_011315	-1.93	0.00005	0.00089	Saa3
NM_010235	-1.93	0.00450	0.04299	Fosl1
NM_001112813	-1.94	0.00005	0.00089	Cacna1g
NM_020581	-2.07	0.00005	0.00089	Angptl4
NM_011333	-2.09	0.00005	0.00089	Ccl2
NM_011338	-2.14	0.00005	0.00089	Ccl9
NM_019759	-2.19	0.00005	0.00089	Dpt
NM_080639	-2.20	0.00005	0.00089	Timp4
NM_153170	-2.70	0.00025	0.00374	Slc36a2

Control vs Esrrb

gene	log2(fold_change)	p_value	q_value	Gene Symbol
NR_045442	#NAME?	0.00005	0.00135	1700019B21Rik
NM_001177437	inf	0.00005	0.00135	Mroh4
NR_040617	inf	0.00015	0.00348	Ptprtos
NM_009197	6.21	0.00005	0.00135	Slc16a2
NM_001252569	4.27	0.00005	0.00135	Serpina1a
NM_176973	4.09	0.00085	0.01440	Podxl2
NM_010266	3.95	0.00005	0.00135	Gda
NM_001256005	3.95	0.00005	0.00135	Gbp4
NM_001145807	3.86	0.00005	0.00135	Brinp3
NM_009244	3.66	0.00005	0.00135	Serpina1b
NM_001024139	3.51	0.00005	0.00135	Adamts15
NM_008270	3.51	0.00005	0.00135	Hoxb9
NM_009144	3.42	0.00005	0.00135	Sfrp2
NM_013554	3.24	0.00405	0.04649	Hoxd10
NM_033616	3.20	0.00005	0.00135	Csprs
NM_026142	3.16	0.00030	0.00614	3632451O06Rik
NM_001104547	3.06	0.00090	0.01512	Vmn2r96
NM_001037909	3.00	0.00005	0.00135	C130026I21Rik
NM_022018	2.74	0.00005	0.00135	Fam129a
NM_153131	2.66	0.00005	0.00135	Unc5a

NM_213615	2.63	0.00005	0.00135	A530032D15Rik
NM_001164566	2.62	0.00005	0.00135	Spats2l
NM_009285	2.54	0.00005	0.00135	Stc1
NM_030194	2.52	0.00005	0.00135	Sp110
NM_001285917	2.49	0.00005	0.00135	Dapk1
NM_025658	2.48	0.00005	0.00135	Ms4a4d
NR_028580	2.35	0.00015	0.00348	Gm10825
NM_008709	2.28	0.00060	0.01073	Mycn
NM_007730	2.17	0.00005	0.00135	Col12a1
NM_001079869	2.15	0.00010	0.00248	Hoxb3
NM_027852	2.09	0.00005	0.00135	Rarres2
NM_027406	2.08	0.00005	0.00135	Aldh1l1
NM_011909	2.05	0.00100	0.01637	Usp18
NM_177909	2.05	0.00045	0.00842	Slc9a9
NM_001039646	2.03	0.00005	0.00135	Gbp10
NM_008744	1.98	0.00005	0.00135	Ntn1
NM_001039647	1.97	0.00005	0.00135	Gbp11
NM_001081746	1.93	0.00005	0.00135	Gm7609
NM_001164107	1.93	0.00440	0.04935	Ripk3
NM_009331	1.91	0.00395	0.04573	Tcf7
NM_028426	1.90	0.00030	0.00614	3110007F17Rik
NM_001177752	1.85	0.00005	0.00135	Pfkfb3
NM_178738	1.80	0.00005	0.00135	Prss35
NM_001128606	1.75	0.00010	0.00248	Epb4.1
NM_080435	1.73	0.00055	0.01003	Adcy4
NM_199223	1.72	0.00005	0.00135	Rtn4rl2
NM_010050	1.71	0.00005	0.00135	Dio2
NM_080453	1.71	0.00005	0.00135	Mmp28
NM_175332	1.67	0.00250	0.03259	E130012A19Rik
NM_001109758	1.67	0.00015	0.00348	Bcan
NR_040297	1.66	0.00005	0.00135	Gm19757
NM_001205219	1.66	0.00005	0.00135	Sorbs2
NM_010658	1.66	0.00040	0.00768	Mafb
NM_173781	1.63	0.00005	0.00135	Rab6b
NR_015456	1.63	0.00360	0.04269	D7Ertd715e
NM_001098170	1.61	0.00005	0.00135	Pcdh10
NM_001122736	1.60	0.00005	0.00135	Igf2
NM_021451	1.57	0.00180	0.02553	Pmaip1
NM_001039209	1.54	0.00005	0.00135	Gm13152
NM_008524	1.49	0.00005	0.00135	Lum
NM_025492	1.49	0.00005	0.00135	1700020L24Rik
NM_018870	1.48	0.00220	0.02962	Pgam2
NM_019419	1.47	0.00005	0.00135	Arl6ip1
NM_029600	1.45	0.00005	0.00135	Abcc3
NM_028970	1.45	0.00360	0.04269	Rbm31y
NM_001029836	1.45	0.00005	0.00135	Npnt
NM_054098	1.44	0.00005	0.00135	Steap4
NM_172563	1.42	0.00005	0.00135	Hlf
NM_153170	1.42	0.00005	0.00135	Slc36a2
NM_001040085	1.40	0.00005	0.00135	Sytl2
NM_172648	1.40	0.00005	0.00135	Ifi205
NM_001162884	1.40	0.00005	0.00135	Igsf10
NM_013834	1.39	0.00005	0.00135	Sfrp1
NM_001009978	1.35	0.00015	0.00348	Pde1a
NM_177839	1.32	0.00005	0.00135	Tnn
NM_029353	1.31	0.00005	0.00135	Malsu1
NM_001164563	1.29	0.00005	0.00135	Amigo2
NM_001104550	1.27	0.00005	0.00135	Vmn2r98
NM_009899	1.27	0.00005	0.00135	Clca1
NR_040387	1.27	0.00010	0.00248	AI606473
NM_022312	1.26	0.00005	0.00135	Tnr

NM_001039239	1.25	0.00005	0.00135	Zfp808
NM_011882	1.25	0.00005	0.00135	Rnasel
NM_001111268	1.24	0.00005	0.00135	Grik2
NM_001122768	1.23	0.00005	0.00135	Lrrc8d
NM_025807	1.22	0.00005	0.00135	Slc16a9
NM_008817	1.22	0.00005	0.00135	Peg3
NM_013598	1.18	0.00005	0.00135	Kitl
NM_001039056	1.16	0.00005	0.00135	Kcnj15
NM_198100	1.15	0.00015	0.00348	Tbkbp1
NM_001033337	1.15	0.00030	0.00614	Ttc38
NM_027828	1.14	0.00005	0.00135	Fam110c
NM_013846	1.12	0.00005	0.00135	Ror2
NM_172298	1.12	0.00005	0.00135	Tshz3
NM_026358	1.11	0.00005	0.00135	Mgarp
NM_007400	1.10	0.00005	0.00135	Adam12
NM_001033335	1.09	0.00005	0.00135	Serpina3f
NM_001111274	1.09	0.00005	0.00135	Igf1
NM_177741	1.08	0.00005	0.00135	Ppp1r3b
NM_001080708	1.08	0.00005	0.00135	Fam65c
NM_181585	1.07	0.00005	0.00135	Pik3r3
NM_019759	1.05	0.00005	0.00135	Dpt
NM_001146351	1.04	0.00005	0.00135	Ephb6
NM_030261	1.02	0.00005	0.00135	Sesn3
NM_011526	-1.00	0.00005	0.00135	Tagln
NM_011845	-1.00	0.00005	0.00135	Mid2
NM_001013764	-1.02	0.00425	0.04815	Ces1a
NM_009802	-1.03	0.00265	0.03396	Car6
NM_009701	-1.07	0.00305	0.03751	Aqp5
NM_001145799	-1.09	0.00005	0.00135	Ctla2a
NM_029920	-1.12	0.00010	0.00248	Mtus2
NM_008380	-1.12	0.00095	0.01576	Inhba
NR_027819	-1.14	0.00005	0.00135	1810032008Rik
NM_001044740	-1.14	0.00005	0.00135	Slc7a2
NM_015759	-1.14	0.00005	0.00135	Fgd3
NM_181390	-1.15	0.00005	0.00135	Mustn1
NM_001199210	-1.19	0.00010	0.00248	Eva1c
NM_001111314	-1.19	0.00035	0.00693	Ngef
NM_013737	-1.19	0.00005	0.00135	Pla2g7
NM_019967	-1.20	0.00005	0.00135	Brinp1
NM_024406	-1.21	0.00005	0.00135	Fabp4
NM_178395	-1.23	0.00230	0.03063	Zdhhc2
NM_029881	-1.28	0.00005	0.00135	Tmem200a
NM_001037987	-1.28	0.00005	0.00135	Edil3
NM_007729	-1.28	0.00005	0.00135	Col11a1
NM_011766	-1.30	0.00005	0.00135	Zfpm2
NM_025404	-1.31	0.00375	0.04395	Arl4d
NM_170779	-1.34	0.00005	0.00135	Wwc1
NM_175122	-1.35	0.00005	0.00135	Rab39b
NM_001194940	-1.36	0.00005	0.00135	Dlc1
NM_001159965	-1.38	0.00005	0.00135	Ralgps2
NM_001081169	-1.38	0.00105	0.01694	Aspg
NM_029568	-1.38	0.00005	0.00135	Mfap4
NM_053106	-1.39	0.00005	0.00135	Lmod1
NM_011921	-1.39	0.00005	0.00135	Aldh1a7
NM_001285890	-1.40	0.00005	0.00135	Pde1b
NM_011077	-1.41	0.00005	0.00135	Phex
NM_001039220	-1.43	0.00005	0.00135	AI429214
NM_173007	-1.45	0.00005	0.00135	Tspan12
NM_013653	-1.48	0.00005	0.00135	Ccl5
NM_175271	-1.51	0.00005	0.00135	Lpar4
NM_177819	-1.52	0.00005	0.00135	Fam135b

NM_001008424	-1.54	0.00005	0.00135	Cdsn
NM_019503	-1.55	0.00005	0.00135	Fxyd1
NM_001024474	-1.58	0.00005	0.00135	Diras2
NM_001112725	-1.58	0.00175	0.02500	Aldh3a1
NM_053191	-1.58	0.00005	0.00135	Pi15
NM_001085376	-1.60	0.00005	0.00135	Pappa2
NM_010930	-1.61	0.00005	0.00135	Nov
NM_001163015	-1.62	0.00005	0.00135	Gprasp2
NM_001243072	-1.67	0.00005	0.00135	Sema3a
NM_009610	-1.70	0.00235	0.03106	Actg2
NM_007428	-1.76	0.00065	0.01146	Agt
NM_009655	-1.78	0.00005	0.00135	Alcam
NM_007621	-1.87	0.00005	0.00135	Cbr2
NM_021355	-1.91	0.00005	0.00135	Fmod
NM_001252330	-1.96	0.00005	0.00135	Slc6a15
NM_016917	-2.01	0.00005	0.00135	Slc40a1
NM_025429	-2.05	0.00005	0.00135	Serpinb1a
NM_013657	-2.10	0.00005	0.00135	Sema3c
NM_001285956	-2.15	0.00030	0.00614	Podn
NM_175454	-2.16	0.00020	0.00441	Hid1
NM_001039094	-2.29	0.00005	0.00135	Negr1
NM_023850	-2.39	0.00045	0.00842	Chst1
NM_001033228	-2.53	0.00005	0.00135	Itga1
NM_008862	-3.16	0.00005	0.00135	Pkia
NM_026271	-3.45	0.00005	0.00135	Fibin
NM_008904	-3.46	0.00005	0.00135	Ppargc1a
NM_011891	-3.69	0.00005	0.00135	Sgcd
NM_177346	-4.01	0.00005	0.00135	Gpr149
NM_001039934	-4.98	0.00095	0.01576	Map2

Control vs Esrrb+Hedgehog

gene	log2(fold_change)	p_value	q_value	Gene Symbol
NM_001110009	#NAME?	0.00065	0.00434	Apoc1
NM_175662	#NAME?	0.00005	0.00043	Hist2h2ac
NM_013650	#NAME?	0.00030	0.00221	S100a8
NM_029472	inf	0.00005	0.00043	Gstt4
NM_001177437	inf	0.00005	0.00043	Mroh4
NM_009236	inf	0.00005	0.00043	Sox18
NM_010296	9.46	0.00045	0.00316	Gli1
NM_009197	6.48	0.00005	0.00043	Slc16a2
NM_001044751	5.31	0.00005	0.00043	Hsd11b1
NM_152803	4.47	0.00540	0.02550	Hpse
NM_001252569	4.41	0.00005	0.00043	Serpina1a
NM_008957	4.09	0.00005	0.00043	Ptch1
NM_009244	4.07	0.00010	0.00082	Serpina1b
NM_013690	4.00	0.00005	0.00043	Tek
NM_013554	3.99	0.00215	0.01202	Hoxd10
NM_001099276	3.91	0.00005	0.00043	Pik3c2b
NM_176973	3.91	0.00150	0.00884	Podxl2
NM_001145807	3.90	0.00005	0.00043	Brinp3
NM_001024139	3.87	0.00005	0.00043	Adamts15
NM_001177752	3.85	0.00005	0.00043	Pfkfb3
NM_001256005	3.84	0.00005	0.00043	Gbp4
NM_016867	3.63	0.00005	0.00043	Gipc2
NM_016808	3.58	0.00005	0.00043	Usp2
NM_009117	3.48	0.00385	0.01955	Saa1
NM_026142	3.36	0.00090	0.00575	3632451O06Rik
NM_008623	3.34	0.00895	0.03795	Mpz
NM_010266	3.33	0.00005	0.00043	Gda
NM_153131	3.31	0.00005	0.00043	Unc5a
NM_033616	3.27	0.00005	0.00043	Csprs

NM_009641	3.22	0.00005	0.00043	Angpt4
NM_010050	3.19	0.00005	0.00043	Dio2
NM_022657	3.17	0.00005	0.00043	Fgf23
NM_007957	3.02	0.00870	0.03718	Esx1
NM_001037909	2.97	0.00005	0.00043	C130026I21Rik
NM_008709	2.88	0.00020	0.00154	Mycn
NM_001145886	2.85	0.00005	0.00043	Tiam1
NM_007539	2.84	0.00005	0.00043	Bdkrb1
NM_001177713	2.83	0.00005	0.00043	Cyp26b1
NM_001146180	2.74	0.00005	0.00043	Mtss1
NM_011623	2.73	0.00005	0.00043	Top2a
NM_007894	2.71	0.01070	0.04392	Ear1
NM_008270	2.70	0.00005	0.00043	Hoxb9
NM_007719	2.65	0.00035	0.00251	Ccr7
NM_001167680	2.62	0.00005	0.00043	Rhbdf2
NM_001164355	2.60	0.00005	0.00043	Ska1
NM_008489	2.59	0.00005	0.00043	Lbp
NM_009382	2.58	0.00005	0.00043	Thy1
NM_010517	2.57	0.00005	0.00043	Igfbp4
NM_001162506	2.57	0.00005	0.00043	Troap
NM_001081258	2.54	0.00005	0.00043	Kif14
NM_008380	2.51	0.00005	0.00043	Inhba
NM_146208	2.50	0.00005	0.00043	Neil3
NM_213615	2.49	0.00005	0.00043	A530032D15Rik
NM_145356	2.48	0.00005	0.00043	Zbtb7c
NM_010518	2.46	0.00005	0.00043	Igfbp5
NM_001281819	2.46	0.00005	0.00043	Ace
NM_001164107	2.45	0.00055	0.00376	Ripk3
NM_001081117	2.45	0.00005	0.00043	Mki67
NM_030172	2.42	0.00015	0.00119	Efcab11
NM_009860	2.41	0.00005	0.00043	Cdc25c
NM_153137	2.39	0.00005	0.00043	Traf3ip3
NM_054098	2.39	0.00005	0.00043	Steap4
NM_001081363	2.38	0.00005	0.00043	Cenpf
NM_026778	2.37	0.00005	0.00043	Cthrc1
NR_030721	2.37	0.00005	0.00043	Foxd2os
NM_031168	2.37	0.00005	0.00043	Il6
NM_001110265	2.37	0.00005	0.00043	Ttk
NM_016851	2.34	0.00005	0.00043	Irf6
NM_001122768	2.34	0.00005	0.00043	Lrrc8d
NM_197959	2.30	0.00005	0.00043	Kif18b
NM_030194	2.29	0.00005	0.00043	Sp110
NM_027930	2.29	0.00005	0.00043	Mtfr2
NR_030721	2.28	0.00005	0.00043	Foxd2os
NM_013538	2.27	0.00005	0.00043	Cdca3
NM_025429	2.26	0.00005	0.00043	Serpinb1a
NM_172563	2.26	0.00005	0.00043	Hlf
NM_001081746	2.26	0.00005	0.00043	Gm7609
NM_001110162	2.24	0.00005	0.00043	Cdca2
NM_001104547	2.24	0.00400	0.02014	Vmn2r96
NM_008235	2.22	0.00005	0.00043	Hes1
NM_178609	2.22	0.00005	0.00043	E2f7
NM_009791	2.22	0.00005	0.00043	Aspm
NM_008276	2.21	0.00005	0.00043	Hoxd8
NM_007730	2.21	0.00005	0.00043	Col12a1
NM_029617	2.21	0.00005	0.00043	Casc5
NM_011121	2.17	0.00005	0.00043	Plk1
NM_027975	2.17	0.00005	0.00043	Fam83d
NM_009115	2.17	0.00765	0.03362	S100b
NM_029835	2.16	0.00005	0.00043	Ticrr
NM_028039	2.16	0.00005	0.00043	Esco2

NM_027290	2.15	0.00005	0.00043	Mcm10
NM_001081085	2.15	0.00005	0.00043	Sapcd2
NM_001128606	2.15	0.00005	0.00043	Epb4.1
NM_001042421	2.14	0.00005	0.00043	Kntc1
NM_001033633	2.13	0.00005	0.00043	Slc2a13
NM_001014976	2.12	0.00005	0.00043	Espl1
NM_010658	2.12	0.00005	0.00043	Mafb
NM_173762	2.11	0.00005	0.00043	Cenpe
NM_010620	2.11	0.00005	0.00043	Kif15
NM_198423	2.10	0.00005	0.00043	Bahcc1
NM_172756	2.10	0.00005	0.00043	Ankle1
NM_001025779	2.10	0.00005	0.00043	Cdc6
NM_134117	2.08	0.00005	0.00043	Pkdcc
NM_001163495	2.08	0.00005	0.00043	Arhgap19
NM_001172092	2.07	0.00005	0.00043	Depdc1a
NM_010740	2.07	0.00005	0.00043	Cd93
NM_181589	2.07	0.00005	0.00043	Ckap2l
NM_016925	2.06	0.00005	0.00043	Fanca
NM_001012273	2.06	0.00005	0.00043	Birc5
NM_001271729	2.06	0.00005	0.00043	Tk1
NM_007708	2.05	0.00005	0.00043	Cit
NM_172453	2.05	0.00005	0.00043	Pif1
NM_022018	2.04	0.00005	0.00043	Fam129a
NM_001079869	2.04	0.00025	0.00187	Hoxb3
NM_134471	2.04	0.00005	0.00043	Kif2c
NM_024184	2.03	0.00005	0.00043	Asf1b
NM_025658	2.02	0.00005	0.00043	Ms4a4d
NM_198605	2.02	0.00005	0.00043	Ska3
NM_001013368	2.02	0.00005	0.00043	E2f8
NM_013552	2.02	0.00005	0.00043	Hmmr
NM_010468	2.02	0.00005	0.00043	Hoxd3
NM_001195025	2.02	0.00005	0.00043	Nuak2
NM_009144	2.02	0.00005	0.00043	Sfrp2
NM_144553	2.01	0.00005	0.00043	Dlgap5
NM_011132	2.01	0.00005	0.00043	Pole
NM_001159369	2.00	0.00005	0.00043	Polq
NM_175554	2.00	0.00005	0.00043	Clspn
NM_019444	1.99	0.00005	0.00043	Ramp2
NM_177343	1.99	0.00005	0.00043	Camk1d
NM_010790	1.99	0.00005	0.00043	Melk
NM_009185	1.99	0.00005	0.00043	Stil
NM_013555	1.99	0.00005	0.00043	Hoxd9
NM_001113179	1.99	0.00005	0.00043	Bub1
NM_008021	1.98	0.00005	0.00043	Foxm1
NM_009013	1.98	0.00005	0.00043	Rad51ap1
NM_010615	1.98	0.00005	0.00043	Kif11
NM_028481	1.98	0.00005	0.00043	Ccdc18
NM_009252	1.98	0.00005	0.00043	Serpina3n
NM_001113460	1.97	0.00005	0.00043	Tec
NM_172671	1.97	0.00005	0.00043	Lgr4
NM_172301	1.97	0.00005	0.00043	Ccnb1
NM_176982	1.96	0.00005	0.00043	Fbxo48
NM_028870	1.96	0.00005	0.00043	Cltb
NM_009764	1.95	0.00005	0.00043	Brca1
NM_011496	1.95	0.00005	0.00043	Aurkb
NM_001168672	1.95	0.00005	0.00043	Gtse1
NM_010892	1.95	0.00005	0.00043	Nek2
NM_026507	1.95	0.00005	0.00043	Zwilch
NM_008651	1.94	0.00005	0.00043	Mybl1
NR_027817	1.93	0.00385	0.01955	1500011B03Rik
NM_008185	1.93	0.00005	0.00043	Gstt1

NM_009828	1.92	0.00005	0.00043	Ccna2
NM_144818	1.92	0.00005	0.00043	Ncaph
NM_001141975	1.92	0.00005	0.00043	Tpx2
NM_144526	1.92	0.00005	0.00043	Fam64a
NM_009773	1.91	0.00005	0.00043	Bub1b
NM_175563	1.91	0.00005	0.00043	Prr11
NM_007681	1.90	0.00005	0.00043	Cenpa
NM_001039646	1.90	0.00005	0.00043	Gbp10
NM_026410	1.90	0.00005	0.00043	Cdca5
NM_011909	1.90	0.00265	0.01429	Usp18
NM_026412	1.90	0.00005	0.00043	Knstrn
NM_001013377	1.90	0.00025	0.00187	Arhgef39
NM_001285997	1.89	0.00005	0.00043	Prc1
NM_011497	1.89	0.00005	0.00043	Aurka
NM_134041	1.89	0.00005	0.00043	4930427A07Rik
NM_001199123	1.89	0.00005	0.00043	Spc25
NM_007993	1.89	0.00005	0.00043	Fbn1
NM_023223	1.89	0.00005	0.00043	Cdc20
NM_009437	1.88	0.00040	0.00284	Tst
NM_001285917	1.88	0.00005	0.00043	Dapk1
NM_001199696	1.87	0.00005	0.00043	Bai2
NM_001033484	1.87	0.00005	0.00043	Iqgap3
NM_145924	1.87	0.00005	0.00043	Cenpi
NM_183046	1.86	0.00005	0.00043	Kif20b
NM_178421	1.85	0.00005	0.00043	Nanos1
NM_023284	1.85	0.00005	0.00043	Nuf2
NM_008055	1.85	0.00005	0.00043	Fzd4
NM_146171	1.85	0.00005	0.00043	Ncapd2
NM_145588	1.84	0.00005	0.00043	Kif22
NM_033270	1.84	0.00050	0.00346	E2f6
NM_153762	1.84	0.00005	0.00043	Rnf26
NM_011369	1.83	0.00005	0.00043	Shcbp1
NM_001033141	1.83	0.00485	0.02336	Ecscr
NM_008593	1.83	0.00070	0.00463	Foxd2
NM_080435	1.83	0.00065	0.00434	Adcy4
NM_026560	1.82	0.00005	0.00043	Cdca8
NR_001592	1.82	0.00005	0.00043	H19
NR_015456	1.82	0.00230	0.01272	D7Ertd715e
NM_019753	1.82	0.00005	0.00043	Cdh17
NM_001042653	1.80	0.00160	0.00936	Oip5
NM_001003919	1.80	0.00005	0.00043	Ddx11
NM_001167743	1.80	0.00005	0.00043	Slfn8
NM_010657	1.80	0.00005	0.00043	Hivep3
NM_001164566	1.79	0.00005	0.00043	Spats2l
NM_001033464	1.79	0.00005	0.00043	Efcab4b
NM_021342	1.79	0.00005	0.00043	Kcne4
NM_001177867	1.79	0.00005	0.00043	Sgol2
NM_198654	1.79	0.00005	0.00043	Nsl1
NM_024245	1.78	0.00005	0.00043	Kif23
NM_017407	1.78	0.00005	0.00043	Spag5
NM_001080995	1.78	0.00005	0.00043	4632434I11Rik
NM_026785	1.78	0.00005	0.00043	Ube2c
NM_178309	1.77	0.00005	0.00043	Brip1
NM_029249	1.76	0.00005	0.00043	Parpbp
NM_001001804	1.76	0.01085	0.04440	Ephx4
NM_001131054	1.76	0.00005	0.00043	Pttg1
NM_023209	1.76	0.00005	0.00043	Pbk
NM_001195298	1.76	0.00005	0.00043	Kifc1
NM_023294	1.75	0.00005	0.00043	Ndc80
NM_001098170	1.75	0.00005	0.00043	Pcdh10
NM_001177625	1.74	0.00005	0.00043	Ect2

NM_016854	1.74	0.00005	0.00043	Ppp1r3c
NM_001127259	1.74	0.00005	0.00043	Trp63
NM_001081125	1.74	0.00005	0.00043	Gli2
NM_001159518	1.74	0.00005	0.00043	Igfbp7
NM_177331	1.72	0.00005	0.00043	Gen1
NM_001039556	1.72	0.00005	0.00043	Rad54b
NM_153544	1.71	0.00005	0.00043	BC030867
NM_172578	1.71	0.00005	0.00043	Mis18bp1
NM_146235	1.71	0.00005	0.00043	Ercc6l
NM_001033455	1.71	0.00040	0.00284	Ccdc27
NM_019641	1.70	0.00005	0.00043	Stmn1
NM_028232	1.70	0.00005	0.00043	Sgol1
NM_009931	1.69	0.00005	0.00043	Col4a1
NM_025979	1.69	0.00005	0.00043	Mastl
NM_029482	1.69	0.00005	0.00043	4930579G24Rik
NM_058214	1.68	0.00005	0.00043	Recql4
NM_001033331	1.68	0.00005	0.00043	Gas2l3
NM_001081406	1.68	0.00065	0.00434	Lrr1
NM_019971	1.68	0.00005	0.00043	Pdgfc
NM_028666	1.67	0.00005	0.00043	Fam110a
NM_023058	1.65	0.00005	0.00043	Pkmyt1
NM_001122958	1.65	0.00635	0.02902	Rad54l
NM_001032413	1.65	0.00005	0.00043	Pear1
NM_008652	1.65	0.00005	0.00043	Mybl2
NM_028222	1.65	0.00005	0.00043	Cdkn3
NM_146186	1.65	0.00005	0.00043	Wdr62
NM_001033244	1.64	0.00005	0.00043	Fancd2
NM_178252	1.64	0.00005	0.00043	Arhgap33
NM_001163763	1.64	0.00005	0.00043	Tcf19
NM_001040435	1.63	0.00005	0.00043	Tacc3
NM_026743	1.63	0.00005	0.00043	Tspan11
NM_008446	1.63	0.00005	0.00043	Kif4
NM_019670	1.63	0.00005	0.00043	Diap3
NM_198622	1.63	0.00005	0.00043	H1fx
NM_007525	1.63	0.00005	0.00043	Bard1
NM_001080158	1.63	0.00005	0.00043	Cenpm
NM_010353	1.62	0.00005	0.00043	Gsg2
NM_001163256	1.61	0.00005	0.00043	Fblim1
NM_139303	1.61	0.00005	0.00043	Kif18a
NM_009253	1.61	0.00005	0.00043	Serpina3m
NM_001163793	1.61	0.00005	0.00043	C530008M17Rik
NR_033780	1.61	0.00005	0.00043	2810001G20Rik
NM_172564	1.61	0.00005	0.00043	Tns4
NM_145409	1.60	0.00005	0.00043	Chtf18
NM_053173	1.60	0.00005	0.00043	Kifc5b
NM_001012766	1.60	0.00170	0.00984	Ear12
NM_001271705	1.60	0.00975	0.04064	Pgf
NM_175265	1.60	0.00005	0.00043	Bora
NR_024720	1.60	0.00005	0.00043	2700099C18Rik
NM_016692	1.60	0.00005	0.00043	Incenp
NM_153524	1.59	0.00565	0.02642	Mrgpra4
NM_001146081	1.59	0.00005	0.00043	Fancb
NM_019438	1.58	0.00005	0.00043	Ncapg
NM_029975	1.58	0.00005	0.00043	Ulbp1
NM_178683	1.57	0.00005	0.00043	Depdc1b
NM_001145827	1.57	0.00005	0.00043	Stk40
NM_025415	1.57	0.00005	0.00043	Cks2
NM_001252055	1.57	0.00005	0.00043	Ly6c1
NM_001163356	1.56	0.00010	0.00082	Fam212b
NM_013571	1.56	0.00005	0.00043	Ksr1
NM_001142952	1.56	0.00235	0.01295	Fam46c

NM_177372	1.56	0.00005	0.00043	Dna2
NM_001164362	1.56	0.00005	0.00043	Cep55
NM_001109747	1.55	0.00005	0.00043	Cenpw
NM_021886	1.55	0.00005	0.00043	Cenph
NM_007634	1.55	0.00005	0.00043	Ccnf
NM_001163476	1.55	0.00005	0.00043	Gins1
NM_001085549	1.55	0.00005	0.00043	Trabd2b
NM_001111274	1.54	0.00005	0.00043	Igf1
NM_025995	1.54	0.00005	0.00043	Fbxo5
NM_001009978	1.54	0.00005	0.00043	Pde1a
NM_012012	1.54	0.00005	0.00043	Exo1
NM_172145	1.54	0.00005	0.00043	Eva1b
NM_029766	1.53	0.00005	0.00043	Dtl
NM_181848	1.53	0.00005	0.00043	Optn
NM_008744	1.53	0.00005	0.00043	Ntn1
NM_009932	1.52	0.00005	0.00043	Col4a2
NM_181815	1.52	0.00005	0.00043	Cep128
NM_001159930	1.52	0.00005	0.00043	Cenpl
NM_008017	1.52	0.00005	0.00043	Smc2
NM_028390	1.52	0.00005	0.00043	Anln
NM_026613	1.51	0.00005	0.00043	Ccdc34
NM_009104	1.51	0.00005	0.00043	Rrm2
NM_028013	1.51	0.00005	0.00043	Endod1
NM_001164564	1.50	0.00005	0.00043	Egfl7
NM_027435	1.50	0.00005	0.00043	Atad2
NM_001253808	1.50	0.00005	0.00043	Racgap1
NM_026301	1.49	0.00035	0.00251	Rnf125
NM_001166406	1.49	0.00005	0.00043	Kif20a
NM_019521	1.49	0.00005	0.00043	Gas6
NM_007695	1.49	0.00005	0.00043	Chil1
NM_173781	1.49	0.00005	0.00043	Rab6b
NM_028349	1.48	0.00005	0.00043	Sass6
NM_013807	1.48	0.00005	0.00043	Plk3
NM_027182	1.46	0.00005	0.00043	Trip13
NR_023846	1.46	0.00060	0.00405	Peg3os
NM_011234	1.46	0.00005	0.00043	Rad51
NR_040297	1.46	0.00010	0.00082	Gm19757
NM_008716	1.46	0.00005	0.00043	Notch3
NM_146116	1.45	0.00005	0.00043	Tubb4b
NM_001177794	1.45	0.00005	0.00043	Sertad4
NM_008209	1.45	0.00005	0.00043	Mr1
NM_001080708	1.45	0.00005	0.00043	Fam65c
NR_033736	1.45	0.00005	0.00043	E330020D12Rik
NM_017370	1.45	0.00005	0.00043	Hp
NM_026515	1.45	0.00005	0.00043	2810417H13Rik
NM_080467	1.44	0.00005	0.00043	Atp6v0a4
NM_001039209	1.44	0.00015	0.00119	Gm13152
NM_175332	1.44	0.00615	0.02830	E130012A19Rik
NM_001081099	1.44	0.00055	0.00376	Clhc1
NM_027354	1.43	0.00005	0.00043	Poc1a
NM_178804	1.43	0.00005	0.00043	Slit2
NM_001033217	1.43	0.00005	0.00043	Prickle1
NM_008817	1.43	0.00005	0.00043	Peg3
NM_001190717	1.42	0.00005	0.00043	Dbf4
NM_001004140	1.42	0.00005	0.00043	Ckap2
NM_019419	1.42	0.00005	0.00043	Arl6ip1
NM_013529	1.42	0.00005	0.00043	Gfpt2
NM_001029838	1.42	0.00005	0.00043	Pknox2
NM_008458	1.42	0.00035	0.00251	Serpina3c
NM_146238	1.41	0.00005	0.00043	Gemin8
NM_001205219	1.41	0.00005	0.00043	Sorbs2

NM_028261	1.41	0.00005	0.00043	Tmem173
NR_015469	1.40	0.00005	0.00043	2810442I21Rik
NM_001037134	1.40	0.00005	0.00043	Ccne2
NM_001163359	1.39	0.00005	0.00043	Fignl1
NM_181416	1.39	0.00005	0.00043	Arhgap11a
NM_001114386	1.39	0.00005	0.00043	Nedd4l
NM_008566	1.39	0.00005	0.00043	Mcm5
NM_010049	1.39	0.00005	0.00043	Dhfr
NM_011495	1.39	0.00005	0.00043	Plk4
NR_037955	1.38	0.00005	0.00043	1190002F15Rik
NM_026282	1.38	0.00005	0.00043	Spc24
NM_010655	1.38	0.00005	0.00043	Kpna2
NM_001163480	1.38	0.00005	0.00043	Neurl1a
NM_010726	1.38	0.00005	0.00043	Phyh
NM_012018	1.37	0.00005	0.00043	Cep110
NM_007659	1.37	0.00005	0.00043	Cdk1
NM_008882	1.37	0.00005	0.00043	Plxna2
NM_001122736	1.37	0.00005	0.00043	Igf2
NM_025566	1.37	0.00015	0.00119	Tnfaip8l1
NM_011264	1.37	0.00005	0.00043	Rev3l
NM_001111268	1.36	0.00005	0.00043	Grik2
NM_028131	1.36	0.00005	0.00043	Cenpn
NM_001159646	1.36	0.00005	0.00043	Dut
NM_172598	1.36	0.00005	0.00043	Wdhd1
NM_016719	1.35	0.00005	0.00043	Grb14
NM_028006	1.35	0.00005	0.00043	Tube1
NM_001033335	1.35	0.00005	0.00043	Serpina3f
NM_019499	1.35	0.00005	0.00043	Mad2l1
NM_013733	1.35	0.00005	0.00043	Chaf1a
NM_001162533	1.34	0.00125	0.00762	Sh3d21
NM_008737	1.34	0.00005	0.00043	Nrp1
NM_133897	1.34	0.00005	0.00043	Lrrc8c
NM_144786	1.34	0.00005	0.00043	Ggt7
NR_038009	1.33	0.00005	0.00043	2810408I11Rik
NM_001161623	1.32	0.00005	0.00043	Cdc45
NM_153805	1.32	0.00010	0.00082	Pkn3
NM_080453	1.32	0.00005	0.00043	Mmp28
NM_027263	1.32	0.00005	0.00043	Apitd1
NM_183089	1.32	0.00005	0.00043	Dscc1
NM_027411	1.32	0.00005	0.00043	Spdl1
NM_146174	1.32	0.00005	0.00043	Fam115c
NM_001029856	1.32	0.00005	0.00043	Atad5
NM_027225	1.31	0.00005	0.00043	Cobll1
NM_007955	1.30	0.00005	0.00043	Ptprv
NM_029842	1.30	0.00265	0.01429	Kdm8
NM_001111078	1.30	0.00005	0.00043	Uhrf1
NM_009769	1.30	0.00005	0.00043	Klf5
NM_001081695	1.30	0.00390	0.01974	Dnmt3l
NM_172616	1.30	0.00005	0.00043	C330027C09Rik
NM_001039543	1.30	0.00005	0.00043	Mlf1
NM_011452	1.30	0.00150	0.00884	Serpinb9b
NM_010637	1.29	0.00005	0.00043	Klf4
NM_001039647	1.29	0.00005	0.00043	Gbp11
NM_008966	1.29	0.00005	0.00043	Ptgfr
NM_008921	1.29	0.00005	0.00043	Prim1
NM_178576	1.28	0.01195	0.04798	Cpsf4
NM_178113	1.28	0.00300	0.01592	Ncapd3
NM_008817	1.28	0.00005	0.00043	Peg3
NM_028128	1.28	0.00005	0.00043	Rfc5
NM_009320	1.28	0.00005	0.00043	Slc6a6
NM_001136082	1.27	0.00005	0.00043	Timeless

NM_009516	1.27	0.00005	0.00043	Wee1
NM_025492	1.27	0.00020	0.00154	1700020L24Rik
NM_021790	1.27	0.00410	0.02055	Cenpk
NM_011427	1.27	0.00005	0.00043	Snai1
NM_001271566	1.27	0.00005	0.00043	Cdc7
NM_001167730	1.27	0.00005	0.00043	Rad18
NM_080640	1.26	0.00005	0.00043	Baalc
NM_201364	1.26	0.00005	0.00043	BC055324
NM_027852	1.26	0.00005	0.00043	Rarres2
NM_011634	1.25	0.00005	0.00043	Traip
NM_025642	1.25	0.00005	0.00043	Mis18a
NM_001083188	1.25	0.00005	0.00043	Lig1
NM_172505	1.25	0.00005	0.00043	A730008H23Rik
NM_145946	1.25	0.00005	0.00043	Fanci
NM_199467	1.25	0.00005	0.00043	Mms22l
NM_184109	1.25	0.00005	0.00043	Rtl1
NM_009285	1.24	0.00005	0.00043	Stc1
NM_007633	1.24	0.00005	0.00043	Ccne1
NM_001081317	1.24	0.00005	0.00043	Zfand4
NM_027871	1.24	0.00005	0.00043	Arhgef3
NM_001271614	1.24	0.00005	0.00043	Fen1
NM_001113350	1.24	0.00025	0.00187	Xkr5
NM_139197	1.24	0.00905	0.03830	Gbgt1
NM_133762	1.23	0.00005	0.00043	Ncapg2
NM_183208	1.23	0.00005	0.00043	Zmiz1
NM_134000	1.23	0.00035	0.00251	Traf3ip2
NM_001278115	1.22	0.00005	0.00043	Ube2t
NM_010791	1.22	0.00005	0.00043	Meox1
NM_180588	1.22	0.00005	0.00043	Reep4
NM_146061	1.22	0.00005	0.00043	Prr5
NM_028829	1.22	0.00005	0.00043	Paqr8
NM_021793	1.22	0.00005	0.00043	Tmem8
NM_001168318	1.22	0.00005	0.00043	Scara5
NM_183297	1.21	0.00615	0.02830	Nxph4
NM_013691	1.21	0.00005	0.00043	Thbs3
NM_021288	1.21	0.00005	0.00043	Tyms
NM_080850	1.21	0.00005	0.00043	Pask
NM_133687	1.21	0.00005	0.00043	Cxxc5
NM_024241	1.21	0.00005	0.00043	Kif24
NM_028679	1.21	0.00005	0.00043	Irak3
NM_001033550	1.21	0.00005	0.00043	Lrrc8b
NM_177603	1.20	0.00740	0.03279	Frat2
NM_001163471	1.20	0.00005	0.00043	Hectd2
NM_001174155	1.20	0.00035	0.00251	Rasgrp4
NM_011015	1.19	0.00005	0.00043	Orc1
NM_001242396	1.19	0.00005	0.00043	Jmjd1c
NM_001042527	1.19	0.00005	0.00043	Blm
NM_001111075	1.18	0.00005	0.00043	Cdc25b
NM_007691	1.18	0.00015	0.00119	Chek1
NM_001040631	1.18	0.00005	0.00043	C1qtnf5
NM_001161665	1.18	0.00005	0.00043	Kif26b
NM_001080929	1.18	0.00005	0.00043	Cdr2l
NM_020567	1.17	0.00005	0.00043	Gmnn
NM_025928	1.17	0.00005	0.00043	Pmf1
NM_144881	1.17	0.00005	0.00043	Hhat
NM_001204915	1.17	0.00005	0.00043	Reep3
NM_008563	1.16	0.00005	0.00043	Mcm3
NM_175429	1.16	0.00005	0.00043	Kctd12b
NM_173396	1.16	0.00005	0.00043	Tgif2
NM_025590	1.16	0.00005	0.00043	Acot11
NM_025676	1.16	0.00005	0.00043	Mcm8

NM_028083	1.16	0.00005	0.00043	Chaf1b
NM_001042652	1.15	0.00005	0.00043	Nusap1
NM_022724	1.15	0.00005	0.00043	Suv39h2
NM_001162365	1.15	0.00115	0.00709	Ptk2b
NM_009016	1.15	0.00005	0.00043	Raet1a
NM_053204	1.15	0.00005	0.00043	Erc1
NM_001162932	1.15	0.00010	0.00082	Rmi2
NM_178932	1.15	0.00885	0.03762	Aoc2
NM_028175	1.14	0.00005	0.00043	Lrrc8e
NM_001103182	1.14	0.00005	0.00043	Lin9
NM_025853	1.13	0.00005	0.00043	Dsn1
NM_009542	1.13	0.00005	0.00043	Zfp101
NM_016710	1.13	0.00005	0.00043	Hmgn5
NM_008567	1.13	0.00005	0.00043	Mcm6
NM_008344	1.13	0.00005	0.00043	Igfbp6
NM_010387	1.13	0.00005	0.00043	H2-DMb1
NM_001081091	1.12	0.00005	0.00043	Cep152
NM_001040111	1.12	0.00005	0.00043	Arap1
NR_015556	1.12	0.00095	0.00602	2610035D17Rik
NM_011854	1.12	0.00320	0.01678	Oasl2
NM_001081249	1.12	0.00005	0.00043	Vcan
NM_016782	1.12	0.00130	0.00786	Cntnap1
NM_001005341	1.12	0.00005	0.00043	Ypel2
NM_001252600	1.12	0.00345	0.01785	Irf7
NM_011131	1.11	0.00005	0.00043	Pold1
NM_001162537	1.11	0.00005	0.00043	9330159F19Rik
NM_001193305	1.11	0.00005	0.00043	Mical2
NM_001081001	1.11	0.00005	0.00043	Brca2
NM_013778	1.11	0.00105	0.00655	Akr1c13
NM_177710	1.10	0.00005	0.00043	Ssh2
NM_019483	1.10	0.00055	0.00376	Smad9
NM_174848	1.10	0.00005	0.00043	Crybg3
NM_001276248	1.10	0.00005	0.00043	Cp
NM_001113211	1.09	0.00005	0.00043	Tmem194
NM_013673	1.09	0.00005	0.00043	Sp100
NM_001139516	1.09	0.00005	0.00043	Rbl1
NM_001025365	1.09	0.00005	0.00043	Miip
NM_011962	1.09	0.00005	0.00043	Plod3
NM_177702	1.09	0.00125	0.00762	4833427G06Rik
NM_008892	1.09	0.00005	0.00043	Pola1
NM_016756	1.09	0.00005	0.00043	Cdk2
NM_001081445	1.08	0.00005	0.00043	Ncam1
NM_001003960	1.08	0.00005	0.00043	Dnmt3b
NM_033602	1.08	0.00005	0.00043	Peli2
NM_028266	1.07	0.00005	0.00043	Col16a1
NM_001077398	1.07	0.00005	0.00043	Ldb2
NM_027556	1.07	0.00005	0.00043	Cep192
NM_001033378	1.07	0.00020	0.00154	A430078G23Rik
NM_019518	1.06	0.00005	0.00043	Grasp
NM_001025163	1.06	0.00020	0.00154	Zfp78
NM_009895	1.06	0.00005	0.00043	Cish
NM_016681	1.05	0.00005	0.00043	Chek2
NM_009378	1.05	0.00005	0.00043	Thbd
NR_033599	1.05	0.00005	0.00043	Gm6682
NM_030889	1.05	0.00005	0.00043	Sorcs2
NM_172134	1.04	0.00005	0.00043	Pdxk
NM_001081364	1.04	0.00005	0.00043	Arhgap21
NM_001146174	1.04	0.00005	0.00043	Rangap1
NM_001115010	1.04	0.00005	0.00043	Lin54
NM_021305	1.03	0.00005	0.00043	Sec61a2
NM_053072	1.03	0.00005	0.00043	Fgd6

NM_172911	1.03	0.00005	0.00043	D8Ertd82e
NM_175382	1.03	0.01220	0.04880	Fam72a
NM_021361	1.03	0.00005	0.00043	Nova1
NM_022312	1.03	0.00005	0.00043	Tnr
NM_001040691	1.02	0.00005	0.00043	Ung
NM_134024	1.02	0.00005	0.00043	Tubg1
NM_011858	1.02	0.00005	0.00043	Tenm4
NM_177409	1.02	0.00005	0.00043	Tram2
NM_173386	1.02	0.00005	0.00043	Mb21d1
NM_001039150	1.02	0.00005	0.00043	Cd44
NM_001168304	1.02	0.00005	0.00043	Cdk19
NM_011284	1.02	0.00005	0.00043	Rpa2
NM_008548	1.02	0.00005	0.00043	Man1a
NM_001285945	1.02	0.00005	0.00043	Zranb3
NM_153287	1.01	0.00050	0.00346	Csrnp1
NM_030198	1.01	0.00005	0.00043	Gins3
NM_001172472	1.01	0.00005	0.00043	Sphk1
NM_008565	1.01	0.00005	0.00043	Mcm4
NM_008922	1.01	0.00005	0.00043	Prim2
NM_001272057	1.01	0.00305	0.01611	Adam5
NM_001282992	1.01	0.00005	0.00043	Pcnt
NM_011625	1.00	0.00005	0.00043	Ppp1r13b
NM_009103	1.00	0.00005	0.00043	Rrm1
NM_018827	1.00	0.00005	0.00043	Crlf1
NM_177271	1.00	0.00005	0.00043	Samd5
NM_026640	1.00	0.00005	0.00043	Fam111a
NM_001040397	-1.00	0.00005	0.00043	Filip1l
NM_181344	-1.00	0.00005	0.00043	C1rl
NM_153529	-1.00	0.00005	0.00043	Nrn1
NM_027950	-1.01	0.00005	0.00043	Osgin1
NM_001136086	-1.02	0.00005	0.00043	Dpysl3
NM_007739	-1.02	0.00005	0.00043	Col8a1
NM_012030	-1.02	0.00005	0.00043	Slc9a3r1
NM_130449	-1.02	0.00005	0.00043	Colec12
NM_001159394	-1.03	0.00005	0.00043	Nfkbiz
NM_153782	-1.03	0.00005	0.00043	Fam20a
NM_001081277	-1.03	0.00290	0.01545	Ak5
NM_001252059	-1.03	0.00005	0.00043	Cacna1a
NM_001256081	-1.03	0.00005	0.00043	Myo7a
NM_001160112	-1.03	0.00005	0.00043	Foxg1
NM_001081433	-1.04	0.00005	0.00043	Ankrd44
NM_175454	-1.05	0.00455	0.02225	Hid1
NM_001042779	-1.06	0.00005	0.00043	Sema3b
NM_026235	-1.07	0.00405	0.02036	Larp6
NM_009368	-1.07	0.00005	0.00043	Tgfb3
NM_001252568	-1.07	0.00005	0.00043	Phyhd1
NM_001204201	-1.07	0.00005	0.00043	Spp1
NM_001281527	-1.08	0.00005	0.00043	Gm7361
NM_025770	-1.08	0.00005	0.00043	Atg10
NM_023386	-1.09	0.00840	0.03620	Rtp4
NM_001109988	-1.09	0.00005	0.00043	Nrep
NM_133764	-1.09	0.00005	0.00043	Atp6v0e2
NM_019967	-1.09	0.00005	0.00043	Brinp1
NM_145144	-1.09	0.00005	0.00043	Aif1l
NM_054056	-1.09	0.00005	0.00043	Pawr
NM_144896	-1.10	0.00005	0.00043	Pet112
NM_001136077	-1.10	0.00005	0.00043	Enpp2
NM_001081756	-1.10	0.00025	0.00187	Nckap5
NM_029568	-1.11	0.00005	0.00043	Mfap4
NR_045098	-1.12	0.00005	0.00043	Gm2115
NM_001271531	-1.12	0.00005	0.00043	Insig2

NM_020578	-1.12	0.00005	0.00043	Ehd3
NM_013495	-1.13	0.00005	0.00043	Cpt1a
NM_019919	-1.13	0.00005	0.00043	Ltbp1
NM_019759	-1.14	0.00005	0.00043	Dpt
NM_010774	-1.15	0.00005	0.00043	Mbd4
NM_178591	-1.17	0.00005	0.00043	Nrg1
NM_054041	-1.17	0.00005	0.00043	Antxr1
NM_013467	-1.18	0.00005	0.00043	Aldh1a1
NM_028561	-1.18	0.00005	0.00043	Speer4b
NM_009189	-1.18	0.00005	0.00043	Six1
NM_001025602	-1.20	0.00005	0.00043	Il1rl1
NM_023455	-1.20	0.00005	0.00043	Nat8
NM_025427	-1.20	0.00005	0.00043	Rgcc
NM_007562	-1.20	0.00005	0.00043	Bnc1
NM_182808	-1.20	0.00005	0.00043	Fam19a1
NM_001081169	-1.21	0.00215	0.01202	Aspg
NM_001013019	-1.21	0.00005	0.00043	Lrrn4cl
NM_013654	-1.21	0.00005	0.00043	Ccl7
NM_001135100	-1.22	0.00005	0.00043	Il34
NM_027759	-1.23	0.00775	0.03396	Fsip1
NM_009121	-1.23	0.00005	0.00043	Sat1
NM_001044740	-1.23	0.00005	0.00043	Slc7a2
NM_001177810	-1.23	0.00005	0.00043	Rapgef3
NM_177157	-1.24	0.00005	0.00043	Gchfr
NM_001081307	-1.24	0.00005	0.00043	Ppp1r12b
NM_001077189	-1.26	0.00815	0.03537	Fcgr2b
NM_001190911	-1.26	0.00005	0.00043	Kirrel3
NM_009154	-1.27	0.00005	0.00043	Sema5a
NM_001271483	-1.27	0.00005	0.00043	Clip4
NM_170779	-1.27	0.00005	0.00043	Wwc1
NM_010415	-1.28	0.00005	0.00043	Hbegf
NM_008349	-1.28	0.00005	0.00043	Il10rb
NR_033474	-1.29	0.00005	0.00043	2810433D01Rik
NM_148951	-1.29	0.00035	0.00251	Gipc3
NM_001113553	-1.30	0.00005	0.00043	Irak2
NM_001177319	-1.31	0.00005	0.00043	Tfpi
NM_001254747	-1.32	0.00915	0.03867	Il15
NM_173008	-1.33	0.00005	0.00043	Ssc5d
NM_174857	-1.33	0.00005	0.00043	Mamdc2
NM_026416	-1.34	0.00025	0.00187	S100a16
NM_001083810	-1.34	0.00005	0.00043	Prr5l
NM_013653	-1.34	0.00010	0.00082	Ccl5
NM_001272078	-1.35	0.00005	0.00043	Trem2
NM_015759	-1.36	0.00005	0.00043	Fgd3
NM_001145965	-1.36	0.00005	0.00043	Dlgap2
NM_130858	-1.37	0.00725	0.03227	Nxph3
NM_181390	-1.37	0.00005	0.00043	Mustn1
NM_013602	-1.38	0.00005	0.00043	Mt1
NM_001286653	-1.39	0.00005	0.00043	Rcan2
NM_007498	-1.39	0.00005	0.00043	Atf3
NM_153170	-1.40	0.00110	0.00681	Slc36a2
NM_001112813	-1.40	0.00005	0.00043	Cacna1g
NM_001285890	-1.41	0.00005	0.00043	Pde1b
NM_026436	-1.42	0.00005	0.00043	Tmem86a
NM_007413	-1.42	0.00005	0.00043	Adora2b
NM_009369	-1.42	0.00005	0.00043	Tgfbi
NM_001166580	-1.44	0.00005	0.00043	8430408G22Rik
NM_009141	-1.44	0.00005	0.00043	Cxcl5
NM_001103177	-1.44	0.00005	0.00043	Ablim1
NM_001285956	-1.44	0.00100	0.00627	Podn
NM_001163015	-1.45	0.00005	0.00043	Gprasp2

NM_008973	-1.46	0.00005	0.00043	Ptn
NM_001164316	-1.47	0.00005	0.00043	Ccser1
NM_053109	-1.48	0.00005	0.00043	Clec2d
NM_001039220	-1.48	0.00005	0.00043	AI429214
NM_001172424	-1.49	0.00005	0.00043	Dhrs3
NM_001243072	-1.51	0.00005	0.00043	Sema3a
NM_138649	-1.51	0.01005	0.04170	Syt17
NM_011766	-1.51	0.00005	0.00043	Zfpm2
NM_001112725	-1.53	0.00325	0.01700	Aldh3a1
NM_175271	-1.53	0.00005	0.00043	Lpar4
NM_010930	-1.53	0.00005	0.00043	Nov
NM_010235	-1.53	0.00130	0.00786	Fosl1
NM_009162	-1.56	0.00820	0.03552	Scg5
NM_007392	-1.56	0.00005	0.00043	Acta2
NM_173007	-1.57	0.00005	0.00043	Tspan12
NM_008630	-1.58	0.00005	0.00043	Mt2
NM_001039094	-1.59	0.00005	0.00043	Negr1
NM_008046	-1.59	0.00005	0.00043	Fst
NM_001194940	-1.59	0.00005	0.00043	Dlc1
NM_177191	-1.59	0.00010	0.00082	Sycp2
NM_011580	-1.60	0.00005	0.00043	Thbs1
NM_008086	-1.60	0.00005	0.00043	Gas1
NM_139300	-1.61	0.00005	0.00043	Mylk
NM_010111	-1.63	0.00005	0.00043	Efnb2
NM_009846	-1.64	0.00005	0.00043	Cd24a
NM_001167828	-1.65	0.00005	0.00043	Trim30d
NM_009721	-1.65	0.00005	0.00043	Atp1b1
NM_145467	-1.67	0.00005	0.00043	Itgbl1
NM_009062	-1.68	0.00005	0.00043	Rgs4
NM_138654	-1.71	0.00810	0.03519	5033411D12Rik
NM_008404	-1.72	0.00005	0.00043	Itgb2
NM_013468	-1.72	0.00005	0.00043	Ankrd1
NM_001195084	-1.72	0.00005	0.00043	Plscr2
NM_008176	-1.73	0.00005	0.00043	Cxcl1
NM_170778	-1.73	0.00005	0.00043	Dpyd
NM_001042660	-1.73	0.00005	0.00043	Smad7
NM_019564	-1.74	0.00130	0.00786	Htra1
NM_016873	-1.74	0.00005	0.00043	Wisp2
NM_008491	-1.75	0.00005	0.00043	Lcn2
NM_172118	-1.76	0.00005	0.00043	Myl9
NM_001033228	-1.80	0.00020	0.00154	Itga1
NM_007621	-1.80	0.00005	0.00043	Cbr2
NM_001013833	-1.82	0.00100	0.00627	Prkg1
NR_036452	-1.84	0.00005	0.00043	E230016K23Rik
NM_031393	-1.85	0.00745	0.03296	Sytl1
NM_011333	-1.85	0.00005	0.00043	Ccl2
NM_144556	-1.86	0.00815	0.03537	Lgi4
NM_001039048	-1.87	0.00015	0.00119	Trim63
NM_001199210	-1.87	0.00005	0.00043	Eva1c
NM_016689	-1.89	0.00015	0.00119	Aqp3
NM_025404	-1.90	0.00180	0.01036	Arl4d
NM_172393	-1.91	0.00005	0.00043	Aim1
NM_026821	-1.93	0.00005	0.00043	Lurap1l
NM_013743	-1.93	0.00005	0.00043	Pdk4
NR_040287	-1.95	0.00040	0.00284	A730020E08Rik
NR_026733	-1.95	0.00005	0.00043	3110039M20Rik
NM_001008424	-1.96	0.00005	0.00043	Cdsn
NM_198656	-1.97	0.00005	0.00043	Cdh26
NM_001038839	-2.01	0.00005	0.00043	P2rx7
NM_023048	-2.03	0.00005	0.00043	Asb4
NM_176933	-2.03	0.00005	0.00043	Dusp4

NR_045079	-2.09	0.00005	0.00043	3300005D01Rik
NM_172799	-2.14	0.00005	0.00043	Ttll6
NR_045905	-2.15	0.00880	0.03750	2700069I18Rik
NM_009610	-2.17	0.00230	0.01272	Actg2
NM_001172481	-2.19	0.00005	0.00043	Aspn
NM_030728	-2.23	0.00005	0.00043	9930013L23Rik
NM_010104	-2.30	0.00010	0.00082	Edn1
NM_007759	-2.35	0.00185	0.01061	Crabp2
NM_024406	-2.36	0.00005	0.00043	Fabp4
NM_011077	-2.37	0.00005	0.00043	Phex
NM_009701	-2.44	0.00060	0.00405	Aqp5
NM_013657	-2.47	0.00005	0.00043	Sema3c
NM_009655	-2.50	0.00005	0.00043	Alcam
NM_011526	-2.50	0.00005	0.00043	Tagln
NM_001037987	-2.57	0.00005	0.00043	Edil3
NM_001013764	-2.57	0.00030	0.00221	Ces1a
NM_008904	-2.67	0.00005	0.00043	Ppargc1a
NM_021355	-2.71	0.00005	0.00043	Fmod
NM_001042615	-2.72	0.00005	0.00043	Htra3
NM_020581	-2.78	0.00005	0.00043	Angptl4
NM_053106	-2.89	0.00005	0.00043	Lmod1
NM_010228	-2.90	0.00005	0.00043	Flt1
NM_011921	-2.95	0.00005	0.00043	Aldh1a7
NM_080639	-2.99	0.00005	0.00043	Timp4
NM_023608	-3.00	0.00005	0.00043	Gdpd2
NM_008555	-3.08	0.01035	0.04274	Masp1
NM_008760	-3.17	0.00005	0.00043	Ogn
NM_011338	-3.18	0.00005	0.00043	Ccl9
NM_008862	-3.20	0.00005	0.00043	Pkia
NM_026271	-3.26	0.00005	0.00043	Fibin
NM_023850	-3.68	0.00430	0.02134	Chst1
NM_001039934	-3.85	0.00005	0.00043	Map2
NM_011891	-3.88	0.00005	0.00043	Sgcd
NM_177346	-4.55	0.00005	0.00043	Gpr149

Hedgehog vs Esrrb+Hedgehog

gene	log2(fold_change)	p_value	q_value	Gene Symbol
NM_001110009	#NAME?	0.00385	0.04057	Apoc1
NM_009197	5.71	0.00005	0.00106	Slc16a2
NM_009244	5.11	0.00145	0.01869	Serpina1b
NM_029509	4.76	0.00070	0.01027	Gbp8
NM_001252569	4.17	0.00005	0.00106	Serpina1a
NM_001145807	3.77	0.00005	0.00106	Brinp3
NM_176973	3.52	0.00060	0.00905	Podxl2
NM_001198811	3.37	0.00005	0.00106	Frem1
NM_001256005	3.24	0.00005	0.00106	Gbp4
NM_152803	3.08	0.00010	0.00196	Hpse
NM_009144	3.07	0.00005	0.00106	Sfrp2
NM_010266	3.03	0.00005	0.00106	Gda
NM_001024139	3.00	0.00005	0.00106	Adamts15
NM_001037909	2.97	0.00005	0.00106	C130026I21Rik
NM_009285	2.92	0.00005	0.00106	Stc1
NM_033616	2.83	0.00005	0.00106	Csprs
NM_029472	2.79	0.00465	0.04687	Gstt4
NM_153131	2.71	0.00005	0.00106	Unc5a
NM_007730	2.68	0.00005	0.00106	Col12a1
NM_008270	2.51	0.00005	0.00106	Hoxb9
NM_008821	2.48	0.00035	0.00578	Pet2
NM_007957	2.46	0.00435	0.04449	Esx1
NM_001164566	2.45	0.00005	0.00106	Spats2l
NM_213615	2.43	0.00005	0.00106	A530032D15Rik

NM_001285917	2.33	0.00005	0.00106	Dapk1
NM_027406	2.33	0.00155	0.01970	Aldh1l1
NM_030194	2.33	0.00015	0.00282	Sp110
NM_010050	2.25	0.00005	0.00106	Dio2
NM_183201	2.18	0.00005	0.00106	Slfn5
NR_027817	2.17	0.00050	0.00781	1500011B03Rik
NM_025658	2.15	0.00005	0.00106	Ms4a4d
NM_001104547	2.14	0.00480	0.04811	Vmn2r96
NM_001081746	2.12	0.00005	0.00106	Gm7609
NM_026142	2.07	0.00005	0.00106	3632451O06Rik
NM_020509	2.03	0.00005	0.00106	Retnla
NM_001146180	1.98	0.00005	0.00106	Mtss1
NM_022018	1.97	0.00005	0.00106	Fam129a
NM_025492	1.93	0.00005	0.00106	1700020L24Rik
NM_001079869	1.89	0.00025	0.00433	Hoxb3
NM_080435	1.89	0.00010	0.00196	Adcy4
NM_008709	1.82	0.00005	0.00106	Mycn
NM_022312	1.79	0.00005	0.00106	Tnr
NM_175332	1.75	0.00400	0.04176	E130012A19Rik
NM_008744	1.72	0.00005	0.00106	Ntn1
NM_001039646	1.72	0.00005	0.00106	Gbp10
NM_013554	1.70	0.00070	0.01027	Hoxd10
NM_001164107	1.69	0.00065	0.00969	Ripk3
NM_001033249	1.69	0.00230	0.02699	Zfp583
NM_027852	1.65	0.00005	0.00106	Rarres2
NM_001128606	1.62	0.00005	0.00106	Epb4.1
NM_080453	1.62	0.00005	0.00106	Mmp28
NM_001098170	1.53	0.00005	0.00106	Pcdh10
NM_001039209	1.52	0.00005	0.00106	Gm13152
NM_001177752	1.48	0.00005	0.00106	Pfkfb3
NM_001122768	1.47	0.00005	0.00106	Lrrc8d
NM_001039647	1.46	0.00005	0.00106	Gbp11
NM_001122736	1.46	0.00005	0.00106	Igf2
NM_001039056	1.43	0.00005	0.00106	Kcnj15
NR_040297	1.41	0.00005	0.00106	Gm19757
NM_001009978	1.40	0.00075	0.01085	Pde1a
NM_001039239	1.38	0.00005	0.00106	Zfp808
NM_011909	1.35	0.00380	0.04017	Usp18
NM_177839	1.34	0.00035	0.00578	Tnn
NM_027828	1.30	0.00005	0.00106	Fam110c
NM_011315	1.30	0.00005	0.00106	Saa3
NM_013555	1.29	0.00005	0.00106	Hoxd9
NM_001162365	1.29	0.00050	0.00781	Ptk2b
NM_033270	1.28	0.00125	0.01650	E2f6
NM_001111268	1.26	0.00005	0.00106	Grik2
NM_173781	1.26	0.00005	0.00106	Rab6b
NM_001163476	1.21	0.00005	0.00106	Gins1
NM_054098	1.18	0.00005	0.00106	Steap4
NM_172563	1.18	0.00005	0.00106	Hlf
NM_015790	1.18	0.00050	0.00781	Icosl
NM_011623	1.17	0.00005	0.00106	Top2a
NM_020557	1.15	0.00005	0.00106	Cmpk2
NM_021342	1.14	0.00005	0.00106	Kcne4
NM_178609	1.14	0.00005	0.00106	E2f7
NM_001014976	1.13	0.00005	0.00106	Espl1
NM_011121	1.13	0.00005	0.00106	Plk1
NM_019419	1.11	0.00005	0.00106	Arl6ip1
NM_024184	1.11	0.00005	0.00106	Asf1b
NM_001025779	1.10	0.00005	0.00106	Cdc6
NM_008958	1.10	0.00005	0.00106	Ptch2
NM_029415	1.10	0.00005	0.00106	Slc10a6

NM_145588	1.10	0.00005	0.00106	Kif22
NM_134471	1.09	0.00005	0.00106	Kif2c
NM_009773	1.09	0.00005	0.00106	Bub1b
NM_172453	1.08	0.00005	0.00106	Pif1
NM_144786	1.07	0.00005	0.00106	Ggt7
NM_027290	1.07	0.00005	0.00106	Mcm10
NM_197959	1.07	0.00005	0.00106	Kif18b
NM_146208	1.06	0.00005	0.00106	Neil3
NM_016925	1.06	0.00005	0.00106	Fanca
NM_001163495	1.06	0.00005	0.00106	Arhgap19
NM_146238	1.06	0.00025	0.00433	Gemin8
NM_175554	1.06	0.00005	0.00106	Clspn
NM_008524	1.06	0.00005	0.00106	Lum
NM_172301	1.06	0.00005	0.00106	Ccnb1
NM_007708	1.06	0.00005	0.00106	Cit
NM_001003919	1.06	0.00005	0.00106	Ddx11
NM_175265	1.06	0.00005	0.00106	Bora
NM_001104550	1.05	0.00025	0.00433	Vmn2r98
NM_008276	1.05	0.00005	0.00106	Hoxd8
NM_001162506	1.05	0.00005	0.00106	Troap
NM_001081258	1.05	0.00005	0.00106	Kif14
NM_011858	1.05	0.00005	0.00106	Tenm4
NM_019759	1.05	0.00005	0.00106	Dpt
NM_028039	1.05	0.00005	0.00106	Esco2
NM_001168672	1.05	0.00005	0.00106	Gtse1
NM_172693	1.04	0.00005	0.00106	Galnt12
NM_029835	1.04	0.00005	0.00106	Ticrr
NM_008716	1.04	0.00005	0.00106	Notch3
NM_001080944	1.04	0.00025	0.00433	Atp8b4
NM_013673	1.04	0.00005	0.00106	Sp100
NM_001029838	1.04	0.00005	0.00106	Pknox2
NM_175314	1.03	0.00005	0.00106	Adamts9
NM_011497	1.03	0.00005	0.00106	Aurka
NM_011854	1.02	0.00485	0.04850	Oasl2
NM_001163793	1.02	0.00005	0.00106	C530008M17Rik
NM_001033484	1.02	0.00005	0.00106	Iqgap3
NM_001271729	1.02	0.00005	0.00106	Tk1
NM_001167743	1.02	0.00005	0.00106	Slfn8
NM_139232	1.01	0.00005	0.00106	Fgd4
NM_008651	1.01	0.00005	0.00106	Mybl1
NM_011496	1.01	0.00005	0.00106	Aurkb
NM_199223	1.01	0.00005	0.00106	Rtn4rl2
NM_008446	1.01	0.00005	0.00106	Kif4
NM_009185	1.01	0.00005	0.00106	Stil
NM_001081117	1.00	0.00005	0.00106	Mki67
NM_153544	1.00	0.00005	0.00106	BC030867
NM_001040397	-1.00	0.00005	0.00106	Filip1l
NM_019919	-1.01	0.00005	0.00106	Ltbp1
NM_011338	-1.04	0.00100	0.01377	Ccl9
NR_029439	-1.04	0.00040	0.00645	1700018A04Rik
NM_001177319	-1.05	0.00030	0.00510	Tfpi
NM_001164184	-1.06	0.00025	0.00433	Lsr
NM_175454	-1.08	0.00425	0.04382	Hid1
NM_019967	-1.09	0.00005	0.00106	Brinp1
NM_029920	-1.09	0.00005	0.00106	Mtus2
NM_007428	-1.09	0.00045	0.00714	Agt
NM_139197	-1.11	0.00020	0.00359	Gbgt1
NM_053106	-1.11	0.00010	0.00196	Lmod1
NM_172118	-1.11	0.00005	0.00106	Myl9
NM_025770	-1.15	0.00005	0.00106	Atg10
NM_008046	-1.15	0.00005	0.00106	Fst

NM_029631	-1.17	0.00005	0.00106	Abhd14b
NM_001164724	-1.18	0.00005	0.00106	Il33
NM_001083810	-1.19	0.00005	0.00106	Prr5l
NM_177819	-1.20	0.00005	0.00106	Fam135b
NM_001085376	-1.20	0.00005	0.00106	Pappa2
NM_020564	-1.22	0.00135	0.01757	Sult5a1
NM_198656	-1.22	0.00010	0.00196	Cdh26
NM_001008424	-1.22	0.00005	0.00106	Cdsn
NM_007470	-1.22	0.00005	0.00106	Apod
NM_013737	-1.23	0.00005	0.00106	Pla2g7
NM_177191	-1.23	0.00025	0.00433	Sycp2
NR_030484	-1.24	0.00005	0.00106	Mir703
NM_023608	-1.26	0.00005	0.00106	Gdpd2
NM_001286653	-1.27	0.00005	0.00106	Rcan2
NM_009131	-1.28	0.00005	0.00106	Clec11a
NM_024406	-1.28	0.00350	0.03760	Fabp4
NM_018779	-1.32	0.00005	0.00106	Pde3a
NM_001111073	-1.32	0.00005	0.00106	Fxyd5
NM_001081169	-1.33	0.00115	0.01541	Aspg
NM_011077	-1.35	0.00085	0.01209	Phex
NM_001081006	-1.36	0.00005	0.00106	Etl4
NM_053191	-1.37	0.00005	0.00106	Pi15
NM_011454	-1.39	0.00110	0.01492	Serpinb6b
NM_029881	-1.41	0.00005	0.00106	Tmem200a
NM_011921	-1.41	0.00080	0.01148	Aldh1a7
NM_145467	-1.43	0.00005	0.00106	Itgbl1
NM_019866	-1.46	0.00405	0.04221	Spib
NM_010228	-1.47	0.00070	0.01027	Flt1
NR_045079	-1.48	0.00005	0.00106	3300005D01Rik
NM_011766	-1.49	0.00005	0.00106	Zfpm2
NM_008235	-1.51	0.00005	0.00106	Hes1
NM_019503	-1.52	0.00005	0.00106	Fxyd1
NM_001177881	-1.52	0.00005	0.00106	Mfap3l
NM_144799	-1.53	0.00010	0.00196	Lmcd1
NM_010930	-1.55	0.00005	0.00106	Nov
NM_007621	-1.55	0.00005	0.00106	Cbr2
NM_028705	-1.57	0.00005	0.00106	Herc3
NM_001163015	-1.57	0.00005	0.00106	Gprasp2
NM_175271	-1.58	0.00005	0.00106	Lpar4
NM_001159965	-1.58	0.00005	0.00106	Ralgps2
NM_007729	-1.61	0.00005	0.00106	Col11a1
NM_173007	-1.62	0.00005	0.00106	Tspan12
NM_009846	-1.62	0.00005	0.00106	Cd24a
NM_001112725	-1.65	0.00115	0.01541	Aldh3a1
NM_023734	-1.65	0.00155	0.01970	Pi16
NM_009548	-1.66	0.00050	0.00781	Rnf112
NM_001285890	-1.66	0.00005	0.00106	Pde1b
NM_001243072	-1.70	0.00005	0.00106	Sema3a
NM_016917	-1.71	0.00005	0.00106	Slc40a1
NM_009701	-1.72	0.00335	0.03640	Aqp5
NM_138654	-1.85	0.00380	0.04017	5033411D12Rik
NM_001001334	-1.86	0.00155	0.01970	BC061194
NM_001194940	-1.88	0.00005	0.00106	Dlc1
NM_001167828	-1.90	0.00005	0.00106	Trim30d
NM_001033228	-1.94	0.00005	0.00106	Itga1
NM_008005	-1.95	0.00480	0.04811	Fgf18
NM_008760	-1.96	0.00005	0.00106	Ogn
NM_170778	-1.99	0.00005	0.00106	Dpyd
NM_001039094	-2.06	0.00005	0.00106	Negr1
NM_001037987	-2.09	0.00005	0.00106	Edil3
NM_001271705	-2.10	0.00005	0.00106	Pgf

NM_009655	-2.12	0.00005	0.00106	Alcam
NM_008407	-2.14	0.00155	0.01970	Itih3
NM_001039220	-2.15	0.00005	0.00106	AI429214
NM_001172481	-2.16	0.00005	0.00106	Aspn
NM_029568	-2.17	0.00005	0.00106	Mfap4
NM_001285956	-2.26	0.00005	0.00106	Podn
NM_001122595	-2.45	0.00090	0.01268	A630033H20Rik
NM_001284380	-2.50	0.00040	0.00645	Adra1b
NM_013657	-2.52	0.00005	0.00106	Sema3c
NM_008904	-2.73	0.00005	0.00106	Ppargc1a
NM_144556	-2.98	0.00010	0.00196	Lgi4
NM_001166493	-3.07	0.00025	0.00433	Rasgrp3
NM_026271	-3.12	0.00005	0.00106	Fibin
NM_021355	-3.27	0.00005	0.00106	Fmod
NM_023850	-3.41	0.00275	0.03117	Chst1
NM_001256382	-3.50	0.00005	0.00106	Rims2
NM_008862	-3.54	0.00005	0.00106	Pkia
NM_011891	-3.83	0.00005	0.00106	Sgcd
NM_001039934	-4.21	0.00005	0.00106	Map2
NM_177346	-4.25	0.00005	0.00106	Gpr149

Esrrb vs Esrrb+Hedgehog

gene	log2(fold_change)	p_value	q_value	Gene Symbol
NM_028473	inf	0.00005	0.00068	3110079015Rik
NM_028804	inf	0.00005	0.00068	Ccdc3
NM_029472	inf	0.00005	0.00068	Gstt4
NM_010296	8.87	0.00005	0.00068	Gli1
NM_020509	7.10	0.00005	0.00068	Retnla
NM_001044751	5.90	0.00005	0.00068	Hsd11b1
NM_025429	4.31	0.00005	0.00068	Serpinb1a
NM_008957	3.96	0.00005	0.00068	Ptch1
NM_008380	3.63	0.00005	0.00068	Inhba
NM_016867	3.61	0.00005	0.00068	Gipc2
NM_020557	3.48	0.00005	0.00068	Cmpk2
NM_009641	3.47	0.00005	0.00068	Angpt4
NM_001099276	3.30	0.00005	0.00068	Pik3c2b
NM_022657	3.30	0.00005	0.00068	Fgf23
NM_009117	3.22	0.00485	0.03874	Saa1
NM_007719	3.08	0.00110	0.01094	Ccr7
NM_016808	3.06	0.00005	0.00068	Usp2
NM_153137	2.97	0.00005	0.00068	Traf3ip3
NM_010517	2.94	0.00005	0.00068	Igfbp4
NM_015790	2.90	0.00015	0.00185	Icosl
NM_001177713	2.89	0.00005	0.00068	Cyp26b1
NM_011452	2.72	0.00090	0.00914	Serpinb9b
NM_010740	2.66	0.00005	0.00068	Cd93
NM_016854	2.65	0.00005	0.00068	Ppp1r3c
NM_010518	2.64	0.00005	0.00068	Igfbp5
NM_001033633	2.51	0.00005	0.00068	Slc2a13
NM_009382	2.47	0.00005	0.00068	Thy1
NM_007539	2.42	0.00005	0.00068	Bdkrb1
NM_138595	2.33	0.00115	0.01136	Gldc
NM_001145886	2.22	0.00005	0.00068	Tiam1
NR_030721	2.20	0.00005	0.00068	Foxd2os
NM_011623	2.19	0.00005	0.00068	Top2a
NM_172756	2.17	0.00005	0.00068	Ankle1
NM_172564	2.16	0.00005	0.00068	Tns4
NM_001081085	2.15	0.00005	0.00068	Sapcd2
NM_001167680	2.14	0.00005	0.00068	Rhbdf2
NM_177343	2.13	0.00005	0.00068	Camk1d
NM_001081363	2.12	0.00005	0.00068	Cenpf

NM_001159518	2.11	0.00005	0.00068	Igfbp7
NR_037955	2.11	0.00005	0.00068	1190002F15Rik
NM_001164355	2.08	0.00005	0.00068	Ska1
NM_001195025	2.07	0.00005	0.00068	Nuak2
NM_146208	2.05	0.00005	0.00068	Neil3
NM_001160012	2.04	0.00020	0.00239	Gjb3
NM_172671	2.04	0.00005	0.00068	Lgr4
NM_001168318	2.02	0.00005	0.00068	Scara5
NM_001013377	2.01	0.00020	0.00239	Arhgef39
NM_001033141	2.01	0.00410	0.03386	Ecscr
NM_139197	2.01	0.00590	0.04548	Gbgt1
NM_019971	2.00	0.00005	0.00068	Pdgfc
NM_001081258	2.00	0.00005	0.00068	Kif14
NR_030721	2.00	0.00005	0.00068	Foxd2os
NM_001177752	2.00	0.00005	0.00068	Pfkfb3
NM_001081117	1.99	0.00005	0.00068	Mki67
NM_145356	1.99	0.00005	0.00068	Zbtb7c
NM_009931	1.99	0.00005	0.00068	Col4a1
NM_009791	1.96	0.00005	0.00068	Aspm
NM_029835	1.95	0.00005	0.00068	Ticrr
NM_008185	1.93	0.00005	0.00068	Gstt1
NM_001162506	1.92	0.00005	0.00068	Troap
NM_176982	1.92	0.00005	0.00068	Fbxo48
NR_015469	1.90	0.00005	0.00068	2810442I21Rik
NM_007695	1.90	0.00005	0.00068	Chil1
NM_001014976	1.88	0.00005	0.00068	Espl1
NM_001177794	1.87	0.00005	0.00068	Sertad4
NM_009437	1.87	0.00045	0.00496	Tst
NM_001281819	1.85	0.00005	0.00068	Ace
NM_009373	1.82	0.00005	0.00068	Tgm2
NM_001003919	1.82	0.00005	0.00068	Ddx11
NM_010790	1.81	0.00005	0.00068	Melk
NM_029617	1.81	0.00005	0.00068	Casc5
NM_011121	1.81	0.00005	0.00068	Plk1
NM_016851	1.80	0.00005	0.00068	Irf6
NM_153805	1.80	0.00015	0.00185	Pkn3
NM_008489	1.78	0.00005	0.00068	Lbp
NM_001042421	1.78	0.00005	0.00068	Kntc1
NM_198423	1.77	0.00005	0.00068	Bahcc1
NM_053173	1.77	0.00005	0.00068	Kifc5b
NM_001162533	1.77	0.00105	0.01049	Sh3d21
NM_009013	1.76	0.00005	0.00068	Rad51ap1
NM_134471	1.76	0.00005	0.00068	Kif2c
NM_026560	1.76	0.00005	0.00068	Cdca8
NM_013538	1.75	0.00005	0.00068	Cdca3
NR_001592	1.74	0.00005	0.00068	H19
NM_172301	1.73	0.00005	0.00068	Ccnb1
NM_028039	1.73	0.00005	0.00068	Esco2
NM_009860	1.73	0.00005	0.00068	Cdc25c
NM_001199696	1.73	0.00005	0.00068	Bai2
NM_001033464	1.73	0.00005	0.00068	Efcab4b
NM_019753	1.72	0.00005	0.00068	Cdh17
NM_008651	1.72	0.00005	0.00068	Mybl1
NM_053191	1.72	0.00005	0.00068	Pi15
NM_031168	1.72	0.00005	0.00068	Il6
NM_001110265	1.71	0.00005	0.00068	Ttk
NM_027975	1.68	0.00005	0.00068	Fam83d
NM_001013368	1.68	0.00005	0.00068	E2f8
NR_015556	1.67	0.00005	0.00068	2610035D17Rik
NM_173762	1.67	0.00005	0.00068	Cenpe
NM_023223	1.67	0.00005	0.00068	Cdc20

NM_080640	1.67	0.00010	0.00129	Baalc
NM_009252	1.67	0.00005	0.00068	Serpina3n
NM_010620	1.67	0.00005	0.00068	Kif15
NM_001113179	1.67	0.00005	0.00068	Bub1
NM_144553	1.66	0.00005	0.00068	Dlgap5
NM_001033331	1.65	0.00005	0.00068	Gas2l3
NM_017407	1.65	0.00005	0.00068	Spag5
NM_011496	1.65	0.00005	0.00068	Aurkb
NM_027182	1.64	0.00005	0.00068	Trip13
NM_028222	1.64	0.00005	0.00068	Cdkn3
NM_001271729	1.64	0.00005	0.00068	Tk1
NM_013552	1.64	0.00005	0.00068	Hmmr
NM_001113460	1.62	0.00005	0.00068	Tec
NM_011497	1.62	0.00005	0.00068	Aurka
NM_008021	1.61	0.00005	0.00068	Foxm1
NM_001141975	1.61	0.00005	0.00068	Tpx2
NM_009932	1.61	0.00005	0.00068	Col4a2
NM_181589	1.60	0.00005	0.00068	Ckap2l
NM_146171	1.60	0.00005	0.00068	Ncapd2
NM_001163495	1.60	0.00005	0.00068	Arhgap19
NM_001285997	1.60	0.00005	0.00068	Prc1
NM_024245	1.60	0.00005	0.00068	Kif23
NM_007708	1.60	0.00005	0.00068	Cit
NM_008055	1.60	0.00005	0.00068	Fzd4
NM_023209	1.60	0.00005	0.00068	Pbk
NM_001172092	1.59	0.00005	0.00068	Depdc1a
NM_178395	1.59	0.00045	0.00496	Zdhhc2
NM_181848	1.59	0.00005	0.00068	Optn
NM_009828	1.59	0.00005	0.00068	Ccna2
NM_009253	1.59	0.00005	0.00068	Serpina3m
NM_026785	1.58	0.00005	0.00068	Ube2c
NM_027290	1.58	0.00005	0.00068	Mcm10
NM_001163471	1.58	0.00005	0.00068	Hectd2
NM_001168672	1.58	0.00005	0.00068	Gtse1
NM_058214	1.57	0.00005	0.00068	Recql4
NM_144526	1.57	0.00005	0.00068	Fam64a
NM_029249	1.57	0.00005	0.00068	Parpbp
NM_001195298	1.56	0.00005	0.00068	Kifc1
NM_010615	1.56	0.00005	0.00068	Kif11
NM_175554	1.56	0.00005	0.00068	Clspn
NM_197959	1.56	0.00005	0.00068	Kif18b
NM_134041	1.56	0.00005	0.00068	4930427A07Rik
NM_001199123	1.56	0.00005	0.00068	Spc25
NM_010892	1.55	0.00005	0.00068	Nek2
NM_028870	1.55	0.00005	0.00068	Cltb
NM_007681	1.55	0.00005	0.00068	Cenpa
NM_018827	1.55	0.00005	0.00068	Crlf1
NR_024720	1.55	0.00005	0.00068	2700099C18Rik
NM_001164362	1.55	0.00005	0.00068	Cep55
NM_001080158	1.54	0.00005	0.00068	Cenpm
NM_001033484	1.54	0.00005	0.00068	Iqgap3
NM_001081406	1.54	0.00165	0.01543	Lrr1
NM_139001	1.54	0.00005	0.00068	Cspg4
NM_001040435	1.53	0.00005	0.00068	Tacc3
NM_198654	1.53	0.00005	0.00068	Nsl1
NM_009773	1.53	0.00005	0.00068	Bub1b
NM_198605	1.53	0.00005	0.00068	Ska3
NM_009185	1.53	0.00005	0.00068	Stil
NM_001110162	1.53	0.00005	0.00068	Cdca2
NM_001012273	1.52	0.00005	0.00068	Birc5
NM_153762	1.52	0.00005	0.00068	Rnf26

NM_026412	1.51	0.00005	0.00068	Knstrn
NM_026778	1.51	0.00005	0.00068	Cthrc1
NM_008446	1.51	0.00005	0.00068	Kif4
NM_139303	1.51	0.00005	0.00068	Kif18a
NM_021886	1.51	0.00005	0.00068	Cenph
NM_175563	1.51	0.00005	0.00068	Prr11
NM_023294	1.51	0.00005	0.00068	Ndc80
NM_026507	1.50	0.00005	0.00068	Zwilch
NM_025979	1.49	0.00005	0.00068	Mastl
NM_001193305	1.49	0.00005	0.00068	Mical2
NM_001085549	1.49	0.00005	0.00068	Trabd2b
NM_016719	1.49	0.00005	0.00068	Grb14
NM_017370	1.49	0.00005	0.00068	Hp
NM_019438	1.49	0.00005	0.00068	Ncapg
NM_145924	1.49	0.00005	0.00068	Cenpi
NM_145588	1.48	0.00005	0.00068	Kif22
NM_178609	1.48	0.00005	0.00068	E2f7
NM_010050	1.48	0.00005	0.00068	Dio2
NM_026282	1.47	0.00005	0.00068	Spc24
NM_001172216	1.47	0.00105	0.01049	Fam221a
NM_028666	1.47	0.00005	0.00068	Fam110a
NM_009764	1.47	0.00005	0.00068	Brca1
NM_001039556	1.47	0.00005	0.00068	Rad54b
NM_016925	1.47	0.00005	0.00068	Fanca
NM_026410	1.46	0.00005	0.00068	Cdca5
NM_011369	1.46	0.00005	0.00068	Shcbp1
NM_001127259	1.45	0.00005	0.00068	Trp63
NM_028959	1.45	0.00040	0.00450	Cep72
NM_023284	1.45	0.00005	0.00068	Nuf2
NM_183046	1.45	0.00005	0.00068	Kif20b
NM_172453	1.44	0.00005	0.00068	Pif1
NM_021790	1.44	0.00600	0.04616	Cenpk
NM_134072	1.44	0.00005	0.00068	Akr1c14
NM_001253808	1.44	0.00005	0.00068	Racgap1
NM_028481	1.43	0.00005	0.00068	Ccdc18
NM_001163359	1.43	0.00005	0.00068	Fignl1
NM_027354	1.43	0.00005	0.00068	Poc1a
NM_001024474	1.42	0.00020	0.00239	Diras2
NM_007993	1.42	0.00005	0.00068	Fbn1
NM_181815	1.42	0.00005	0.00068	Cep128
NM_026515	1.41	0.00010	0.00129	2810417H13Rik
NM_018779	1.41	0.00005	0.00068	Pde3a
NM_028232	1.41	0.00005	0.00068	Sgol1
NM_144818	1.41	0.00005	0.00068	Ncaph
NM_001033244	1.40	0.00005	0.00068	Fancd2
NM_001033455	1.40	0.00075	0.00777	Ccdc27
NM_024184	1.40	0.00005	0.00068	Asf1b
NM_016692	1.40	0.00005	0.00068	Incenp
NM_010637	1.38	0.00005	0.00068	Klf4
NM_146235	1.38	0.00005	0.00068	Ercc6l
NM_001177625	1.38	0.00005	0.00068	Ect2
NM_134000	1.37	0.00225	0.02018	Traf3ip2
NM_008652	1.36	0.00005	0.00068	Mybl2
NM_008276	1.36	0.00005	0.00068	Hoxd8
NM_183089	1.36	0.00005	0.00068	Dscc1
NM_019444	1.36	0.00005	0.00068	Ramp2
NM_030172	1.36	0.00075	0.00777	Efcab11
NM_009320	1.36	0.00005	0.00068	Slc6a6
NM_080467	1.35	0.00005	0.00068	Atp6v0a4
NM_175265	1.35	0.00005	0.00068	Bora
NM_011132	1.35	0.00005	0.00068	Pole

NM_026613	1.35	0.00005	0.00068	Ccdc34
NM_027930	1.34	0.00005	0.00068	Mtfr2
NM_028390	1.34	0.00005	0.00068	Anln
NM_013571	1.34	0.00005	0.00068	Ksr1
NM_016917	1.33	0.00005	0.00068	Slc40a1
NM_181416	1.33	0.00005	0.00068	Arhgap11a
NM_001159369	1.33	0.00005	0.00068	Polq
NM_009802	1.32	0.00060	0.00636	Car6
NM_172578	1.32	0.00005	0.00068	Mis18bp1
NM_023058	1.31	0.00005	0.00068	Pkmyt1
NM_172145	1.31	0.00005	0.00068	Eva1b
NM_001163763	1.31	0.00005	0.00068	Tcf19
NM_001252055	1.31	0.00005	0.00068	Ly6c1
NM_001004140	1.31	0.00005	0.00068	Ckap2
NM_001025779	1.31	0.00005	0.00068	Cdc6
NM_001145827	1.31	0.00005	0.00068	Stk40
NM_001166406	1.31	0.00005	0.00068	Kif20a
NM_172616	1.31	0.00005	0.00068	C330027C09Rik
NM_001111078	1.30	0.00005	0.00068	Uhrf1
NM_007634	1.30	0.00005	0.00068	Ccnf
NM_080850	1.29	0.00010	0.00129	Pask
NM_008209	1.29	0.00005	0.00068	Mr1
NM_001040631	1.29	0.00005	0.00068	C1qtnf5
NM_001278115	1.29	0.00010	0.00129	Ube2t
NM_025566	1.29	0.00025	0.00294	Tnfaip8l1
NM_001167730	1.28	0.00005	0.00068	Rad18
NM_001146081	1.28	0.00005	0.00068	Fancb
NM_001177867	1.27	0.00005	0.00068	Sgol2
NM_008737	1.27	0.00005	0.00068	Nrp1
NM_177331	1.27	0.00005	0.00068	Gen1
NM_145409	1.26	0.00005	0.00068	Chtf18
NM_001037865	1.26	0.00005	0.00068	Col28a1
NM_001039150	1.26	0.00005	0.00068	Cd44
NM_009061	1.26	0.00005	0.00068	Rgs2
NM_027263	1.26	0.00095	0.00960	Apitd1
NM_001111075	1.25	0.00005	0.00068	Cdc25b
NR_033388	1.25	0.00005	0.00068	Gm3002
NM_001039543	1.25	0.00005	0.00068	Mlf1
NM_019641	1.24	0.00005	0.00068	Stmn1
NM_001081238	1.24	0.00185	0.01705	Palb2
NM_178683	1.24	0.00010	0.00129	Depdc1b
NM_008017	1.23	0.00005	0.00068	Smc2
NM_007525	1.23	0.00005	0.00068	Bard1
NM_026743	1.23	0.00005	0.00068	Tspan11
NM_025676	1.23	0.00005	0.00068	Mcm8
NM_013807	1.23	0.00005	0.00068	Plk3
NM_025928	1.23	0.00005	0.00068	Pmf1
NM_001081125	1.23	0.00005	0.00068	Gli2
NM_001131054	1.22	0.00005	0.00068	Pttg1
NM_001083188	1.22	0.00005	0.00068	Lig1
NM_001037134	1.22	0.00060	0.00636	Ccne2
NM_027435	1.22	0.00005	0.00068	Atad2
NM_011234	1.22	0.00005	0.00068	Rad51
NM_001163476	1.22	0.00005	0.00068	Gins1
NM_172598	1.21	0.00005	0.00068	Wdhd1
NM_013822	1.21	0.00005	0.00068	Jag1
NM_153544	1.21	0.00010	0.00129	BC030867
NM_011634	1.21	0.00005	0.00068	Traip
NM_012012	1.21	0.00005	0.00068	Exo1
NM_010657	1.21	0.00005	0.00068	Hivep3
NM_001113350	1.20	0.00050	0.00543	Xkr5

NM_007472	1.20	0.00005	0.00068	Aqp1
NM_025415	1.20	0.00005	0.00068	Cks2
NM_001159646	1.20	0.00005	0.00068	Dut
NM_001271768	1.20	0.00005	0.00068	Bhlhe41
NM_019499	1.20	0.00005	0.00068	Mad2l1
NM_011427	1.20	0.00005	0.00068	Snai1
NM_008566	1.19	0.00005	0.00068	Mcm5
NM_001113211	1.19	0.00005	0.00068	Tmem194
NM_020265	1.19	0.00005	0.00068	Dkk2
NM_009104	1.19	0.00005	0.00068	Rrm2
NM_008921	1.18	0.00005	0.00068	Prim1
NM_001159930	1.18	0.00005	0.00068	Cenpl
NM_177819	1.18	0.00005	0.00068	Fam135b
NM_008252	1.18	0.00005	0.00068	Hmgb2
NM_008871	1.18	0.00005	0.00068	Serpine1
NM_001042527	1.18	0.00005	0.00068	Blm
NM_145946	1.18	0.00070	0.00730	Fanci
NM_027225	1.17	0.00005	0.00068	Cobll1
NM_178309	1.16	0.00005	0.00068	Brip1
NM_080553	1.16	0.00005	0.00068	Itpr3
NM_029766	1.16	0.00005	0.00068	Dtl
NM_177372	1.16	0.00020	0.00239	Dna2
NM_013555	1.16	0.00005	0.00068	Hoxd9
NM_001159748	1.16	0.00005	0.00068	Serpinb8
NM_001080995	1.16	0.00005	0.00068	4632434I11Rik
NM_028006	1.15	0.00005	0.00068	Tube1
NM_033270	1.15	0.00420	0.03436	E2f6
NM_001081445	1.14	0.00005	0.00068	Ncam1
NM_025642	1.14	0.00005	0.00068	Mis18a
NM_025590	1.14	0.00005	0.00068	Acot11
NM_010239	1.14	0.00005	0.00068	Fth1
NM_010049	1.13	0.00005	0.00068	Dhfr
NM_009516	1.13	0.00005	0.00068	Wee1
NM_001136082	1.13	0.00005	0.00068	Timeless
NM_001276248	1.13	0.00005	0.00068	Cp
NM_011495	1.13	0.00005	0.00068	Plk4
NM_198622	1.13	0.00005	0.00068	H1fx
NM_031863	1.13	0.00005	0.00068	Cenpq
NM_134117	1.13	0.00005	0.00068	Pkdcc
NM_173739	1.13	0.00005	0.00068	Galnt18
NM_028128	1.12	0.00005	0.00068	Rfc5
NM_001081364	1.12	0.00005	0.00068	Arhgap21
NM_013691	1.12	0.00005	0.00068	Thbs3
NM_010468	1.12	0.00205	0.01862	Hoxd3
NM_011264	1.11	0.00005	0.00068	Rev3l
NM_029509	1.11	0.00005	0.00068	Gbp8
NM_001122768	1.11	0.00005	0.00068	Lrrc8d
NM_013733	1.11	0.00005	0.00068	Chaf1a
NM_025995	1.11	0.00005	0.00068	Fbxo5
NM_011638	1.10	0.00005	0.00068	Tfrc
NM_133762	1.10	0.00005	0.00068	Ncapg2
NM_001114386	1.10	0.00005	0.00068	Nedd4l
NM_172505	1.10	0.00005	0.00068	A730008H23Rik
NM_001190717	1.10	0.00005	0.00068	Dbf4
NM_019521	1.10	0.00005	0.00068	Gas6
NR_030682	1.09	0.00005	0.00068	2810410L24Rik
NM_201364	1.09	0.00005	0.00068	BC055324
NM_021288	1.09	0.00005	0.00068	Tyms
NM_146186	1.09	0.00005	0.00068	Wdr62
NR_033225	1.08	0.00005	0.00068	Gm13375
NM_028131	1.08	0.00010	0.00129	Cenpn

NM_028083	1.08	0.00005	0.00068	Chaf1b
NM_133687	1.08	0.00005	0.00068	Cxxc5
NM_001163356	1.08	0.00125	0.01222	Fam212b
NM_001029856	1.07	0.00005	0.00068	Atad5
NM_027871	1.07	0.00005	0.00068	Arhgef3
NM_007659	1.07	0.00005	0.00068	Cdk1
NM_001032413	1.07	0.00005	0.00068	Pear1
NM_146061	1.06	0.00005	0.00068	Prr5
NM_001164433	1.06	0.00005	0.00068	Mical1
NM_001085376	1.06	0.00005	0.00068	Pappa2
NM_001166629	1.05	0.00160	0.01505	Dynlt1a
NM_008458	1.05	0.00345	0.02925	Serpina3c
NM_001038642	1.05	0.00005	0.00068	Ets1
NM_001285945	1.05	0.00005	0.00068	Zranb3
NM_146116	1.05	0.00005	0.00068	Tubb4b
NM_001163480	1.05	0.00005	0.00068	Neurl1a
NM_016861	1.04	0.00005	0.00068	Pdlim1
NM_001081091	1.04	0.00005	0.00068	Cep152
NM_010220	1.04	0.00005	0.00068	Fkbp5
NM_013529	1.04	0.00005	0.00068	Gfpt2
NM_020567	1.04	0.00005	0.00068	Gmnn
NM_001242396	1.04	0.00005	0.00068	Jmjd1c
NM_010791	1.03	0.00045	0.00496	Meox1
NM_019670	1.03	0.00005	0.00068	Diap3
NM_001136071	1.03	0.00005	0.00068	Lsp1
NM_029482	1.03	0.00005	0.00068	4930579G24Rik
NM_001025365	1.02	0.00005	0.00068	Miip
NM_012018	1.02	0.00005	0.00068	Cep110
NM_008563	1.02	0.00005	0.00068	Mcm3
NM_010353	1.01	0.00005	0.00068	Gsg2
NM_009542	1.01	0.00005	0.00068	Zfp101
NM_178252	1.01	0.00005	0.00068	Arhgap33
NM_178856	1.01	0.00070	0.00730	Gins2
NM_027948	1.01	0.00135	0.01303	1700003E16Rik
NM_001145968	-1.01	0.00265	0.02329	Rwdd2a
NM_001159394	-1.01	0.00005	0.00068	Nfkbiz
NM_001198894	-1.01	0.00520	0.04107	Gpr56
NM_013599	-1.01	0.00005	0.00068	Mmp9
NM_009834	-1.02	0.00005	0.00068	Ccrn4l
NM_177906	-1.02	0.00005	0.00068	Opcml
NM_013468	-1.02	0.00005	0.00068	Ankrd1
NM_001284507	-1.03	0.00005	0.00068	Crabp1
NM_001025577	-1.03	0.00005	0.00068	Maf
NM_153529	-1.05	0.00005	0.00068	Nrn1
NM_054056	-1.05	0.00005	0.00068	Pawr
NM_025685	-1.05	0.00005	0.00068	Col27a1
NM_010132	-1.05	0.00005	0.00068	Emx2
NM_001135100	-1.06	0.00035	0.00397	Il34
NM_177068	-1.06	0.00005	0.00068	Olfml2b
NM_172856	-1.06	0.00005	0.00068	Cers6
NM_001177810	-1.06	0.00005	0.00068	Rapgef3
NM_016919	-1.07	0.00005	0.00068	Col5a3
NM_009675	-1.07	0.00005	0.00068	Aoc3
NM_007950	-1.07	0.00015	0.00185	Ereg
NM_001271531	-1.07	0.00005	0.00068	Insig2
NM_027950	-1.08	0.00005	0.00068	Osgin1
NM_001104550	-1.08	0.00005	0.00068	Vmn2r98
NM_026821	-1.08	0.00005	0.00068	Lurap1l
NM_008046	-1.09	0.00005	0.00068	Fst
NM_009154	-1.10	0.00005	0.00068	Sema5a
NM_001039239	-1.10	0.00005	0.00068	Zfp808

NM_001109758	-1.11	0.00445	0.03612	Bcan
NM_001252578	-1.11	0.00005	0.00068	Sulf2
NM_001164316	-1.12	0.00005	0.00068	Ccser1
NM_023617	-1.12	0.00005	0.00068	Aox3
NM_172815	-1.13	0.00005	0.00068	Rspo2
NM_010924	-1.13	0.00005	0.00068	Nnmt
NM_001040085	-1.13	0.00005	0.00068	Sytl2
NM_012030	-1.13	0.00005	0.00068	Slc9a3r1
NM_013834	-1.14	0.00005	0.00068	Sfrp1
NM_008630	-1.14	0.00005	0.00068	Mt2
NM_026189	-1.16	0.00005	0.00068	Eepd1
NM_145144	-1.16	0.00005	0.00068	Aif1l
NM_021381	-1.17	0.00005	0.00068	Prokr1
NM_008349	-1.18	0.00005	0.00068	Il10rb
NM_133764	-1.18	0.00005	0.00068	Atp6v0e2
NM_001042779	-1.18	0.00005	0.00068	Sema3b
NM_172648	-1.19	0.00035	0.00397	Ifi205
NM_023844	-1.19	0.00005	0.00068	Jam2
NM_001172481	-1.19	0.00005	0.00068	Aspn
NR_015602	-1.20	0.00005	0.00068	F730043M19Rik
NM_001079844	-1.20	0.00005	0.00068	Gpc6
NM_008872	-1.20	0.00005	0.00068	Plat
NM_011581	-1.21	0.00005	0.00068	Thbs2
NM_133955	-1.21	0.00005	0.00068	Rhou
NM_054041	-1.22	0.00005	0.00068	Antxr1
NM_001271483	-1.22	0.00005	0.00068	Clip4
NM_011610	-1.22	0.00005	0.00068	Tnfrsf1b
NM_028426	-1.22	0.00255	0.02254	3110007F17Rik
NM_008057	-1.22	0.00005	0.00068	Fzd7
NM_008973	-1.24	0.00005	0.00068	Ptn
NM_176922	-1.24	0.00005	0.00068	Itga11
NM_023386	-1.24	0.00550	0.04308	Rtp4
NM_001081977	-1.25	0.00005	0.00068	Rnf144a
NM_010151	-1.26	0.00005	0.00068	Nr2f1
NM_008321	-1.26	0.00005	0.00068	Id3
NM_178591	-1.26	0.00005	0.00068	Nrg1
NM_001242423	-1.27	0.00005	0.00068	Fam105a
NM_031166	-1.27	0.00445	0.03612	Id4
NM_013654	-1.27	0.00005	0.00068	Ccl7
NM_001204129	-1.27	0.00005	0.00068	C1qtnf1
NR_045079	-1.27	0.00015	0.00185	3300005D01Rik
NM_010750	-1.27	0.00005	0.00068	Mab21l1
NM_010516	-1.28	0.00005	0.00068	Cyr61
NM_001081433	-1.28	0.00005	0.00068	Ankrd44
NM_198656	-1.28	0.00045	0.00496	Cdh26
NM_010234	-1.28	0.00530	0.04167	Fos
NM_001037987	-1.28	0.00005	0.00068	Edil3
NM_008491	-1.29	0.00005	0.00068	Lcn2
NM_010111	-1.29	0.00005	0.00068	Efnb2
NM_009285	-1.30	0.00005	0.00068	Stc1
NM_011198	-1.30	0.00005	0.00068	Ptgs2
NM_008176	-1.30	0.00005	0.00068	Cxcl1
NM_001276489	-1.30	0.00005	0.00068	Ism1
NM_016873	-1.31	0.00005	0.00068	Wisp2
NM_173008	-1.31	0.00005	0.00068	Ssc5d
NM_011839	-1.32	0.00430	0.03502	Mab21l2
NM_008695	-1.35	0.00005	0.00068	Nid2
NM_013495	-1.36	0.00005	0.00068	Cpt1a
NM_001039048	-1.36	0.00250	0.02214	Trim63
NM_001081307	-1.37	0.00005	0.00068	Ppp1r12b
NM_023455	-1.37	0.00005	0.00068	Nat8

NR_026733	-1.38	0.00005	0.00068	3110039M20Rik
NM_199223	-1.39	0.00005	0.00068	Rtn4rl2
NM_182808	-1.40	0.00015	0.00185	Fam19a1
NM_001190911	-1.40	0.00015	0.00185	Kirrel3
NM_009144	-1.40	0.00005	0.00068	Sfrp2
NM_007400	-1.41	0.00005	0.00068	Adam12
NM_007901	-1.42	0.00005	0.00068	S1pr1
NM_018781	-1.43	0.00005	0.00068	Egr3
NR_045098	-1.43	0.00005	0.00068	Gm2115
NM_011330	-1.45	0.00125	0.01222	Ccl11
NM_001103177	-1.46	0.00005	0.00068	Ablim1
NM_009676	-1.46	0.00005	0.00068	Aox1
NM_001042660	-1.47	0.00005	0.00068	Smad7
NM_008750	-1.48	0.00005	0.00068	Nxn
NM_001172424	-1.49	0.00005	0.00068	Dhrs3
NM_001081756	-1.49	0.00005	0.00068	Nckap5
NM_001113325	-1.49	0.00005	0.00068	Gria1
NM_139300	-1.49	0.00005	0.00068	Mylk
NM_053106	-1.50	0.00005	0.00068	Lmod1
NM_001195084	-1.51	0.00005	0.00068	Plscr2
NM_011526	-1.51	0.00055	0.00590	Tagln
NM_001081127	-1.54	0.00005	0.00068	Adamts14
NM_025427	-1.54	0.00005	0.00068	Rgcc
NR_036452	-1.54	0.00045	0.00496	E230016K23Rik
NM_009721	-1.55	0.00005	0.00068	Atp1b1
NM_011921	-1.56	0.00055	0.00590	Aldh1a7
NM_011315	-1.56	0.00005	0.00068	Saa3
NM_011352	-1.58	0.00015	0.00185	Sema7a
NM_026436	-1.59	0.00005	0.00068	Tmem86a
NM_019564	-1.59	0.00405	0.03361	Htra1
NM_008086	-1.59	0.00005	0.00068	Gas1
NM_008404	-1.62	0.00005	0.00068	Itgb2
NM_001038839	-1.62	0.00005	0.00068	P2rx7
NM_023048	-1.63	0.00005	0.00068	Asb4
NM_027828	-1.64	0.00005	0.00068	Fam110c
NM_007413	-1.67	0.00005	0.00068	Adora2b
NM_009062	-1.68	0.00005	0.00068	Rgs4
NM_007562	-1.69	0.00005	0.00068	Bnc1
NM_029600	-1.69	0.00005	0.00068	Abcc3
NM_007498	-1.70	0.00005	0.00068	Atf3
NM_013467	-1.71	0.00005	0.00068	Aldh1a1
NM_009899	-1.72	0.00005	0.00068	Clca1
NM_009141	-1.73	0.00005	0.00068	Cxcl5
NM_001025602	-1.76	0.00005	0.00068	Il1rl1
NM_013743	-1.77	0.00005	0.00068	Pdk4
NM_011580	-1.79	0.00005	0.00068	Thbs1
NM_011118	-1.81	0.00005	0.00068	Prl2c3
NM_010104	-1.85	0.00215	0.01944	Edn1
NM_016689	-1.86	0.00035	0.00397	Aqp3
NM_026416	-1.87	0.00005	0.00068	S100a16
NM_176933	-1.87	0.00005	0.00068	Dusp4
NM_011333	-1.89	0.00005	0.00068	Ccl2
NM_172799	-1.93	0.00020	0.00239	Ttll6
NM_172393	-2.00	0.00005	0.00068	Aim1
NM_001029836	-2.03	0.00005	0.00068	Npnt
NM_010228	-2.16	0.00005	0.00068	Flt1
NM_178738	-2.18	0.00005	0.00068	Prss35
NM_019759	-2.19	0.00005	0.00068	Dpt
NM_001112813	-2.27	0.00005	0.00068	Cacna1g
NM_011338	-2.32	0.00005	0.00068	Ccl9
NM_080639	-2.51	0.00005	0.00068	Timp4

NM_030728	-2.51	0.00005	0.00068	9930013L23Rik
NM_008760	-2.53	0.00005	0.00068	Ogn
NM_023608	-2.62	0.00005	0.00068	Gdpd2
NM_020581	-2.77	0.00005	0.00068	Angptl4
NM_001042615	-2.78	0.00005	0.00068	Htra3
NM_153170	-2.82	0.00005	0.00068	Slc36a2

Suplement Table 3 Hh-signaling differentially responsive genes

Hh-CM vs Esrrb+Hh-CM is statistically different (q<0.05)

Gene bank accession number	Gene Symbol	Control	Hh-CM	Esrrb	Esrrb+Hh-CM
NM_011315	Saa3	1382.76	363.421	2632.83	893.386
NM_011118	Prl2c3	379.645	105.745	687.672	195.874
NM_008276	Hoxd8	32.2431	71.9886	58.1307	149.524
NM_001044751	Hsd11b1	3.95612	83.2878	2.62633	156.776
NM_146116	Tubb4b	58.1223	89.1899	77.0267	159.169
NM_010517	Igfbp4	24.0561	81.7083	18.6476	142.621
NM_011623	Top2a	14.9871	44.2786	21.7656	99.5117
NM_001284507	Crabp1	137.413	62.1162	232.543	113.994
NM_017370	Hp	41.4648	62.5567	40.3169	113.038
NM_054098	Steap4	15.4279	35.4933	41.8127	80.6706
NM_019641	Stmn1	35.9876	72.0775	49.3099	116.755
NM_026785	Ube2c	23.4463	44.0101	26.7943	80.2832
NM_025415	Cks2	24.4244	38.3814	31.4891	72.5601
NM_009144	Sfrp2	8.47181	4.06885	90.6511	34.2415
NM_008966	Ptgfr	32.7896	51.1092	50.7009	80.1903
NM_001012273	Birc5	13.4229	29.1083	19.4776	56.0274
NM_009828	Ccna2	14.5706	28.8957	18.4045	55.2587
NM_001271729	Tk1	10.2435	21.0511	13.717	42.6667
NM_023223	Cdc20	11.3334	21.2123	13.1781	41.9055
NM_001253808	Racgap1	18.1094	30.693	18.9131	51.1653
NM_007681	Cenpa	14.0848	32.3276	17.9992	52.6722
NM_001285997	Prc1	11.4492	22.4382	14.0397	42.5162
NM_134117	Pkdcc	10.5465	25.0133	20.4125	44.5321
NM_028870	Cltb	13.2807	33.0279	17.6575	51.709
NM_001131054	Pttg1	10.9923	18.7735	15.9545	37.292
NM_019759	Dpt	77.4622	17.0169	160.435	35.2561
NM_013555	Hoxd9	7.70152	12.4773	13.7086	30.6003
NM_172301	Ccnb1	8.65499	16.2048	10.1651	33.7935
NM_001163763	Tcf19	14.2568	27.1727	17.8747	44.3905
NM_008695	Nid2	54.4263	22.1403	96.212	37.7018
NM_172505	A730008H23Rik	17.6613	27.1762	19.5963	42.0504
NM_021342	Kcne4	7.3513	11.5393	14.1286	25.4446
NM_013538	Cdca3	6.2023	16.2903	8.85753	29.8962
NM_001199123	Spc25	8.4548	17.7626	10.6615	31.3485
NM_001177752	Pfkfb3	1.44702	7.45226	5.21433	20.8522
NM_009104	Rrm2	12.2636	21.6037	15.3878	34.998
NM_019499	Mad2l1	15.2553	25.573	16.9016	38.7528
NM_011496	Aurkb	6.61205	12.6954	8.1655	25.6183
NM_026560	Cdca8	8.33981	16.9662	8.72491	29.5316
NM_001040435	Tacc3	9.93829	18.3717	10.6727	30.8518
NM_001141975	Tpx2	6.82774	13.5202	8.44815	25.8247
NR_033780	2810001G20Rik	8.04778	12.8002	12.3589	24.5246
NM_016692	Incenp	8.33651	13.7915	9.57497	25.2039
NM_134471	Kif2c	5.19041	10.0141	6.30609	21.3537
NM_144526	Fam64a	6.40796	12.9486	8.15955	24.2332
NM_009773	Bub1b	5.64362	9.99851	7.35944	21.2628
NM_008566	Mcm5	10.1526	15.4165	11.6413	26.6487
NM_023284	Nuf2	6.97773	14.2675	9.20804	25.1471
NM_023209	Pbk	7.50398	14.5895	8.39717	25.369
NM_146171	Ncapd2	6.43767	12.8183	7.631	23.1609
NM_029975	Ulbp1	6.59652	10.024	10.2266	19.743
NM_011121	Plk1	3.91676	8.0847	5.04405	17.6332
NM_008021	Foxm1	5.15874	11.0047	6.66217	20.391

NM_010615	Kif11	4.9772	10.4217	6.65913	19.6478
NM_028390	Anln	7.40574	12.0352	8.34586	21.1809
NM_181589	Ckap2l	4.85332	11.304	6.69815	20.3672
NM_144818	Ncaph	4.88254	9.45474	6.97036	18.5085
NM_011497	Aurka	4.72147	8.57015	5.717	17.5218
NM_024184	Asf1b	4.02044	7.60624	6.24004	16.4537
NM_001083188	Lig1	10.0937	15.6708	10.2945	24.0361
NM_011369	Shcbp1	5.14585	10.0263	6.67605	18.3185
NM_145588	Kif22	4.27706	7.16282	5.47062	15.3129
NM_001004140	Ckap2	7.93221	13.2466	8.57911	21.2596
NM_001190717	Dbf4	8.24379	14.1411	10.3237	22.099
NM_001167743	Slfn8	4.43952	7.64206	7.99226	15.4586
NM_027435	Atad2	7.22101	12.711	8.75452	20.4121
NM_001081117	Mki67	2.62498	7.13259	3.60112	14.3121
NM_026412	Knstrn	4.18197	8.54224	5.45924	15.5637
NM_025995	Fbxo5	6.12983	10.938	8.27816	17.8267
NM_024245	Kif23	4.29708	7.90053	4.88319	14.7708
NM_026778	Cthrc1	3.61061	11.9165	6.56327	18.7002
NM_028128	Rfc5	7.41636	11.2861	8.27539	17.9988
NM_172563	Hlf	2.47716	5.2413	6.63521	11.8926
NM_011495	Plk4	6.17554	9.57894	7.3879	16.1505
NM_023294	Ndc80	5.39062	11.6882	6.39909	18.1685
NM_013552	Hmmr	3.82564	9.10929	4.98689	15.4978
NM_019670	Diap3	4.64476	7.98869	7.02255	14.3548
NM_029766	Dtl	5.69484	10.195	7.36267	16.4838
NM_027975	Fam83d	3.15462	8.13212	4.4174	14.1758
NM_001113179	Bub1	3.00824	6.12662	3.76441	11.9502
NM_001122768	Lrrc8d	1.80273	3.2924	4.21507	9.09625
NM_008017	Smc2	5.17318	9.02486	6.29461	14.797
NM_017407	Spag5	3.4597	6.17541	3.78081	11.8739
NM_146208	Neil3	1.88813	5.11904	2.59228	10.709
NM_019438	Ncapg	4.74681	8.65429	5.07499	14.2257
NM_016808	Usp2	1.0894	7.54002	1.56406	13.0368
NM_013733	Chaf1a	5.80523	9.33386	6.84116	14.7471
NM_007634	Ccnf	4.35235	7.41491	5.19433	12.7692
NM_001177625	Ect2	3.92565	7.87449	5.06206	13.1331
NM_009013	Rad51ap1	3.58395	8.98922	4.17847	14.1513
NM_001177867	Sgol2	3.28707	6.23677	4.69337	11.3567
NM_001080158	Cenpm	3.77236	6.52127	3.99093	11.6394
NM_001024139	Adamts15	0.396237	0.725943	4.52673	5.79864
NM_198622	H1fx	3.61498	6.12892	5.11338	11.1665
NM_011234	Rad51	4.66081	7.77432	5.49473	12.7909
NM_008235	Hes1	0.680057	9.04353	0.0588155	3.17691
NM_001085376	Pappa2	7.79788	12.3406	2.57736	5.36725
NM_016917	Slc40a1	6.08488	12.5111	1.5132	3.81684
NM_007729	Col11a1	7.52241	14.5338	3.08727	4.76399
NM_019503	Fxyd1	11.5538	19.2923	3.9421	6.74855
NM_007470	Apod	10.2707	35.7038	7.69312	15.2765
NM_001164724	Il33	23.7629	64.9144	19.1804	28.6884
NM_029568	Mfap4	29.8356	62.0873	11.4344	13.7917
NM_008760	Ogn	165.909	71.6634	106.437	18.4453
NM_022315	Smoc2	77.9563	119.505	63.281	63.8382

Hh-CM vs Esrrb+Hh-CM is not statistically different (q>0.05)

gene	Gene Symbol	Control	Hh-CM	Esrrb	Esrrb+Hh-CM
NM_008380	Inhba	2.79209	16.5248	1.28256	15.89
NM_001168491	Pdcd4	64.3809	124.969	95.9834	123.671
NM_021492	Ap3b2	0.644023	1.3765	1.09738	1.27967

NM_001111274	Igf1	3.51074	9.75599	7.48334	10.2296
