# Supplementary material for: Genes targeted by the Hedgehog-signaling pathway can be regulated by Estrogen related receptor β
Source: BMC Mol Biol. 2015 Nov 23;16:19. doi: 10.1186/s12867-015-0047-3 (PMC4657266; doi:10.1186/s12867-015-0047-3)
Supplement: Supplementary file 2 — 10.1186/s12867-015-0047-3 Genes regulated by either Esrrb or Hh-signaling pathway. Figure S2: Decision tree model of gene sorting. Figure S3: Hh-responsive transcription-related genes. Figure S4: Correlation of qPCR and RNA-seq of top 15 Hh differentially responsive genes. Figure S5: Esrrb-dependent Hh-responsive genes. Figure S6: Glucocorticoid Receptor related genes in response to Hh-CM and Esrrb. [file 12867_2015_47_MOESM2_ESM.pdf]

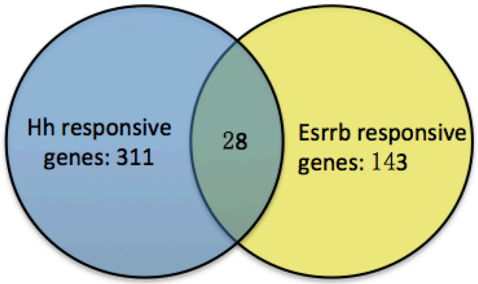

P value=1.24e-18

	Hh increased:	Hh decreased:
Essrb increased:	Igf1(16000)<br>Mafb(16658)<br>Steap4(117167)<br>Pfkfb3(170768)<br>Hlf(217082)	Adam12(11489)<br>Kcnj15(16516)<br>Sfrp2(20319)<br>Stc1(20855)<br>Dpt(56429)<br>ABcc3(76408)<br>Fam110c(104943)<br>Npnt(114249)<br>Prss35(244954)<br>Slc36a2(246049)<br>Zfp808(630579)
Esrrb decreased:	Inhba(16323)<br>Slc40a1(53945)<br>Serpinba1a(66222)<br>Mfab4(76293)<br>Pi15(94227)	Fabp4(11770)<br>Phex(18675)<br>Ccl5(20304)<br>Tagln(21345)<br>Aldh1a7(26358)<br>Lmod1(93689)<br>Ces1a(244595)

Supplement Figure 1. Genes regulated by either Esrrb or Hh-signaling pathway

Hh responsive genes and Esrrb responsive genes were compared, and 28 genes (enrichment p value= 1.24e-18) were altered by either Hh-signaling activation or Esrrb expression. 12 genes among the 28 genes can be regulated the same way by either Hh or Esrrb (Fabp4, Phex, Ccl5, Tagln, Aldn1a7, Lmod1, Ces1a, Igf1, Mafb, Steap4, Pfkfb3, Hlf).

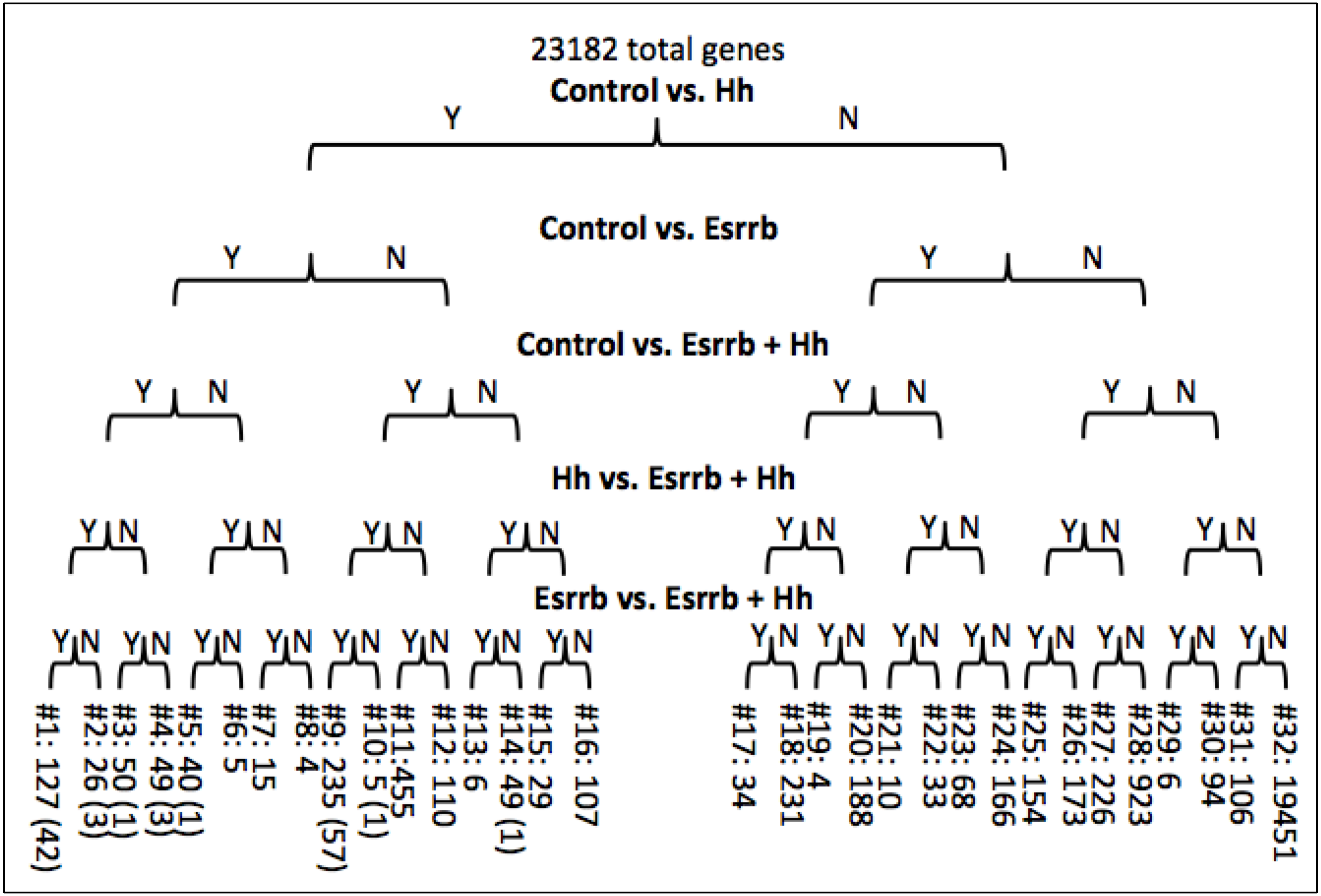
23182 total genes
Control vs. Hh
Control vs. Esrrb
Control vs. Esrrb + Hh
Hh vs. Esrrb + Hh
Esrrb vs. Esrrb + Hh
#1: 127 (42)
#2: 26 (3)
#3: 50 (1)
#4: 49 (3)
#5: 40 (1)
#6: 5
#7: 15
#8: 4
#9: 235 (57)
#10: 5 (1)
#11:455
#12: 110
#13: 6
#14: 49 (1)
#15: 29
#16: 107
#17: 34
#18: 231
#19: 4
#20: 188
#21: 10
#22: 33
#23: 68
#24: 166
#25: 154
#26: 173
#27: 226
#28: 923
#29: 6
#30: 94
#31: 106
#32: 19451

Supplement Figure 2. Decision tree model of gene sorting

For all four biological conditions (control, Hh treatment, Esrrb expression, Esrrb expression with Hh treatment), all 5 possible but non-redundant pairwise comparisons for differentially expressed gene test were carried out. An array including all the transcripts each with the test results of the pairwise comparison was generated, and transcripts are grouped by whether they passed certain test using the “yes (Y)” or “no (N)” logic decision. A total of 32 groups were generated. Genes of interest for further analysis are subjected to further data filter based on fold change value and/or RPKM value.

Gene ID	Symbol	LogFC
Transcription Factor		
14283	Fos11	-1.93
11910	Atf3	-1.67
630579	Zfp808	-1.23
13655	Egr3	-1.09
12394	Runx1	-1.05
71093	Atoh8	1.00
15434	Hoxd3	1.08
217082	Hlf	1.08
52679	E2f7	1.08
14235	Foxm1	1.09
55994	Smad9	1.11
108961	E2f8	1.13
15437	Hoxd8	1.16
16658	Mafb	1.19
16656	Hivep3	1.24
16600	Klf4	1.35
14633	Gli2	1.37
84653	Hes7	1.49
54139	Irf6	1.62
17301	Foxd2	1.71
22061	Trp63	1.87
207259	Zbtb7c	2.26
15205	Hes1	3.73
14632	Gli1	9.14
Chrmomatin Remodeling Factor		
12615	Cenpa	1.20
Transcription co-factor		
12173	Bnc1	-1.59
107765	Ankrd1	-1.19
12189	Brca1	1.05
30937	Lmcd1	1.43
71648	Optn	1.63

Supplement Figure 3. Hh-responsive transcription-related genes

Among the Hh-responsive genes, a group of transcription-related genes are discovered, including transcription factors, chromatin remodeling factors and transcriptional co-factors.

Gene	Control	Shh-CM	Esrrb	Esrrb+shh-CM	Correlation Score
Pdcd4	64.4	125.0	96.0	123.7	0.969
Tubb4b	58.1	89.2	77.0	159.2	0.940
Ogn	165.9	71.7	106.4	18.4	0.998
Dpt	77.5	17.0	160.4	35.3	0.994
Stmn1	36.0	72.1	49.3	116.8	0.998
Hp	41.5	62.6	40.3	113.0	0.981
Hoxd8	32.2	72.0	58.1	149.5	0.996
Top2a	15.0	44.3	21.8	99.5	0.998
Igfbp4	24.1	81.7	18.6	142.6	0.996
Igf1	3.5	9.8	7.5	10.2	0.896
Sfrp2	8.5	4.1	90.7	34.2	0.999
Hsd11b1	4.0	83.3	2.6	156.8	0.999
Saa3	1382.8	363.4	2632.8	893.4	0.996
Smoc2	78.0	119.5	63.3	63.8	0.978
Prl2c3	379.6	105.7	687.7	195.9	0.997

Supplement Figure 4. Correlation of qPCR and RNA-seq of top 15 Hh differentially responsive genes

RPKM (Reads Per Kilo base per Million reads) values of qPCR-validated genes are normalized to NIH3T3-pc3.1. qPCR results from different conditions are also normalized to NIH3T3-pc3.1 condition. For each tested gene, both normalized RPKM values and normalized qPCR results are correlated by Pearson Correlation and correlation coefficients are shown.

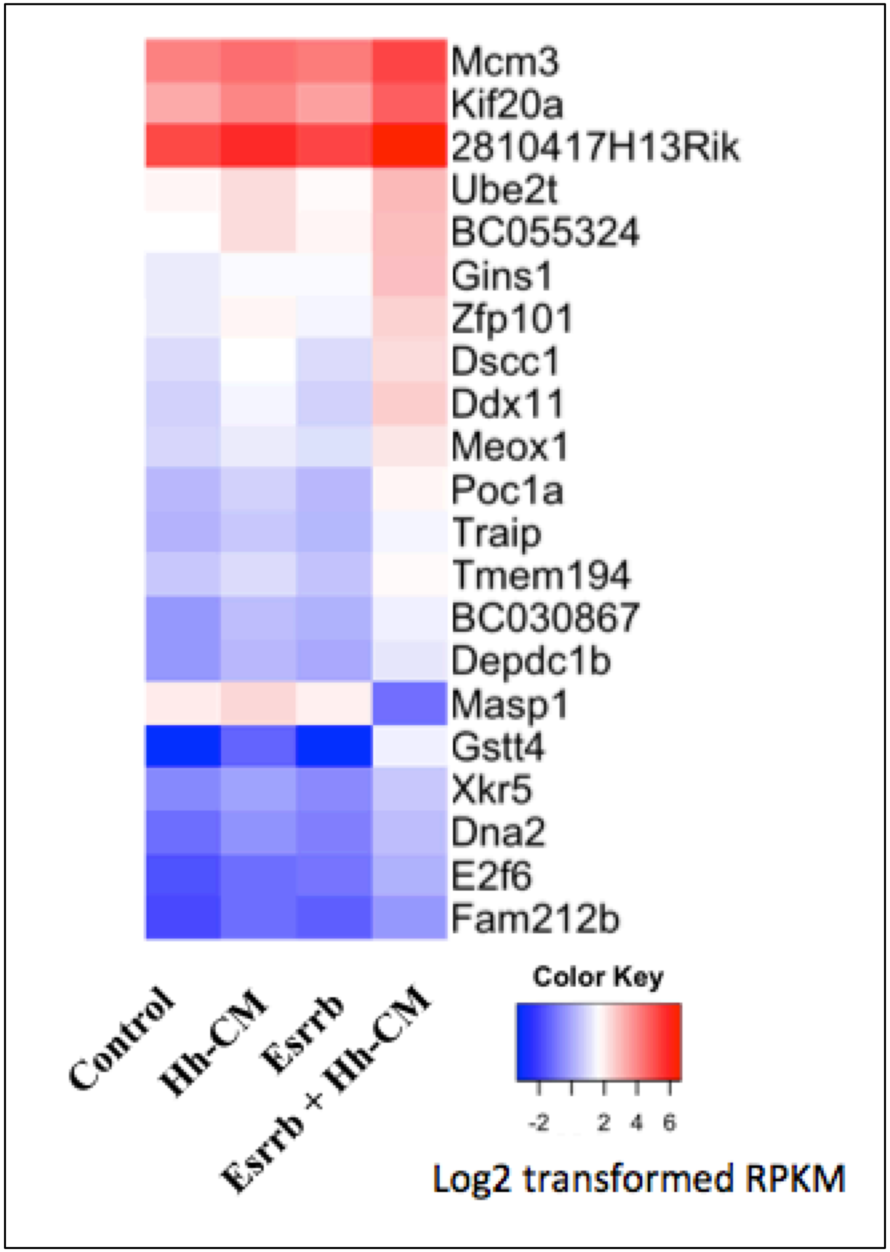
Mcm3
Kif20a
2810417H13Rik
Ube2t
BC055324
Gins1
Zfp101
Dscc1
Ddx11
Meox1
Poc1a
Traip
Tmem194
BC030867
Depdc1b
Masp1
Gstt4
Xkr5
Dna2
E2f6
Fam212b
Control
Hh-CM
Esrrb
Esrrb + Hh-CM
Color Key
-2
2
4
6
Log2 transformed RPKM

Supplement Figure 5. Esrrb-dependent Hh-responsive genes

21 genes showed Hh response only when Esrrb was expressed (Control vs. Hh, q>0.05; Control vs. Esrrb, q>0.05; Control vs. Esrrb+Hh-CM, q<0.05, logFC>1 or <-1; Esrrb vs. Esrrb+Hh-CM, q<0.05, LogFC>1 or <-1; Hh vs. Esrrb+Hh-CM, q<0.05;). The averaged RPKM values of each gene was log2 transformed and further color transformed in heatmap.

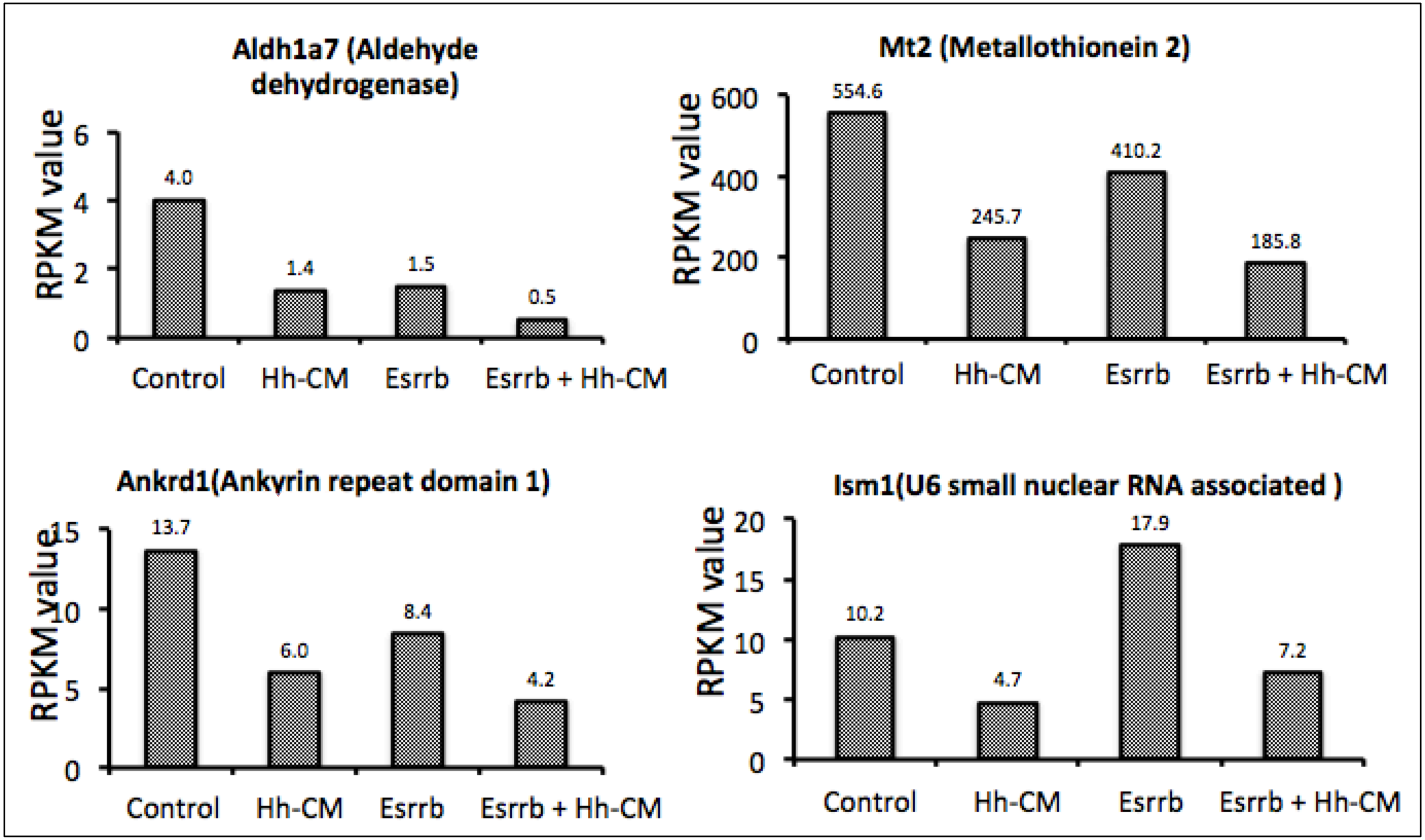
Aldh1a7 (Aldehyde dehydrogenase)
RPKM value
6
4
2
0
4.0
1.4
1.5
0.5
Control
Hh-CM
Esrrb
Esrrb + Hh-CM
Mt2 (Metallothionein 2)
RPKM value
600
400
200
0
554.6
245.7
410.2
185.8
Control
Hh-CM
Esrrb
Esrrb + Hh-CM
Ankrd1(Ankyrin repeat domain 1)
RPKM value
15
10
5
0
13.7
6.0
8.4
4.2
Control
Hh-CM
Esrrb
Esrrb + Hh-CM
Ism1(U6 small nuclear RNA associated)
RPKM value
20
15
10
5
0
10.2
4.7
17.9
7.2
Control
Hh-CM
Esrrb
Esrrb + Hh-CM

Supplement Figure 6. Glucocorticoid Receptor related genes in response to Hh-CM and Esrrb

4 genes having Glucocorticoid Receptor Response Element in their promoter regions were enriched from Hh responsive genes. All four of them, when treated with Hh-CM, were repressed, showed by decreased RPKM value compared Hh to Control. When Hh-CM was treated in the presence of Esrrb, the RPKM values of all 4 genes were further changed compared to Hh-CM treatment without Esrrb expressed.
